# TRIM28 orchestrates SUMO-ubiquitin crosstalk to stabilize PPARG and drive bladder cancer progression

**DOI:** 10.1038/s41419-026-08745-7

**Published:** 2026-04-13

**Authors:** Xuefeng Fan, Zexuan Li, Qiongqiong Gao, Peizheng Huang, Zheyu Li, Dongmei Lu, Lei Wang, Ping Xiang, Tao Huang, Dexin Shen, Jun Xiao

**Affiliations:** 1 Department of Urology, The First Affiliated Hospital of USTC, Division of Life Sciences and Medicine, University of Science and Technology of China, Hefei, China; 2 Core Facility Center for Medical Sciences, The First Affiliated Hospital of USTC, University of Science and Technology of China, Hefei, China

**Keywords:** Bladder cancer, Sumoylation

## Abstract

Bladder cancer (BLCA) is a growing health burden with rising incidence and limited therapeutic options. To define the role of the Tripartite Motif (TRIM) family in BLCA, we integrated multi-cohort transcriptomic analyses with functional and mechanistic validation. TRIM28 was identified as the most consistently upregulated TRIM member in BLCA and correlated with poor prognosis. TRIM28 depletion suppressed, whereas its overexpression enhanced, BLCA cell proliferation. Mechanistically, TRIM28 directly bound to PPARG and acted as a SUMO E3 ligase to catalyze SUMOylation of PPARG at Lys94 within a noncanonical YKYD motif. This modification impaired PPARG recognition by the E3 ubiquitin ligase STUB1, reduced ubiquitin-proteasome degradation, and stabilized PPARG protein. Stabilized PPARG transcriptionally activated cholesterol biosynthetic genes, including *DHCR7* and *DHCR24*, reprogramming cholesterol metabolism to promote BLCA progression. In summary, we identify a TRIM28-PPARG SUMO-ubiquitin crosstalk axis that drives metabolic remodeling and tumor growth in BLCA, highlighting TRIM28-mediated PPARG SUMOylation as a potential therapeutic target for metabolic intervention.

## Introduction

Bladder cancer (BLCA) is one of the most common malignancies of the human urinary system, ranking sixth among all cancers worldwide in annual incidence. Its high recurrence rate and frequent development of chemoresistance remain major clinical challenges. According to the 2023 European Association of Urology (EAU) guidelines, cisplatin-based neoadjuvant chemotherapy combined with radical cystectomy remains the standard treatment for muscle-invasive bladder cancer (MIBC). However, ~30–40% of patients are ineligible for or resistant to cisplatin, leading to limited therapeutic efficacy. In recent years, emerging therapies such as immune checkpoint inhibitors (ICIs) and antibody-drug conjugates (ADCs) have become important treatment options for advanced or metastatic BLCA, partially overcoming the limitations of conventional chemotherapy. Nevertheless, due to significant intertumoral heterogeneity and variable patient responses, only a subset of individuals benefits from these approaches. Overall, current therapeutic strategies have not achieved substantial improvement in overall survival among BLCA patients. Therefore, it remains imperative to identify novel therapeutic strategies and molecular targets that can enhance chemosensitivity, providing new theoretical and translational foundations for improving the clinical management of BLCA.

The tripartite motif (TRIM) protein family is present in virtually all multicellular organisms. TRIM proteins are defined by three conserved N-terminal domains arranged from the N to the C terminus as follows: a RING finger domain, one or two B-box domains, and a coiled-coil region [1, 2]. In addition, they contain a variable C-terminal domain; accordingly, TRIM proteins are also referred to as RBCC proteins. Variation within the C-terminal region accounts for the principal structural diversity among TRIM family members. TRIM proteins are broadly involved in fundamental biological processes such as DNA replication, transcription, translation, post-translational modification, cell-cycle control, and intracellular signal transduction. Dysregulation of TRIM protein function is closely associated with inflammation, immune disorders, degenerative diseases, and tumorigenesis [3, 4]. Owing to the presence of the RING domain, most TRIM proteins are classified as E3 ubiquitin ligases. TRIM24 binds to p53 and promotes its ubiquitination, thereby diminishing the transcriptional activity of p53 and reducing its expression [5]. Elevated TRIM24 levels have been documented in multiple tumor types, including breast and prostate cancers and glioblastoma. Chu et al. further reported that a subset of TRIM proteins act as SUMO E3 ligases; for example, TRIM27 catalyzes SUMOylation of MDM2, which suppresses MDM2 ubiquitination, enhances its stability [6]. In BLCA, several members of the TRIM (Tripartite Motif) protein family have also been reported to play important oncogenic roles. For instance, TRIM65 has been shown to enhance the invasiveness of BLCA cells by promoting the ubiquitination and degradation of ANXA2 [7], while TRIM26 facilitates tumor progression through activation of the Akt/GSK3β/β-catenin signaling pathway [8]. Despite these findings, no comprehensive study has yet systematically characterized the expression patterns and prognostic relevance of TRIM family genes in BLCA. Therefore, in this study, we aimed to perform an integrative bioinformatics analysis using publicly available datasets to delineate the expression landscape of TRIM family members in BLCA. By identifying the TRIM protein most closely associated with disease progression and poor patient prognosis, we sought to provide new mechanistic insights into how TRIM family proteins contribute to BLCA development and to establish a theoretical foundation for future therapeutic exploration.

Peroxisome proliferator-activated receptor gamma (PPARG) is a multifunctional nuclear receptor that plays critical roles in regulating metabolism, controlling inflammation, alleviating atherosclerosis, inhibiting tumorigenesis, and modulating immune responses. As a key transcription factor in lipid metabolism, PPARG governs the expression of genes involved in fatty acid and cholesterol homeostasis [9]. Recent studies have highlighted the importance of PPARG-mediated lipid metabolic reprogramming in cancer progression. For instance, an oleic acid–PPARG–FABP4 signaling axis has been shown to promote lymph node metastasis in cholangiocarcinoma, and targeting PPARG-driven lipid remodeling may help reduce metastatic burden and disease progression [10]. Similarly, in esophageal adenocarcinoma, tumor-specific MRTFs activate PPARG to enhance de novo synthesis of fatty acids, phospholipids, and sphingolipids, thereby facilitating tumor cell proliferation [11]. The protein stability and transcriptional activity of PPARG are tightly regulated by multiple post-translational modifications. SIRT1 deacetylates PPARG at Lys268 and Lys293, promoting its degradation [12]. In hepatocellular carcinoma, USP22 stabilizes PPARG through deubiquitination, leading to upregulation of key lipogenic enzymes such as ACLY and ACC and promoting malignant progression [13]. Additionally, ERK and CDK5 cooperatively mediate phosphorylation of PPARG at Ser273, and pharmacological targeting of this phosphorylation site has become a major strategy for the development of PPARG modulators [14]. Collectively, these findings suggest that elucidating the post-translational regulatory mechanisms of PPARG in the context of BLCA will provide critical insights into how PPARG signaling contributes to tumor metabolic reprogramming and malignant progression.

In this study, we use machine learning-based methods to identify TRIM28 as closely linked to the progression and prognosis of BLCA. We show that TRIM28 drives BLCA progression by stabilizing PPARG, thereby augmenting cholesterol biosynthesis in BLCA cells. Mechanistically, we demonstrate a direct interaction between TRIM28 and PPARG whereby TRIM28 enhances SUMOylation of PPARG at Lys94, which in turn suppresses STUB1-mediated proteasomal degradation of PPARG. Taken together, we uncover an innovative regulatory axis in which TRIM28 orchestrates the balance between SUMOylation and ubiquitination to control PPARG stability, providing a new mechanistic foundation for understanding TRIM28 function in BLCA and the post-translational regulation of PPARG.

## Materials and methods

### Cell culture

The cell lines and culture conditions employed in this study are consistent with those reported in our previous study, as previously reported [15]. All cell lines used in this study are obtained from the Cell Bank of the Chinese Academy of Sciences and were authenticated using Short Tandem Repeat (STR) profiling. T24 BLCA cells are maintained in the 1640 medium, UM-UC3 BLCA cells in the MEM medium, and HEK293T cells in the DMEM medium. All media are supplemented with 10% FBS (Biochannel, BC-SE-FBS08, China) and 1% penicillin-streptomycin (Biochannel, BC-CE-007, China).

### Nucleic acid transfection

All steps are performed as previously reported [15]. KeygenMAX3000 Reagent (KGA9705-1.5, KeyGen BioTECH) are used for nucleic acid transfection. For plasmid transfection, 1 µg of plasmid DNA is mixed with 5 µL MAX3000 reagent and 200 µL Opti-MEM for about 3 × 10^5^ cells. The information of siRNAs is listed in Supplementary Table 1. His SUMO1/2/3 plasmids, V5-Ubc9 plasmids, Flag-TRIM28 plasmid and related TRIM28-truncated plasmids are obtained from Juyan Biotechnology Co., Ltd., Hefei, China. His-Ub plasmid, PPARG-related E3 ligase plasmids, HA-PPARG plasmid and related PPARG-truncated or mutant plasmids are procured from Miaoling Co., Ltd., Wuhan, China.

### RNA extraction and qRT-PCR

All steps are performed as previously reported [15]. Total RNA is extracted using the Sparkjade SPARKeasy Cell RNA Kit (AC0205-B) in accordance with the user instructions. qRT-PCR is performed using the TIANGEN FastReal SYBR Green Kit (FP217-01). The information of primers is listed in Supplementary Table 2.

### Western blot (WB) and co-immunoprecipitation (Co-IP)

All steps are performed as previously reported [15]. Generally, cells are collected and lysed with RIPA buffer (BI-WB013, SBJbio) containing 1 mM PMSF (BI-WB081, SBJbio). Proteins are resolved via SDS-PAGE (GF1810, GeneFist, China). Co-IP assays are performed via using the BeaverBeads™ Protein A/G Immunoprecipitation Kit (22202-100, Beaver, China). Secondary antibodies (Abbkine, A25022) specific to avoid cross-reactivity are used for Co-IP and corresponding WB analysis. The information of antibodies is listed in Supplementary Table 3.

### Cell proliferation assays

Cell Counting Kit-8 (CCK-8) (SparkJade, CT0001-E, China) are used. About 3000 cells with 200 µL are seeded into 96-well plates per well, and 20 µL of CCK-8 reagent is added at different time points (0, 1, 2, 3 and 4days). A microplate reader (Beckman, Brea, CA, USA) is used to obtain the cell absorbance at 450 nm after 3 h of incubation at 37 °C. For clone formation assays, transfected cells (approximately 500 or1000 per well) are seeded in a 6-well plate and cultured for 10 days. Each well is fixed with 4% paraformaldehyde (PFA) for 30 min and stained with 0.1% crystal violet solution for 30 min.

### Cholesterol measurement

Intracellular cholesterol level is measured by Amplex Red Cholesterol Assay Kit (Beyotime, S0211S) according to the user instructions. Briefly, cells are collected and lysed by assay buffer. Corresponding agent is added with subsequent incubation. Absorbance of 570 nm is detected.

### Flow cytometry assays

Cell apoptosis is evaluated using the Annexin V-FITC/PI Apoptosis Detection Kit (A5001-02P, Simubiotech, China). All steps are performed according to the user instruction. The apoptosis rate of BLCA cells are detected by flow cytometry (FACSFortessa, BD, USA).

### Chemicals

Details of all chemicals and their working concentrations are provided in Supplementary Table 4.

### Data collection and preprocessing

Four public datasets are included: TCGA-BLCA (RNA-seq; log_2_(TPM + 1)), GSE13507 (microarray; log₂-transformed, quantile-normalized), GSE32894 (microarray; log_2_-QN), and UROMOL (RNA-seq; log_2_(TPM + 1)). Duplicate gene symbols are removed by retaining the entry with the highest expression value. Only mRNA data with matched clinical information are used. For intra-cohort normalization, microarray matrices are processed using normalizeBetweenArrays in the limma package. When necessary, multi-batch integration within the same platform is corrected using sva/ComBat (restricted to homogeneous training datasets; external validation cohorts are processed independently). Differential expression analysis is performed with limma, using thresholds of |log₂FC| ≥ 0.58 and FDR (Benjamini–Hochberg) < 0.05. All statistical and visualization analyses are conducted in R v4.3.2.

### Machine learning-based biomarker selection

Five algorithms are independently applied in parallel to the same candidate set: LASSO (glmnet, α = 1, λ determined by 10-fold cross-validation), Random Forest (randomForestSRC::rfsrc, ntree = 1000, nodesize = 50, permutation-based importance, 1000 Monte Carlo repetitions), XGBoost (xgboost, logistic loss, 5-fold cross-validation with early stopping; parameter ranges: eta = 0.05-0.1, max_depth = 3-7, subsample = 0.8, colsample_bytree = 0.8), Boruta (shadow feature thresholding, doTrace = 2, ntree = 1000, output: Confirmed/Rejected), and SVM-RFE (caret + e1071, linear kernel, 10-fold cross-validation). All models are trained exclusively on the “tumor vs. normal” framework of TCGA-BLCA, while external GEO cohorts are used only for expression and diagnostic validation, not for model training or tuning. The final robust feature set is obtained by integrating the results of all five methods using UpSet and Venn intersection analyses.

### Visualization and cross-cohort consistency assessment

Expression differences are visualized using violin + box + jitter plots, annotated with sample sizes per group and titled as “Dataset-Variable.” Integrated heatmaps are generated with ComplexHeatmap, where samples are grouped by NMIBC/MIBC and ordered by TRIM28 expression within each group. Top annotations included Type, Grade, N stage, and Overall Survival. To assess cross-cohort consistency, triangular heatmaps are constructed: each cell (Dataset × Gene) displayed the direction of median difference in the upper triangle (whether tumor/high-grade > normal/low-grade) and the significance (*P* < 0.05, Welch/Wilcoxon) in the lower triangle. Kaplan–Meier plots annotated HR and 95% CI; when survival curves intersected between 730 and 1095 days, the crossover point is recorded and truncated plots are redrawn to validate early-stage robustness.

### CRISPR/Cas9 sgRNA cloning

Single-guide RNA (sgRNA) targeting TRIM28 is designed using CRISPOR (https://crispor.gi.ucsc.edu/crispor.py). Complementary oligonucleotide is synthesized by General Bio (Anhui) Co., Ltd. Oligos are phosphorylated/annealed and ligated into lentiCRISPR v2 previously linearized with Esp3I (BsmBI) (Yeasen Biotechnology, FuniCut™ Esp3I, 15048ES30). Ligation is performed with T4 DNA ligase (Yeasen Biotechnology, Hieff® Gold T4 DNA Ligase, 10300ES80). Ligation mixtures are transformed into Stbl3 chemically competent E. coli and plated on selective medium. Positive colonies are screened and verified by Sanger sequencing (General Bio, Anhui).

### RNA-seq analysis

Cells are collected after silencing TRIM28. Total RNA is extracted as described above. Subsequent steps are performed as previously reported [16]. RNA-seq data is uploaded to GEO database, and the GEO accession number is GSE318442.

### LC/MS analysis for TRIM28-interacted proteins

HEK-293T cells are transfected with Flag-TRIM28 plasmid. Cells are collected and lysed for immunoprecipitation steps as described above. Subsequent steps are performed as previously reported [15]. Proteins that interact with Flag-TRIM28 is provided as supplementary information.

### In vivo experiments and immunohistochemistry (IHC)

The Ethics Committee for Experimental Animals at The First Affiliated Hospital of the University of Science and Technology of China approved this study (Approval Number: 2025-N(A)-176).

Male BALB/c nude mice, purchased from Zhejiang Vital River Laboratory Animal Technology Co., Ltd., are randomly assigned to experimental groups. A total of 5 × 10^6^ sgNC or sgTRIM28 T24 cells are subcutaneously injected into the mice. Each xenograft is measured every 3 days starting six days post-injection, and tumor volume is calculated using the formula V = (length × width^2^) × 0.5. All mice are sacrificed on day 30 following xenografting, after which tumor weight and volume are recorded. IHC assays are conducted as previously described [15]. Sample sizes were chosen based on prior experience and published xenograft studies in the field. No samples or animals were excluded from the analysis. Investigators were not blinded to group allocation during the experiment, outcome assessment, or data analysis.

### Statistical analysis

Two-group comparisons are conducted using two-sided Wilcoxon tests (or Welch’s *t*-test when normality and homoscedasticity are satisfied). For ≥3 groups, Kruskal–Wallis tests with Dunn’s post hoc and Benjamini–Hochberg (BH) correction are applied. Multiple testing adjustments are consistently handled using BH correction. Diagnostic performance is evaluated using pROC (DeLong confidence intervals/comparisons) and timeROC (standardized, marginally weighted). Survival analysis is performed by Kaplan–Meier and log-rank tests, and independent prognostic effects are assessed by Cox regression (reporting HR, 95% CI, and FDR). Results from three independent experiments are expressed as mean ± standard deviation (SD). Significance symbols: ns ≥ 0.05; ^*^P < 0.05; ^**^P < 0.01; ^***^P < 0.001.

## Results

### Identification of TRIM28 as the core TRIM family member associates with BLCA progression

**Fig. 1:**
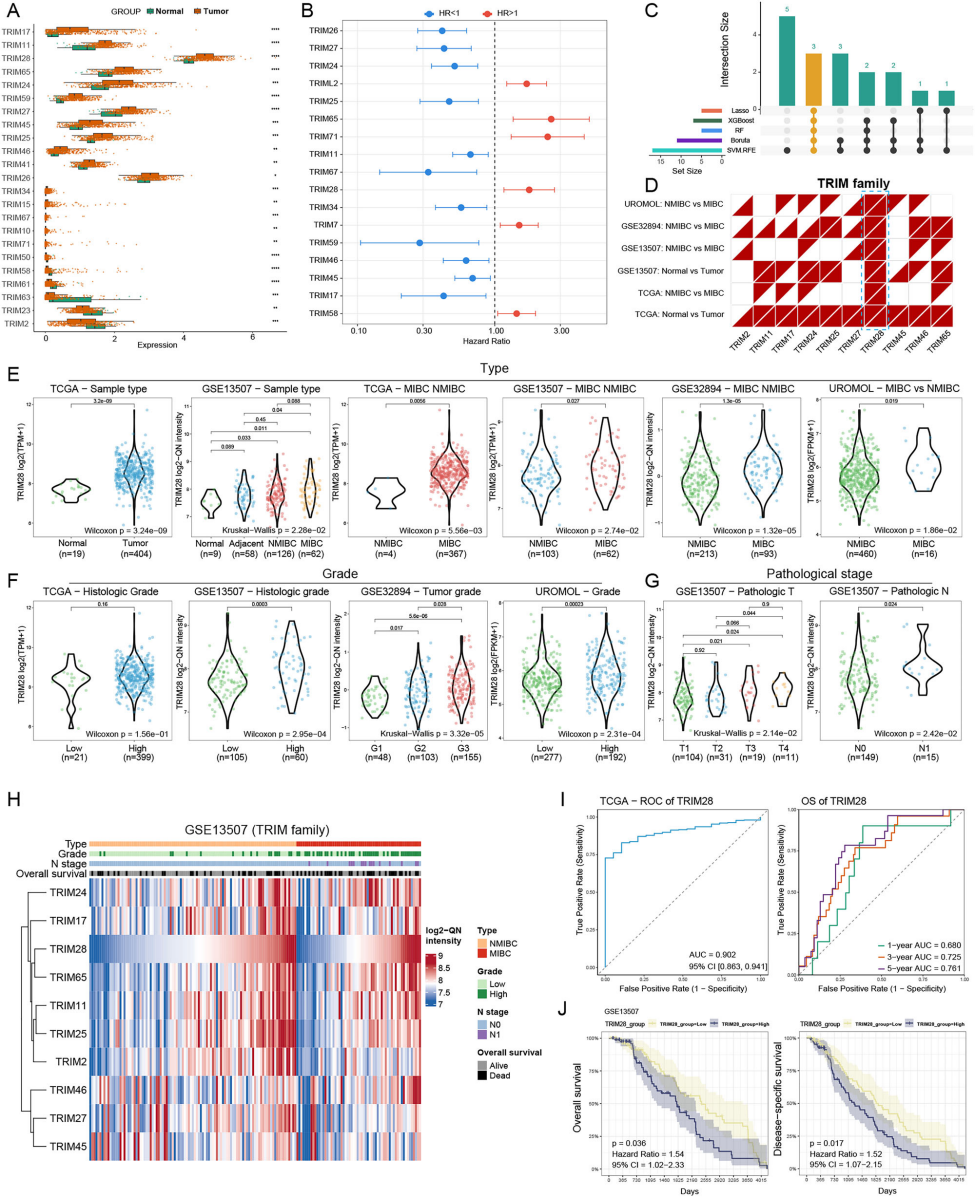
Cross-cohort expression profiling, feature selection, and diagnostic/prognostic value of TRIM28 in bladder cancer. **A** Overview boxplots showing differential expression of TRIM family genes between tumor and normal tissues in the TCGA-BLCA cohort. **B** Forest plot of univariate Cox regression displaying the association between TRIM gene expression and OS. **C** UpSet plot showing the intersection of top-ranked candidate genes identified by five independent feature selection algorithms (LASSO, random forest, XGBoost, Boruta, and SVM-RFE). **D** Triangular heatmap summarizing the expression differences of ten TRIM genes (TRIM2, 11, 17, 24, 25, 27, 28, 45, 46, 65) across normal, NMIBC, and MIBC tissues in four cohorts. Boxplots showing stepwise elevation of TRIM28 expression across BLCA subtypes (**E**), pathological grades (**F**), and increasing T and pathological stages (**G**). **H** Integrated heatmap of TRIM family expression and clinical annotations in the GSE13507 cohort. **I** Diagnostic and time-dependent ROC curves of TRIM28 for distinguishing NMIBC and MIBC and predicting OS. **J** Kaplan-Meier curves of OS and DSS comparing high versus low TRIM28 expression groups. ^*^ *P* < 0.05, ^**^ *P* < 0.01, ^***^ *P* < 0.001.

Using four independent cohorts (TCGA-BLCA, GSE13507, GSE32894, and UROMOL), we systematically analyze TRIM family expression and prognosis in bladder cancer. In the TCGA cohort, 23 TRIM genes are differentially expressed between tumors and normal tissues (18 upregulated, 5 downregulated; Fig. 1A). Multigene survival analysis reveals heterogeneity among family members, with several, including TRIM28, showing a significant association with poor overall survival (OS) (Fig. 1B). To identify robust diagnostic candidates, five machine-learning algorithms (XGBoost, random forest, Boruta, LASSO, and SVM-RFE) are applied in parallel. Independent feature sets are integrated using UpSet and Venn analyses (Fig. 1C & Supplementary Fig. 1), yielding three consistent genes: TRIM27, TRIM65, and TRIM28. Among ten TRIM members detectable across all four datasets, cross-cohort comparisons reveal higher expression of TRIM25, TRIM28, TRIM46, and TRIM65 in tumor or MIBC tissues, whereas TRIM2 is enriched in normal or NMIBC samples. Notably, TRIM28 is the only gene consistently and significantly upregulated across all datasets (Fig. 1D & Supplementary Fig. 2), and is thus selected for further study. Clinical stratification confirms that TRIM28 expression increases progressively with malignancy-from normal to NMIBC to MIBC (Fig. 1E), and is higher in high-grade, advanced-stage, lymph node-positive tumors and worse prognosis (Fig. 1F, G & Supplementary Fig. 3A, B & Table 1). Integrated heatmaps ordered by TRIM28 levels show co-occurrence of high TRIM28 with MIBC status, higher histological grade, nodal metastasis, and poorer survival (Fig. 1H). Receiver operating characteristic (ROC) analyses demonstrate that TRIM28 effectively distinguishes MIBC from NMIBC, with AUCs of 0.902, 0.730, 0.657, and 0.673 in TCGA, GSE13507, GSE32894, and UROMOL, respectively (Fig. 1I; Supplementary Fig. 3C). Time-dependent ROC curves further show improving long-term predictive accuracy for OS (1-/3-/5-year AUC = 0.680/0.725/0.761; Fig. 1I). Kaplan-Meier analysis confirms that high TRIM28 expression predicts worse OS (HR = 1.54, 95% CI 1.02-2.33) and DSS (HR = 1.52, 95% CI 1.07-2.15; Fig. 1J).

**Table 1:** Clinicopathological statistics of BLCA patients from GSE13507 based on TRIM28 expression level.

Clinicopathological Features	TRIM28 expression level		Total	*P* value
	Low	High		
Age				
<65	40 (48.19%)	29 (35.37%)	69	0.0434^*^
≥65	43 (51.81%)	53 (64.63%)	96	
Grade				
Low	62 (74.70%)	43 (52.44%)	105	<0.001^***^
High	21 (25.30%)	39 (47.56%)	60	
Invasiveness				
Non-muscle invasive	58 (69.88%)	45 (54.88%)	103	0.0274^*^
Muscle invasive	25 (30.12%)	37 (45.12%)	62	
M stage				
M0	80 (96.39%)	78 (95.12%)	158	0.642
M1 + MX	3 (3.61%)	4 (4.88%)	7	
N stage				
N0	81 (97.59%)	68 (83.95%)	149	0.0242^*^
N1 + N2 + N3 + NX	2 (2.41%)	13 (16.05%)	15	
Disease-Specific Survival (DSS)				
Alive	10 (12.05%)	22 (26.83%)	32	0.0136^*^
Dead	73 (87.95%)	60 (73.17%)	133	

^*^P < 0.05; ^**^P < 0.01; ^***^P < 0.001. Bold values indicate statistically significant differences. (*P* < 0.05).

Collectively, multi-cohort validation and multi-algorithm feature selection consistently identify TRIM28 as the most critical TRIM family member in BLCA. TRIM28 expression increases with tumor aggressiveness and exhibits stable diagnostic and prognostic performance, providing a strong rationale for its in-depth functional characterization in subsequent experiments.

### TRIM28 is essential for BLCA progression

**Fig. 2:**
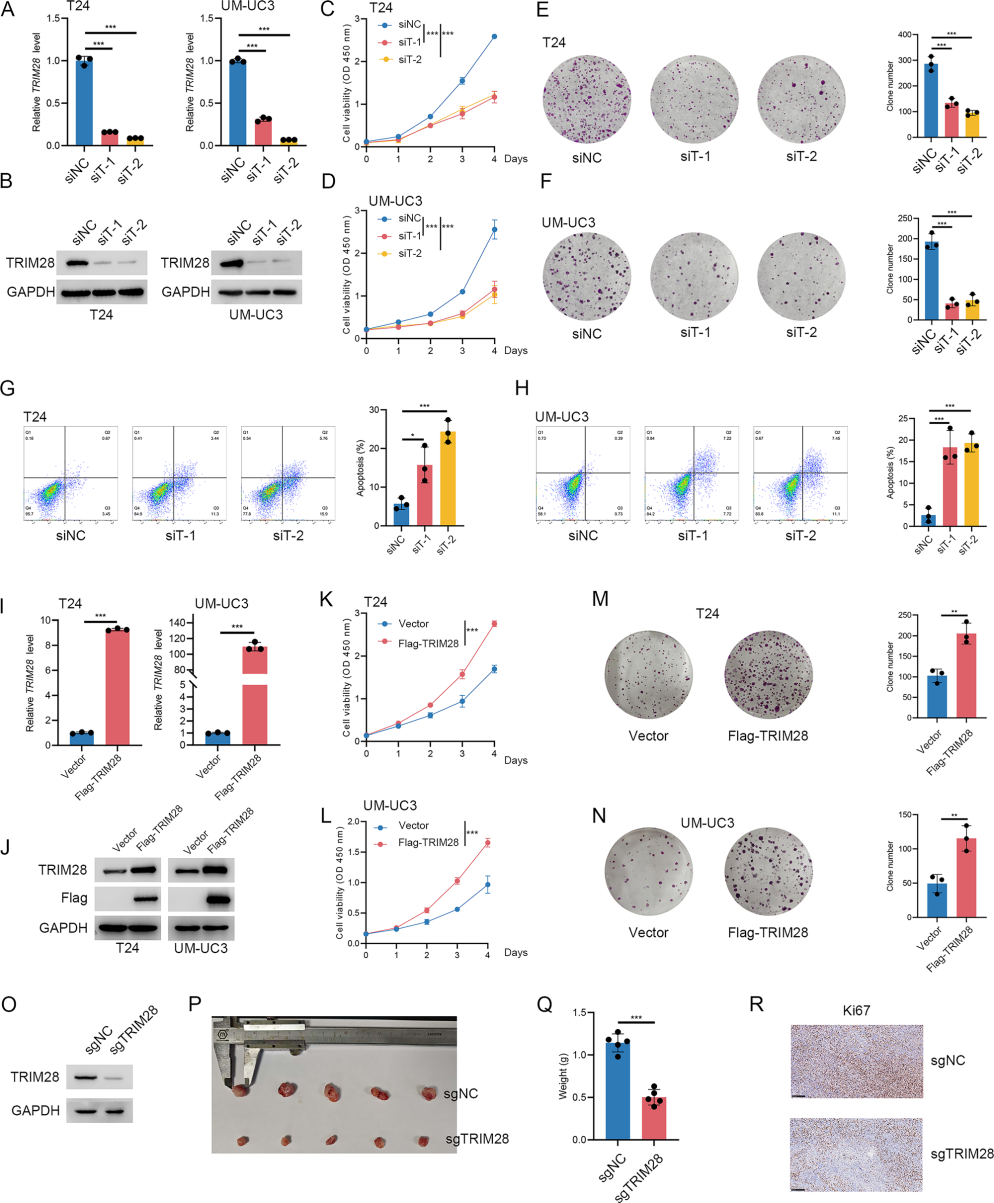
TRIM28 is essential for BLCA cell proliferation. Validation of TRIM28 knockdown efficiency in T24 and UM-UC3 cells by qRT-PCR (**A**) and Western blot (**B**). **C**, **D** CCK-8 assays showing that TRIM28 silencing significantly reduces cell viability in T24 and UM-UC3 cells. **E**, **F** Colony formation assays demonstrating decreased clonogenic ability after TRIM28 knockdown. Representative images and quantification are shown (right). **G**, **H** Flow cytometry analysis indicates that TRIM28 depletion increases apoptosis rates in T24 and UM-UC3 cells. Verification of TRIM28 overexpression in T24 and UM-UC3 cells by qRT-PCR (**I**) and Western blot (**J**). **K**, **L** CCK-8 assays showing that TRIM28 overexpression promoted proliferation in both cell lines. **M**, **N** Colony formation assays confirming the pro-proliferative effect of TRIM28 overexpression. **O**-**R** In vivo xenograft model using T24 cells with or without TRIM28 knockout. **O** Western blot validation of TRIM28 deletion. **P**, **Q** Representative tumor images and tumor weights. **R** Immunohistochemical staining for Ki-67 showing decreased proliferation in sgTRIM28 tumors.

After identifying TRIM28 as the most critical TRIM family member associates with malignant progression of bladder cancer through multi-cohort validation and multi-algorithm feature selection, we sought to further elucidate its functional role in tumor development. We first silence TRIM28 in T24 and UM-UC3 bladder cancer cells using two target-specific siRNAs (Fig. 2A, B). Both CCK-8 proliferation assays and colony formation assays demonstrates that TRIM28 knockdown markedly reduces the proliferative capacity of bladder cancer cells (Fig. 2C–F). Flow cytometric analysis further reveals a significant increase in apoptotic cell populations following TRIM28 depletion (Fig. 2G, H). Conversely, ectopic overexpression of Flag-tagged TRIM28 (Fig. 2I, J) notably enhances cell proliferation and clonogenic growth (Fig. 2K–N), confirming its pro-tumorigenic effect in vitro. To validate these findings in vivo, we generate TRIM28-knockout T24 cells (Fig. 2O) using CRISPR/Cas9 and perform subcutaneous xenograft assays in nude mice. The xenograft results show that tumors derived from TRIM28-deficient cells exhibit a marked reduction in final tumor weight (Fig. 2P, Q). Immunohistochemical analysis further confirms decreased Ki-67 expression in tumors lacking TRIM28 (Fig. 2R). Collectively, both in vitro and in vivo experiments consistently demonstrate that TRIM28 acts as a key driver of malignant proliferation in bladder cancer.

### TRIM28 regulates BLCA cholesterol metabolism

**Fig. 3:**
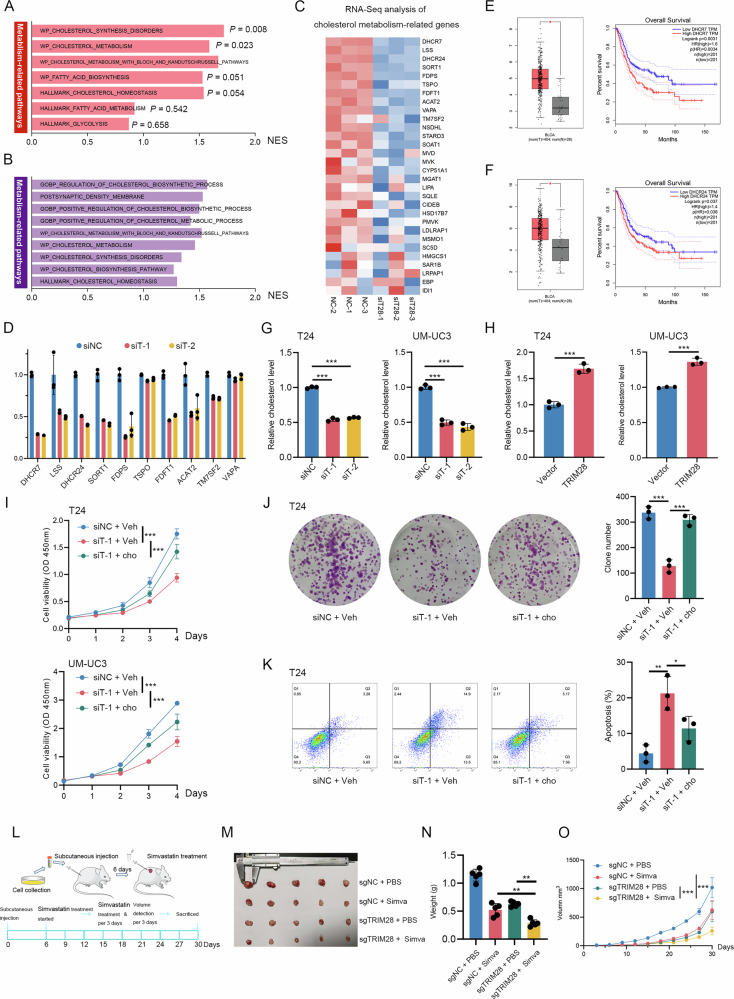
TRIM28 regulates BLCA cholesterol metabolism. **A** Metabolism-related pathway via GSEA analysis conducted on GSE32894. **B** Cholesterol-related pathway via GSEA analysis conducted on TRIM28-silenced RNA-seq. **C** Heatmap of RNA-Seq expression for cholesterol metabolism-related genes. **D** qRT-PCR validation of selected cholesterol pathway genes in UM-UC3 cells after silencing TRIM28. **E**, **F** Clinical association of DHCR7 and DHCR24 in TCGA-BLCA cohort via GEPIA online database. **G** Relative cholesterol in T24 and UM-UC3 cells after silencing TRIM28. **H** Relative cholesterol in T24 and UM-UC3 cells after overexpressing TRIM28. **I** CCK-8 cell viability curves for T24 (top) and UM-UC3 (bottom) cells with siRNA and exogenous cholesterol (cho) treatment as indicated. **J** Representative colony formation assays (left) and quantification (right) for T24 cells. **K** Flow cytometry apoptotic analysis for T24 cells under the same conditions. **L** Drug treatment regimens of in vivo experiments. Representative tumor images (**M**) and quantification of tumor weights (**N**) and tumor volume (**O**). ^*^P < 0.05, ^**^P < 0.01, ^***^P < 0.001.

To explore the molecular mechanisms underlying the oncogenic role of TRIM28 in BLCA, we first perform gene set enrichment analysis (GSEA) based on public transcriptomic dataset (GSE32894). The analysis reveals that high TRIM28 expression is significantly associated with pathways related to multiple metabolism-related pathways, including cholesterol metabolism, fatty acid metabolism, and glucose metabolism pathways (Fig. 3A & Supplementary Table 5). To further validate these observations, we conduct RNA-seq in T24 BLCA cells following TRIM28 knockdown. GSEA of the transcriptomic data indicates that depletion of TRIM28 markedly affects multiple cholesterol metabolism-related pathways (Fig. 3B & Supplementary Table 6), accompanied by significant downregulation of several key enzymes involve in cholesterol biosynthesis (Fig. 3C). Consistent findings are observed in UM-UC3 cells, where TRIM28 silencing similarly suppresses the expression of critical enzymes participating in cholesterol metabolism (Fig. 3D). Among these, DHCR7 and DHCR24 show the most pronounced reduction upon TRIM28 knockdown (Fig. 3C & D). Both the two genes are markedly upregulated in BLCA tissues and correlate with unfavorable clinical outcomes (Fig. 3E, F), in agreement with previous studies showing that cholesterol biosynthesis catalyzed by DHCR7 and DHCR24 drives BLCA progression [17, 18]. These results suggest a previously unrecognized role of TRIM28 in modulating cholesterol metabolism. Supporting this notion, TRIM28 knockdown significantly decrease intracellular cholesterol levels (Fig. 3G), whereas TRIM28 overexpression elevates cholesterol content in BLCA cells (Fig. 3H). Moreover, exogenous cholesterol supplementation not only rescues the proliferative inhibition caused by TRIM28 silencing but also alleviate the induced apoptosis by TRIM28 silencing (Fig. 3I-K). Simvastatin, a widely used cholesterol-lowering agent, has been reported to suppress the growth of bladder cancer cells [19]. Treatment of simvastatin further decreases the intracellular cholesterol in sgTRIM28-T24 cells, showing the potential additive inhibitory effect (Supplementary Fig. 4A). To determine whether TRIM28 depletion could potentiate the antitumor effects of simvastatin in vivo, we perform subcutaneous xenograft assays in nude mice (Fig. 3L). The results show that combined treatment with TRIM28 knockout and simvastatin administration produces a markedly stronger inhibitory effect on tumor growth compared with either treatment alone (Fig. 3M). Tumor growth rate and final xenograft weight are both significantly reduced in the TRIM28 knockout + simvastatin group relative to the TRIM28 knockout only or simvastatin only groups (Fig. 3N, O). Collectively, these findings demonstrate that TRIM28 enhances BLCA progression by regulating cholesterol metabolism through the transcriptional upregulation of key biosynthetic enzymes.

### Identification and validation of PPARG as an interacting partner of TRIM28

**Fig. 4:**
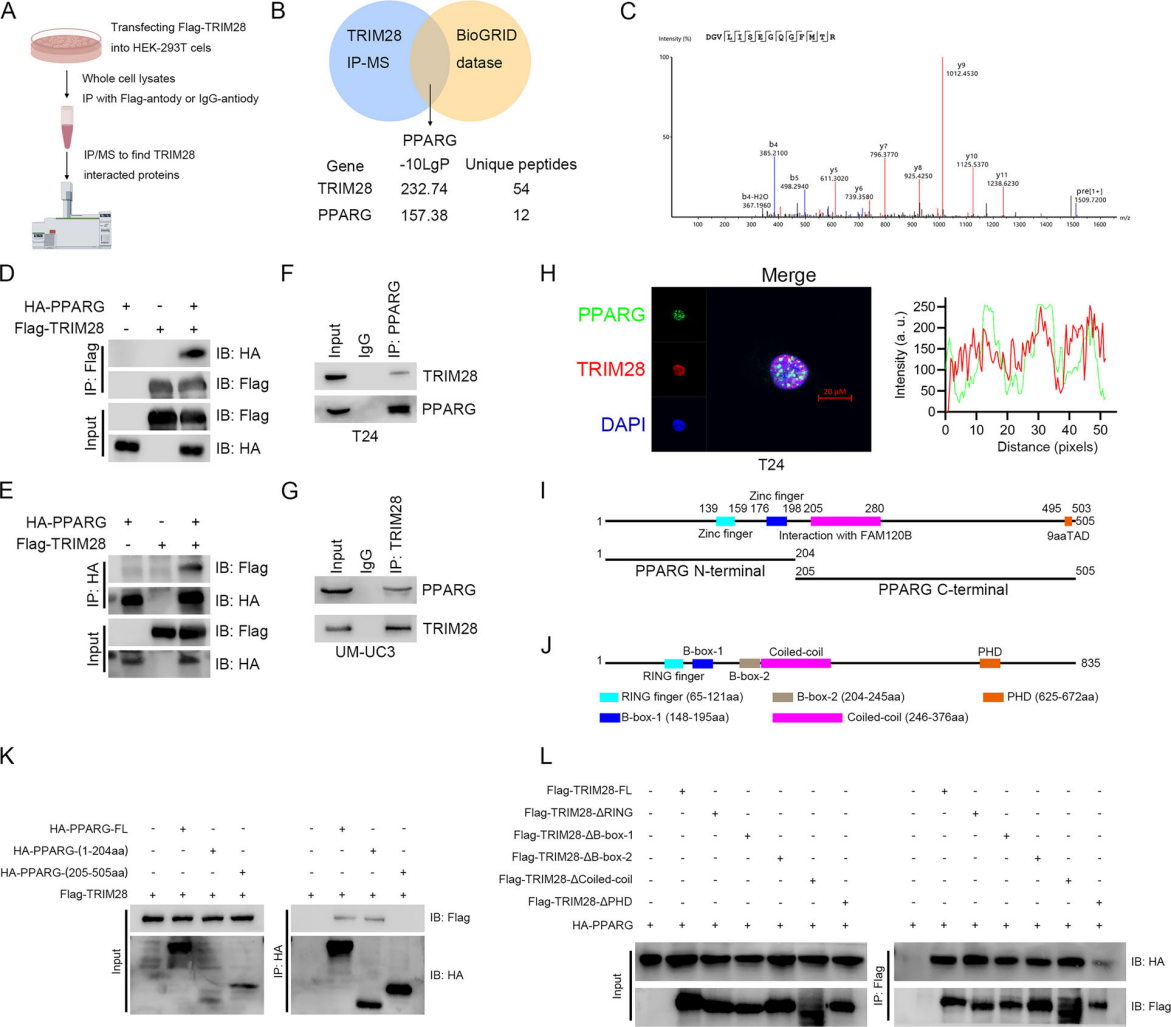
TRIM28 interacts with PPARG. **A** Workflow of TRIM28 interactome identification. **B** Venn diagram integrating TRIM28 IP–MS candidates with the BioGRID database highlights PPARG. **C** Representative MS spectrum of a PPARG peptide detected in the TRIM28 IP/MS. **D**, **E** Exogenous Co-IP assays for detecting the interaction between TRIM28 and PPARG. **F**, **G** Endogenous Co-IP assays for detecting the interaction between TRIM28 and PPARG in T24 and UM-UC3 cells. **H** Immunofluorescence colocalization of PPARG (green) and TRIM28 (red) with DAPI (blue). Scale bar, as indicated. **I** Schematic of PPARG domain architecture and truncation constructs used for mapping. **J** Schematic of TRIM28 domain architecture and deletion mutants employed for mapping. **K** Mapping of the PPARG region required for TRIM28 association. **L** Mapping of the TRIM28 region required for PPARG association.

Next, we try to investigate how TRIM28 regulates cholesterol metabolism. We transfect Flag-TRIM28 plasmid in HEK-293T cells and conduct an IP-MS assay to screen potential TRIM28-interacted proteins (Fig. 4A). We further intersect the proteins acquired by IP-MS with previously reported TRIM28-interatced proteins via exploring BioGrid database (Fig. 4B). Among these, PPARG is prioritized due to its role in regulating lipid metabolism [13, 20, 21], its well-known function in promoting BLCA progression [22] and the high score in our IP-MS results (Fig. 4B, C). We also check whether PPARG could regulate the cholesterol metabolism of BLCA cells. Our qRT-PCR results show silencing PPARG and pharmacological inhibiting PPARG significantly reduce the mRNA level of cholesterol metabolism genes (Supplementary Fig. 4B, C), and the intracellular cholesterol content is also decreased in PPARG silenced cells (Supplementary Fig. 4D). Therefore, we aim to explore the potential regulation of TRIM28 on PPARG.

We firstly explore the potential interaction between TRIM28 and PPARG. We co-transfect Flag-TRIM28 plasmid and HA-PPARG plasmid in HEK-293T cells and perform exogenous Co-IP assays. Our results confirm the interaction between TRIM28 and PPARG (Fig. 4D, E). Endogenous Co-IP assays in T24 and UM-UC3 BLCA cells further validate the interaction mode (Fig. 4F, G & Supplementary Fig. 4E). Moreover, we perform immunofluorescence assays to explore the intra-cellular localization of TRIM28 and PPARG. We found both TRIM28 and PPARG are localized in the nucleus department and the fluorescence signals of TRIM28 and PPARG overlap obviously (Fig. 4H & Supplementary Fig. 4F). We further construct truncated plasmid to explore the potential interaction region between PPARG and TRIM28 (Fig. 4I, J). PPARG is truncated to N-terminal (1-204 aa) and C-terminal (205-505 aa). Our Co-IP results show TRIM28 mainly interacts with the N-terminal of PPARG (Fig. 4K). TRIM28 is truncated into 5 truncated-plasmid according to its specific region. Our Co-IP results show the PHD region is indispensable for TRIM28 to bind with PPARG (Fig. 4L).

### TRIM28 stabilizes PPARG to promote cholesterol metabolism in BLCA cells

**Fig. 5:**
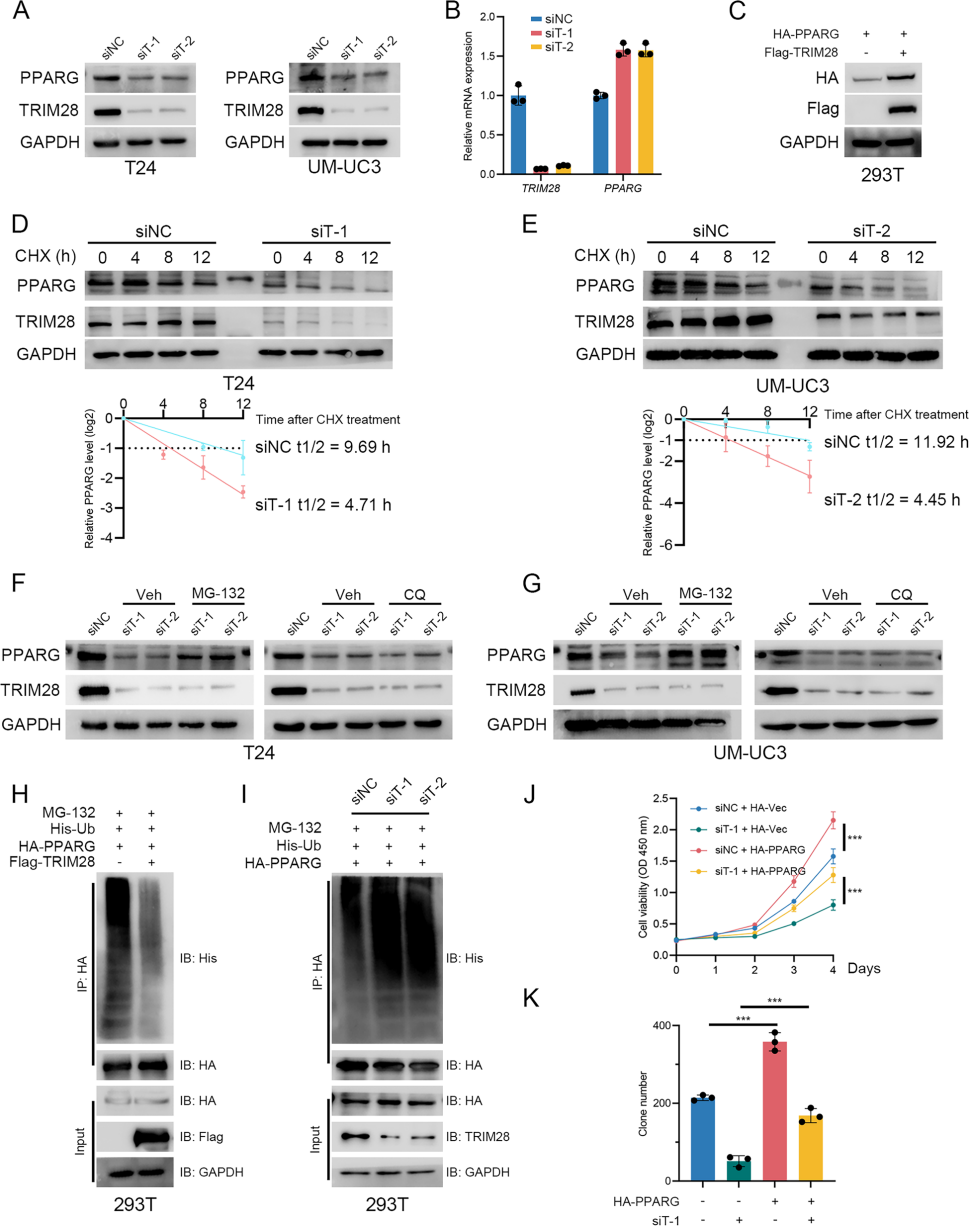
TRIM28 stabilizes PPARG. **A** WB assays detecting the protein level of PPARG after silencing TRIM28. **B** qRT-PCR assays detecting the mRNA level of *PPARG* after silencing *TRIM28*. **C** WB assays detecting the protein level of HA-PPARG after overexpressing Flag-TRIM28 in HEK-293T cells. **D** WB assays assessing the effect of TRIM28 silencing on PPARG protein stabilization in T24 cells following CHX treatment (50 µg/mL) and densitometric analysis. **E** WB assays assessing the effect of TRIM28 silencing on PPARG protein stabilization in UM-UC3 cells following CHX treatment (50 µg/mL) and densitometric analysis. **F** WB assays assessing PPARG protein levels in TRIM28-silenced T24 cells with MG132 (10 µM, 8 h) or CQ (20 µM, 8 h). **G** WB assays assessing PPARG protein levels in TRIM28-silenced UM-UC3 cells with MG132 (10 µM, 8 h) or CQ (20 µM, 8 h). **H** Ubiquitination assay assessing the exogenous ubiquitination levels of PPARG following TRIM28 overexpression in HEK-293T cells. **I** Ubiquitination assay assessing the exogenous ubiquitination levels of PPARG in TRIM28-silenced in HEK-293T cells. **J**, **K** CCK-8 assays and clone formation assays for T24 cells with PPARG overexpression and TRIM28 silencing. ^*^P < 0.05, ^**^P < 0.01, ^***^P < 0.001.

After confirming the interaction between TRIM28 and PPARG, we further explore whether TRIM28 can regulate the expression of PPARG. Our western blot results show that silencing TRIM28 effectively reduces the protein level of PPARG while does not reduce the mRNA level of *PPARG* (Fig. 5A, B). Overexpressing TRIM28 increases the protein level of PPARG (Fig. 5C & Supplementary Fig. 5A). We also add CHX to inhibit the synthesis of nascent proteins and we found silencing TRIM28 significantly shortens the half-life of PPARG (Fig. 5D, E), while overexpressing TRIM28 strengthens the stability of PPARG (Supplementary Fig. 5B). These results demonstrate that TRIM28 regulates the protein level of PPARG, especially the half-life of PPARG. The degradation of intracellular proteins is mainly accomplished through the ubiquitin-proteasome pathway or the autophagy-lysosome pathway, therefore we next explore how TRIM28 inhibits the degradation of PPARG. We find adding MG-132, the inhibitor of 26s proteasome, could effectively restores the reduced expression of PPARG after silencing TRIM28 (Fig. 5F, G), while adding CQ, the inhibitor of autophagy, fails to restore the reduced expression of PPARG (Fig. 5F, G). Moreover, ubiquitination experiment show overexpressing TRIM28 reduces the ubiquitination level of PPARG (Fig. 5H), while silencing TRIM28 elevating the ubiquitination level of PPARG (Fig. 5I). Moreover, CCK-8 and clone formation assays show overexpressing PPARG significantly restores the reduced proliferation ability of BLCA cells caused by silencing TRIM28 (Fig. 5J, K & Supplementary Fig. 5C). Overexpressing PPARG also restores the decreased cholesterol level in TRIM28-silenced BLCA cells (Supplementary Fig. 5D). Moreover, to further substantiate the PPARG-dependent role of TRIM28 in regulating intracellular cholesterol levels, we generated PPARG-knockout (sgPPARG) cells (Supplementary Fig. 5E). Notably, additional TRIM28 silencing in sgPPARG cells does not further decrease intracellular cholesterol content (Supplementary Fig. 5F). These results confirm that TRIM28-mediated regulation of cholesterol levels in bladder cancer cells is dependent on PPARG. The above results indicate that the interaction between TRIM28 and PPARG enhances the stability of PPARG protein and therefore promoting BLCA progression.

### TRIM28 enhances the K97 SUMOylation of PPARG

**Fig. 6:**
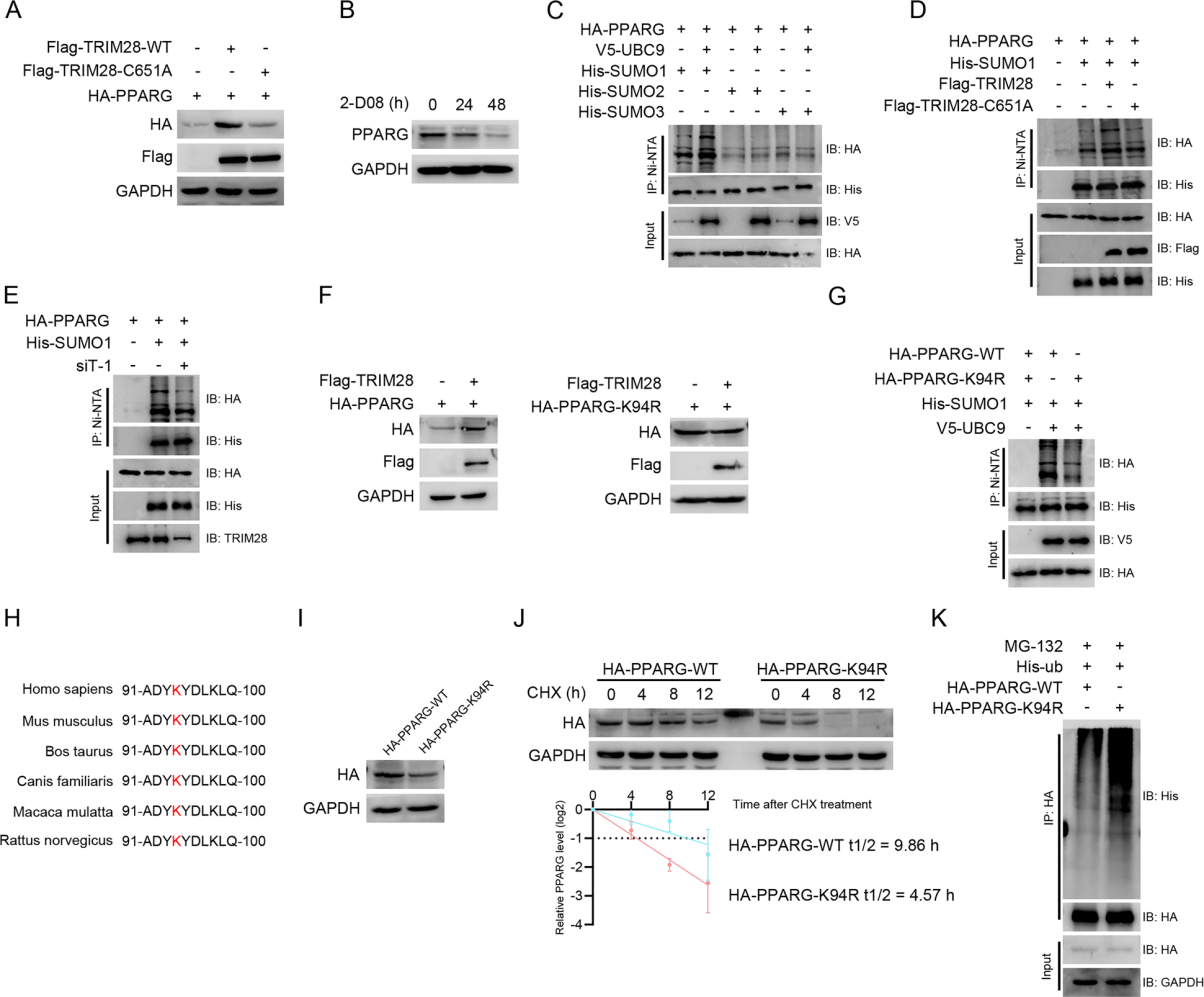
TRIM28 regulates SUMOylation of PPARG at Lysine 94. **A** Overexpression immunoblots in HEK-293T cells showing that Flag-TRIM28-WT, but not the SUMO-E3-deficient mutant C651A, increases HA-PPARG protein abundance. **B** Pharmacologic inhibition of SUMOylation with 2-D08 lowers endogenous PPARG protein level. **C** His-SUMO pull-down (Ni-NTA) demonstrating PPARG SUMOylation. **D** TRIM28 dependence of PPARG SUMOylation. **E** TRIM28 knockdown decreases PPARG SUMOylation. **F** The K94R mutant of PPARG blunts the TRIM28-mediated increase in PPARG abundance. **G** The K94R mutant of PPARG blunts the SUMOylation of PPARG. **H** Cross-species conservation of the PPARG region surrounding K94 (amino acids 91–100). **I** Expression control showing comparable baseline levels of HA-PPARG-WT and HA-PPARG-K94R. **J** Cycloheximide (CHX) chase comparing the half-life of wild-type PPARG and K94R-mutant PPARG. **K** In-vivo ubiquitination assay comparing the ubiquitination level of wild-type PPARG and K94R-mutant PPARG.

Besides the well-known function of a ubiquitination E3 ligase [23], the PHD region enables TRIM28 to function as a TRIM28 SUMO E3 ligase, thereby inhibiting the degradation of substrates [24, 25]. Considering TRIM28 elevates the stabilization of PPARG, we speculate that TRIM28 can regulate the SUMOylation of PPARG. Therefore, we mutate the cysteine at position 651 of TRIM28 to alanine to simulate the loss-function of SUMO E3 ligase. Western blot results show that TRIM28-C651A mutant fail to elevate the protein level of PPARG (Fig. 6A). Treatment of bladder cancer cells with the SUMOylation inhibitor 2-D08 markedly reduces PPARG protein expression (Fig. 6B). To characterize the type of SUMO modification on PPARG, we perform SUMO isoform-specific assays and find that PPARG is predominantly modified by SUMO1 (Fig. 6C). Overexpression of wild-type TRIM28, but not the E3 ligase-defective mutant TRIM28-C615A, significantly increase PPARG SUMOylation (Fig. 6D), whereas TRIM28 knockdown leads to a notable decrease in PPARG SUMOylation (Fig. 6E). SUMOylation typically occurs on lysine residues of substrate proteins. Using the JASSA database, we predict nine potential SUMOylation sites on PPARG and generate corresponding lysine-to-arginine (K → R) substitution mutants. Co-transfection experiments with each PPARG mutant and TRIM28 reveal that TRIM28 fails to enhance the protein expression of the K94R mutant (Fig. 6F & Supplementary Fig. 6), and the SUMOylation level of K94R PPARG is markedly lower than that of wild-type PPARG (Fig. 6G). Sequence alignment across multiple species demonstrates that Lys94 of PPARG is evolutionarily conserved (Fig. 6H). Interestingly, despite transfection with equal plasmid amounts, K94R-mutant PPARG exhibits a substantially lower expression level (Fig. 6I), a shorter protein half-life (Fig. 6J), and a higher degree of ubiquitination (Fig. 6K) compared with wild-type PPARG. Collectively, these results indicate that TRIM28 stabilizes PPARG by promoting SUMOylation at Lys94, thereby reducing its ubiquitin-mediated degradation.

### TRIM28-mediated SUMOylation of PPARG at Lys94 inhibits STUB1-mediated ubiquitin–proteasome degradation

**Fig. 7:**
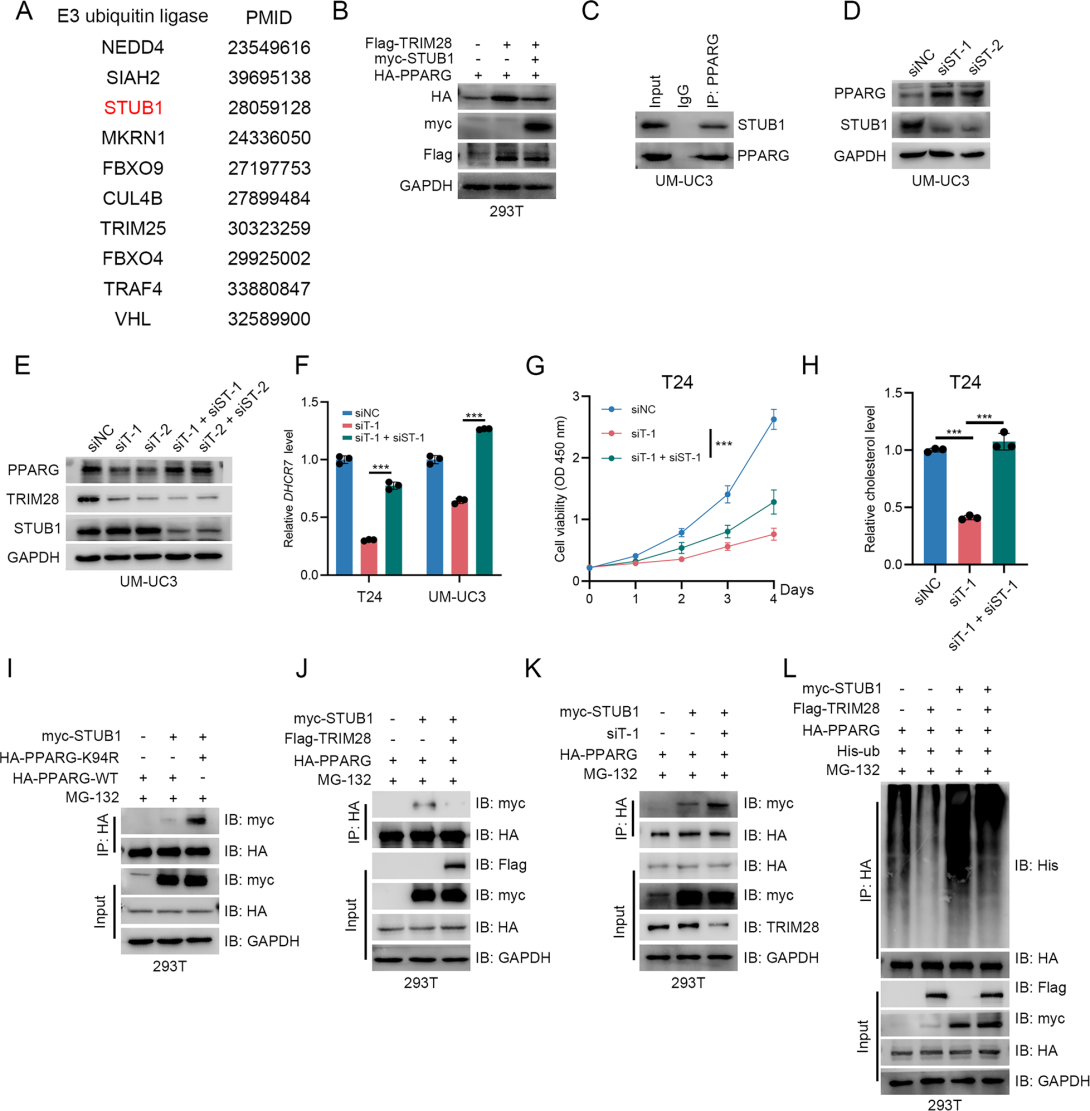
TRIM28-mediated SUMOylation of PPARG inhibits STUB1-mediated PPARG degradation. **A** Literature-derived shortlist of candidate PPARG E3 ubiquitin ligases with PubMed IDs. **B** HEK-293T overexpression immunoblot showing STUB1 counteracts TRIM28-induced increase of PPARG protein. **C** Endogenous PPARG-STUB1 interaction in UM-UC3 cells detected by Co-IP assay. **D** Detection of PPARG protein level after silencing STUB1 in UM-UC3 cells. **E** Detection of PPARG protein level after co-silencing STUB1 and TRIM28 in UM-UC3 cells. **F** Detection of *DHCR7* mRNA level after co-silencing STUB1 and TRIM28 in T24 and UM-UC3 cells. **G** CCK-8 cell-viability curves for T24 cells after co-silencing STUB1 and TRIM28. **H** Detection of endogenous cholesterol level after co-silencing STUB1 and TRIM28 in T24 cells. **I** Co-IP in HEK-293T cells comparing STUB1 binding to HA-PPARG-WT versus HA-PPARG-K94R. **J** Co-IP in HEK-293T cells showing that TRIM28 co-expression weakens the association between STUB1 and PPARG. **K** Co-IP in HEK-293T cells showing that silencing TRIM28 enhances STUB1 association with PPARG. **L** In-vivo ubiquitination assay detecting the ubiquitination level of PPARG with co-transfecting TRIM28 and STUB1 in HEK-293T cells. ^*^P < 0.05, ^**^P < 0.01, ^***^P < 0.001.

The degradation of proteins via the ubiquitin-proteasome pathway requires the recognition of substrate proteins by E3 ubiquitin ligases, and we obtain a list of ten E3 ubiquitin ligase that facilitate the degradation of PPARG according to previous reports (Fig. 7A) [26–35]. Our results show that only co-transfecting with STUB1, the up-regulated PPARG level by TRIM28 is off-set (Fig. 7B & Supplementary Fig. 7A–I). Moreover, SUTB1 was also reported a negative regulator of BLCA progression [36]. Therefore, we attempt to investigate whether TRIM28 mediated SUMOylation at the K94 site of PPARG inhibits STUB1’s ubiquitination of PPARG, thereby reducing its degradation. Our endogeneous Co-IP assays reveal the interaction of PPARG and STUB1 in BLCA cells (Fig. 7C), and the western blot results show silencing STUB1 can elevate the protein level of PPARG (Fig. 7D & Supplementary Fig. 7J). Meanwhile, further silencing STUB1 restore the reduced level of PPARG by silencing TRIM28 (Fig. 7E & Supplementary Fig. 7K) and silencing STUB1 can elevate the mRNA level of *DHCR7* (Fig. 7F). Silencing STUB1 also re-elevates the proliferation ability and intracellular cholesterol level in TRIM28-silenced BLCA cells (Fig. 7G, H & Supplementary Fig. 7L). Moreover, STUB1 is more inclined to interact with K94R-mutated PPARG (Fig. 7I), and K94R-mutated PPARG is much more ubiquitinated by STUB1 compared with wild type PPARG (Supplementary Fig. 7M). We further explore the potential competition of TRIM28 and STUB1 in binding PPARG. Co-IP assays show overexpressing TRIM28 reduces the interaction between STUB1 and PPARG (Fig. 7J), while silencing TRIM28 promotes the interaction between STUB1 and PPARG (Fig. 7K). Ubiquitination assays show overexpressing TRIM28 reduces the increased ubiquitination level of PPARG by STUB1 (Fig. 7L), while silencing TRIM28 further elevates the ubiquitination level of PPARG by STUB1 (Supplementary Fig. 7N). Collectively, the above results show the SUMOylation of K94 on PPARG mediated by TRIM28 inhibits the ubiquitination of PPARG by STUB1.

## Discussion

Accumulating evidence suggests that members of the TRIM (Tripartite Motif) family play crucial roles in intracellular signaling, transcriptional regulation, innate immunity, autophagy, and tumorigenesis [37–39]. Among them, TRIM28 (Tripartite Motif Containing 28), also known as TIF1β or KAP1 (KRAB-associated protein 1), is a highly conserved protein of 835 amino acids. TRIM28 exerts multifaceted biological functions, including transcriptional repression or activation and post-translational modification of downstream effectors. Structurally, it possesses the characteristic TRIM domain architecture comprising an N-terminal RING finger, two B-box motifs, and a coiled-coil region, followed by a C-terminal PHD-Bromo domain, which collectively dictate its E3 ligase activity and scaffolding potential.

Functionally, TRIM28 acts as both a transcriptional regulator and an E3 ubiquitin or SUMO ligase, depending on cellular context. It can function as a transcriptional activator by directly binding to mutant *hTERT* promoter alleles, promoting telomerase expression [40]. As a classical E3 ubiquitin ligase, TRIM28 interacts with p27 to induce its ubiquitination and proteasomal degradation, facilitating hepatocellular carcinoma cell-cycle progression [41]. In colorectal cancer, PRMT5-mediated symmetric dimethylation of ALKBH5 at Arg316 enhances TRIM28-dependent ubiquitination and degradation of ALKBH5, leading to CD276 upregulation and immune evasion [23]. Additionally, through its C-terminal PHD-Bromo domain, TRIM28 exhibits E3 SUMO ligase activity, mediating SUMOylation of PD-L1 and protecting it from proteasomal degradation, which in turn promotes tumor immune escape [25]. TRIM28 has also been shown to compete with SENP3 in regulating SUMOylation of ACSL4 at Lys352, thereby modulating its autophagic turnover [24].

Our previous work demonstrated that TRIM28 stabilizes the oncogenic transcription factor MYC by suppressing its ubiquitin-mediated degradation [15]; however, whether this process relies on the SUMO E3 ligase function of TRIM28 remained undetermined. In this study, we comprehensively analyzed TRIM family gene expression profiles in BLCA and identified TRIM28 as a key oncogenic member with distinct prognostic significance. Functional assays confirmed that TRIM28 is indispensable for BLCA cell proliferation. Transcriptomic analyses of TRIM28-depleted cells, integrated with public datasets, revealed that TRIM28 regulates cholesterol metabolism in BLCA. Proteomic interaction studies further uncovered that TRIM28 physically associates with and enhances SUMOylation of PPARG at Lys94, thereby preventing STUB1-mediated ubiquitination and proteasomal degradation. This stabilization of PPARG results in increased expression of cholesterol biosynthetic genes such as *DHCR7*, promoting lipid anabolism and malignant progression.

PPARG (Peroxisome proliferator-activated receptor gamma) is a ligand-activated nuclear receptor that serves as a master regulator of lipid and glucose metabolism, adipocyte differentiation, and energy homeostasis. Extensive studies have revealed the relationship between PPARG and various tumors, especially in BLCA [16, 22]. SUMOylation represents a critical regulatory mechanism modulating PPARG transcriptional activity and stability. Previous studies have identified Lys107 as a primary SUMOylation site catalyzed by UBC9 in cooperation with E3 ligases such as PIAS1 or PIASxα, where SUMOylation suppresses transcriptional activity, while the K107R mutation enhances transactivation and adipogenesis [42]. Phosphorylation at Ser112 further promotes Lys107 SUMOylation, establishing a dual inhibitory mechanism [43]. Conversely, de-SUMOylation by SENP2 increases phospholipid synthesis and cardiolipin accumulation in T cells [44].

Distinct from these classical regulatory events, our findings identify Lys94 as a novel SUMOylation site within PPARG. SUMO modification at this residue markedly reduces the binding affinity of PPARG to the E3 ubiquitin ligase STUB1, thereby limiting its ubiquitin–proteasome degradation. This post-translational modification leads to increased PPARG stability and activity, consequently elevating downstream cholesterol biosynthetic gene expression and driving metabolic reprogramming in BLCA cells.

Traditionally, the canonical SUMOylation consensus motif is defined as ΨKxE, where Ψ represents a hydrophobic amino acid and x denotes any residue. Intriguingly, the Lys94-adjacent sequence in PPARG (YKYD, residues 93-96) deviates from this motif. This noncanonical configuration implies that specialized SUMO E3 ligases, such as PIAS1 or TRIM28, can facilitate SUMO conjugation beyond classical sequence constraints. Indeed, PIAS1 has been shown to mediate SUMOylation at noncanonical motifs-for example, promoting SUMO modification of vimentin at K439 (SKRT) and K445 (IKTV), thereby enhancing cytoskeletal dynamics and cell migration [45]. We propose that while UBC9 alone can target canonical ΨKxE motifs, E3 ligases such as PIAS1 and TRIM28 enhance catalytic efficiency and broaden substrate specificity to include atypical motifs. The YKYD sequence may function as a quasi-consensus motif, where tyrosine provides partial hydrophobicity analogous to tryptophan or leucine, and aspartic acid confers an acidic charge similar to glutamate, enabling SUMO conjugation.

**Fig. 8:**
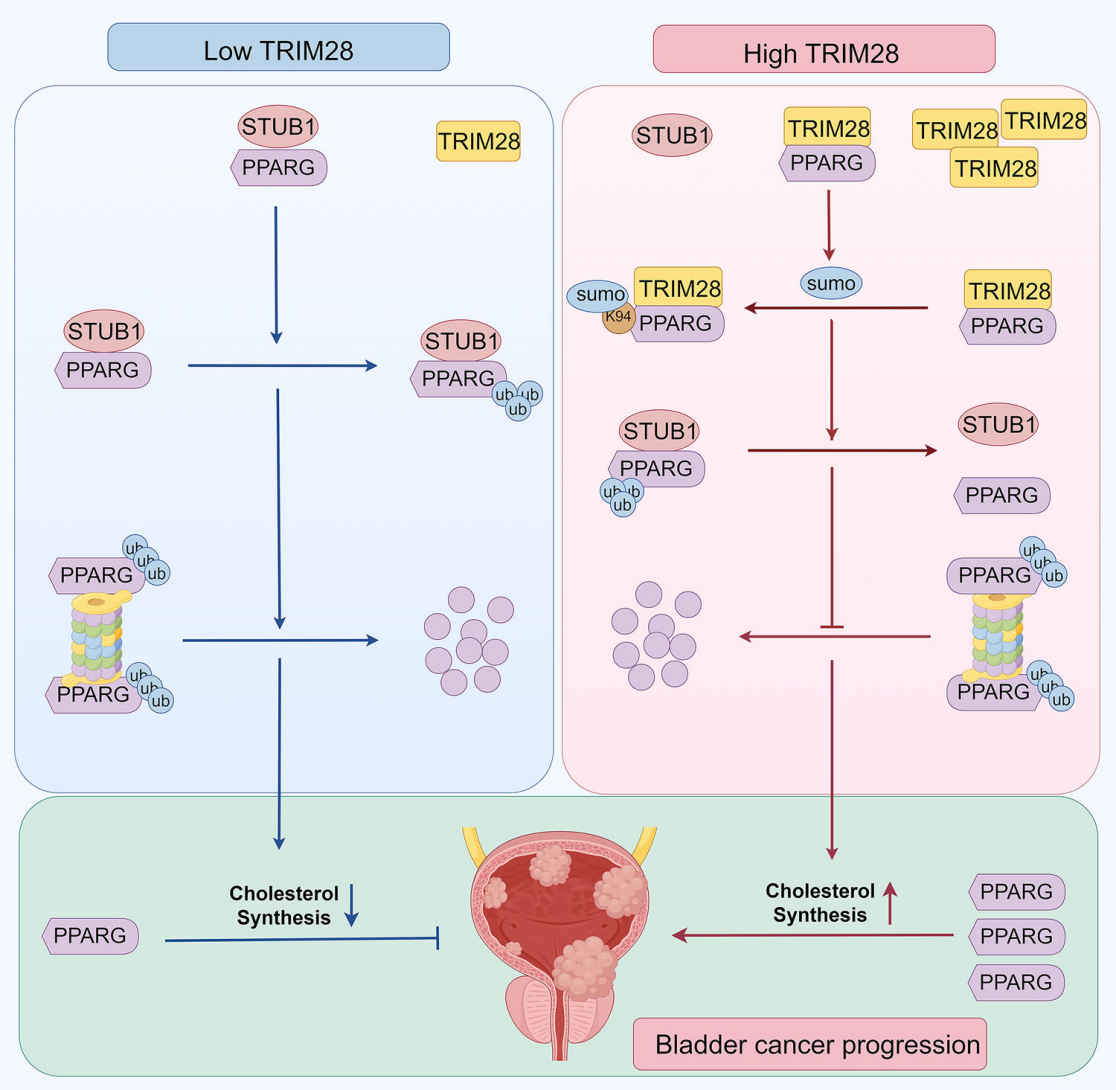
TRIM28-mediated SUMOylation of PPARG enhances cholesterol synthesis and BLCA progression. At low TRIM28 levels, STUB1 binds and ubiquitinates PPARG, promoting its proteasomal degradation and reducing cholesterol synthesis. When TRIM28 is highly expressed, it acts as a SUMO E3 ligase that catalyzes PPARG SUMOylation at Lys94, preventing STUB1 binding and stabilizing PPARG protein. The accumulation of PPARG upregulates cholesterol biosynthetic genes, thereby enhancing cholesterol metabolism and driving BLCA progression.

Collectively, our findings uncover a previously unrecognized mechanism by which TRIM28 functions as a SUMO E3 ligase to stabilize PPARG via SUMOylation at Lys94. This modification confers metabolic and oncogenic advantages to BLCA cells by enhancing PPARG-driven cholesterol biosynthesis (Fig. 8). These results not only extend the understanding of the oncogenic function of TRIM28 but also provide new insights into the noncanonical SUMOylation of PPARG, highlighting a potential therapeutic axis for metabolic intervention in BLCA.

## Supplementary information

Supplementary Figures and Tables

Uncropped data

## Data Availability

Data from the GSE13507, GSE32894 and TCGA cohorts are publicly available and are used in this study. RNA-seq data is uploaded to GEO database, and the GEO accession number is GSE318442. Proteins that interact with Flag-TRIM28 is provided in the data repository as supplementary information. Code is available from the corresponding author upon reasonable request.
