# Supplementary material for: TRIM28 orchestrates SUMO-ubiquitin crosstalk to stabilize PPARG and drive bladder cancer progression
Source: Cell Death Dis. 2026 Apr 13;17(1):475. doi: 10.1038/s41419-026-08745-7 (PMC13183881; doi:10.1038/s41419-026-08745-7)
Supplement: Supplementary file 1 — Supplementary Figures and Tables [file 41419_2026_8745_MOESM1_ESM.pdf]

Supplementary Figure legends

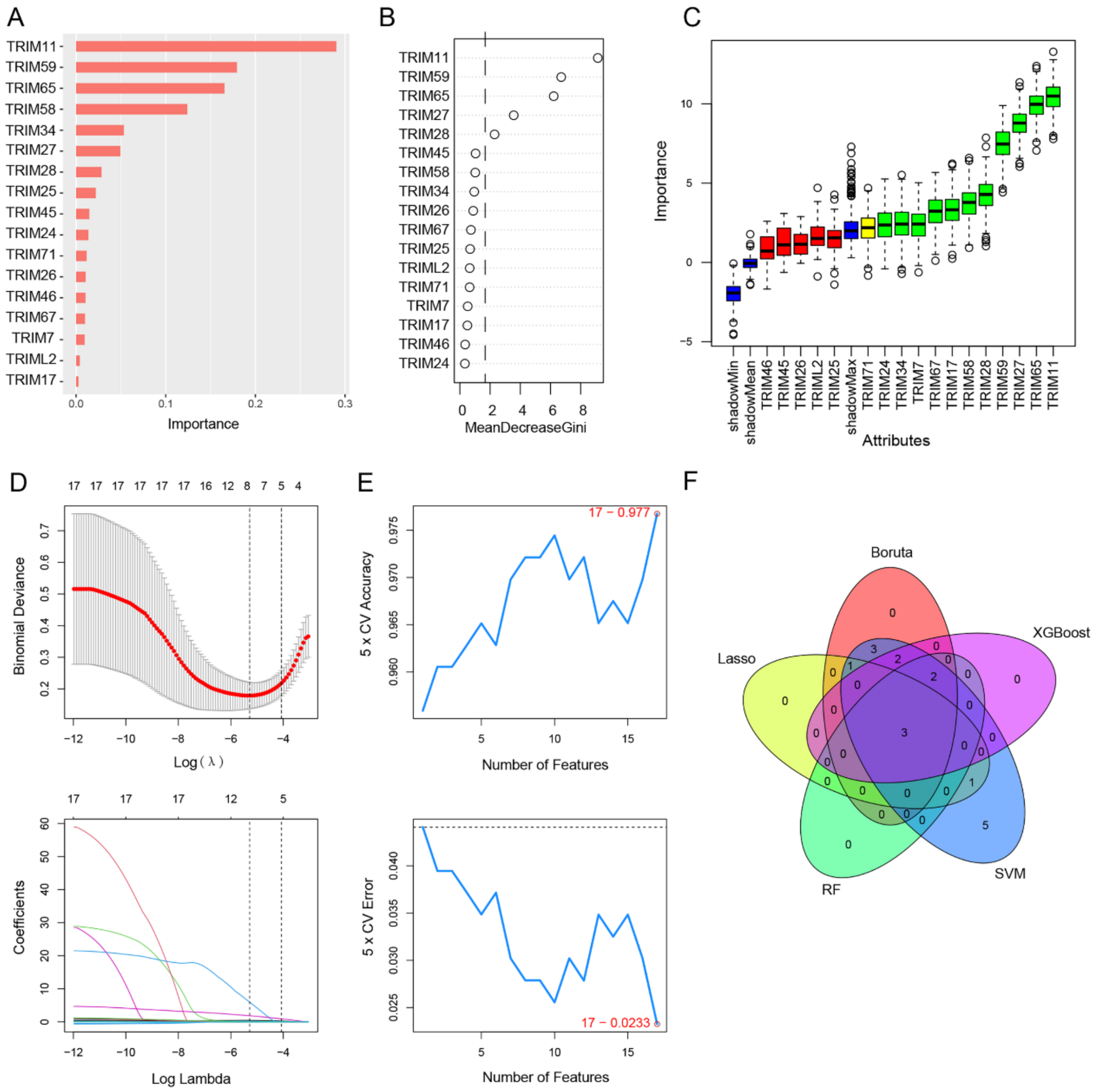

Supplementary Figure 1. Details and intersections of multi-algorithm feature selection. **(A)** Candidate variables ranked by feature importance in the XGBoost model. **(B)** Feature importance ranking and selected variables from the Random Forest model. **(C)** Feature genes identified by iterative evaluation using the Boruta algorithm. **(D)** Candidate genes selected by LASSO regression with 10-fold cross-validation. **(E)** Feature genes selected by the SVM-RFE model based on accuracy and cross-validation error curves. **(F)** Venn diagram showing the intersection of genes commonly identified across the five machine-learning algorithms.

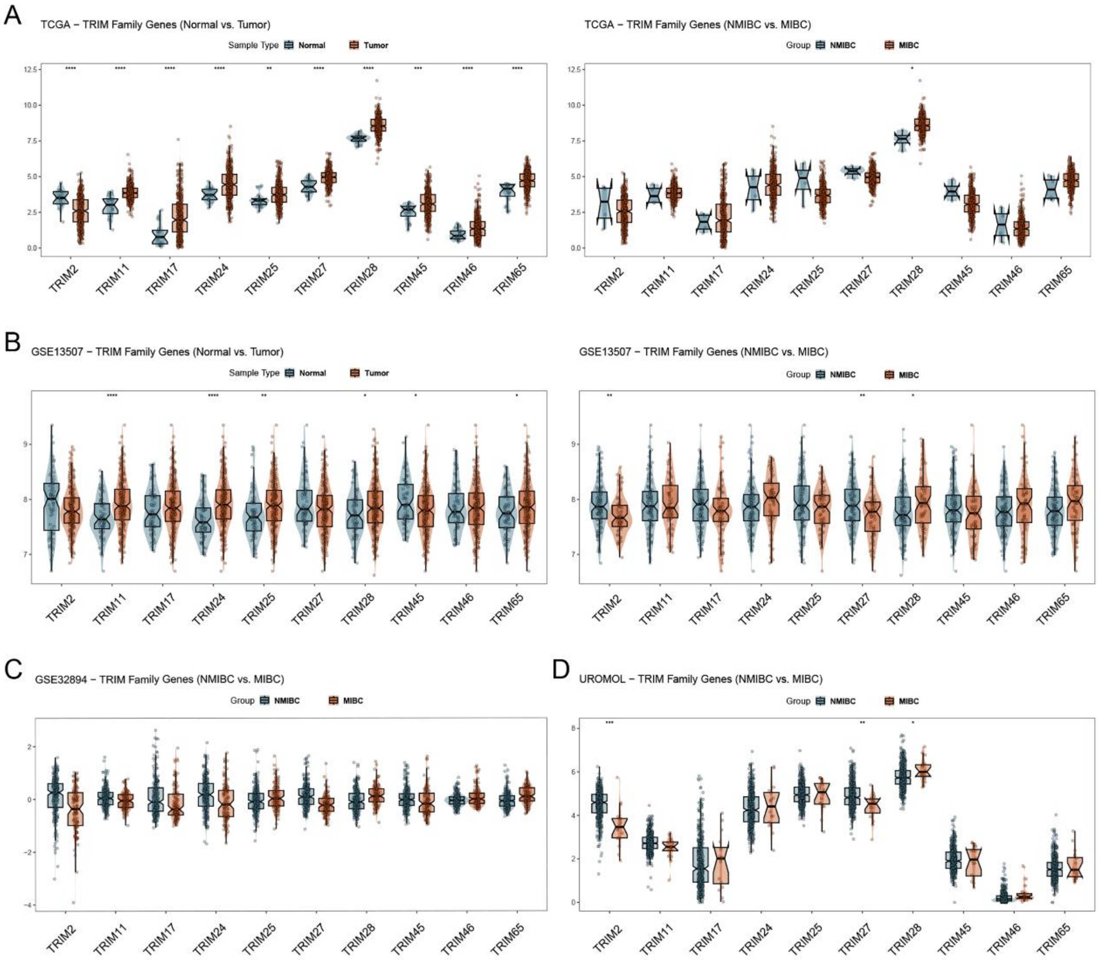

Supplementary Figure 2. Details and intersections of multi-algorithm feature selection. Cross-cohort expression differences and consistency analysis of TRIM family genes. **(A)** Expression differences of TRIM family genes between normal and tumor tissues and between NMIBC and MIBC in the TCGA-BLCA cohort. **(B)** Expression differences of TRIM family genes between normal and tumor tissues and between NMIBC and MIBC in the GSE13507 dataset. **(C)** Expression differences between NMIBC and MIBC in the GSE32894 dataset. **(D)** Expression differences between NMIBC and MIBC in the UROMOL dataset. ns ≥ 0.05, $P < 0.05$, $P < 0.01$, $P < 0.001$. Boxplots display the median and interquartile range; each dot represents an individual sample, and jittering was applied for visualization only. $^{*}P < 0.05$, $^{**}P < 0.01$, $^{***}P < 0.001$.

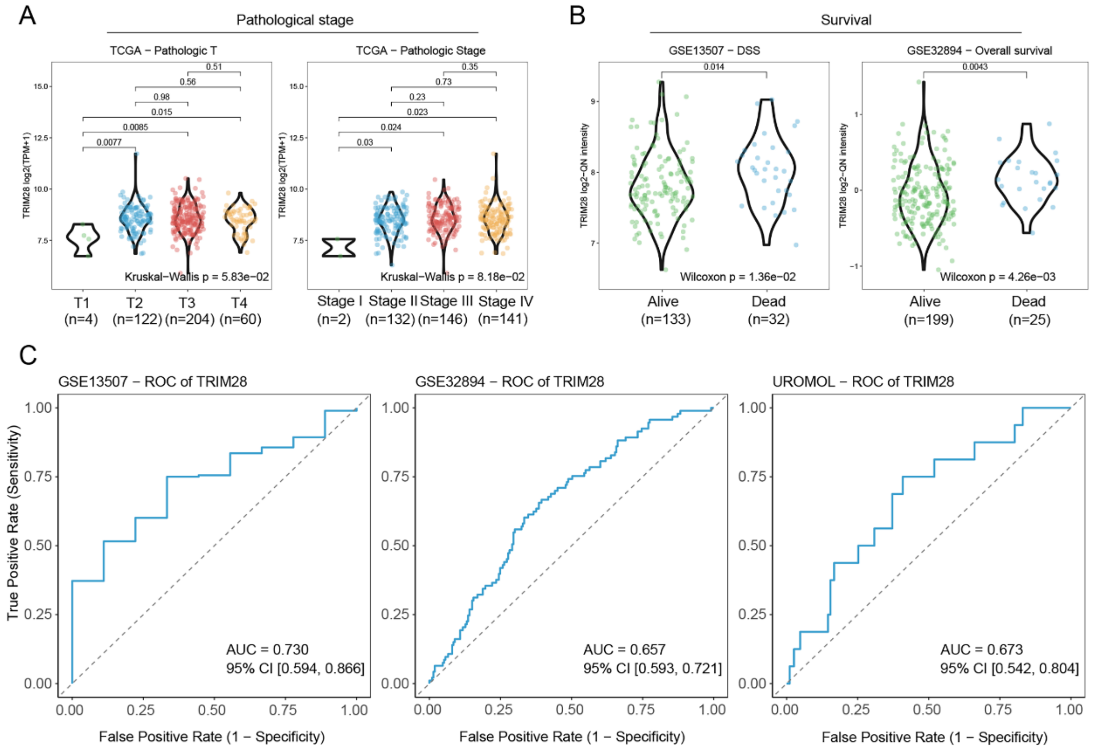

Supplementary Figure 3. Supplementary analyses of TRIM28 expression stratified by clinical features and prognosis. **(A)** Expression differences of TRIM28 across T stages and pathological stages in the TCGA-BLCA cohort. **(B)** Survival-associated expression differences of TRIM28 in GSE13507 (DSS) and GSE32894 (OS) datasets. **(C)** Time-dependent ROC curves (1-, 3-, and 5-year) for TRIM28 in the GSE32894 cohort, serving as an independent validation consistent with Figure 1I.

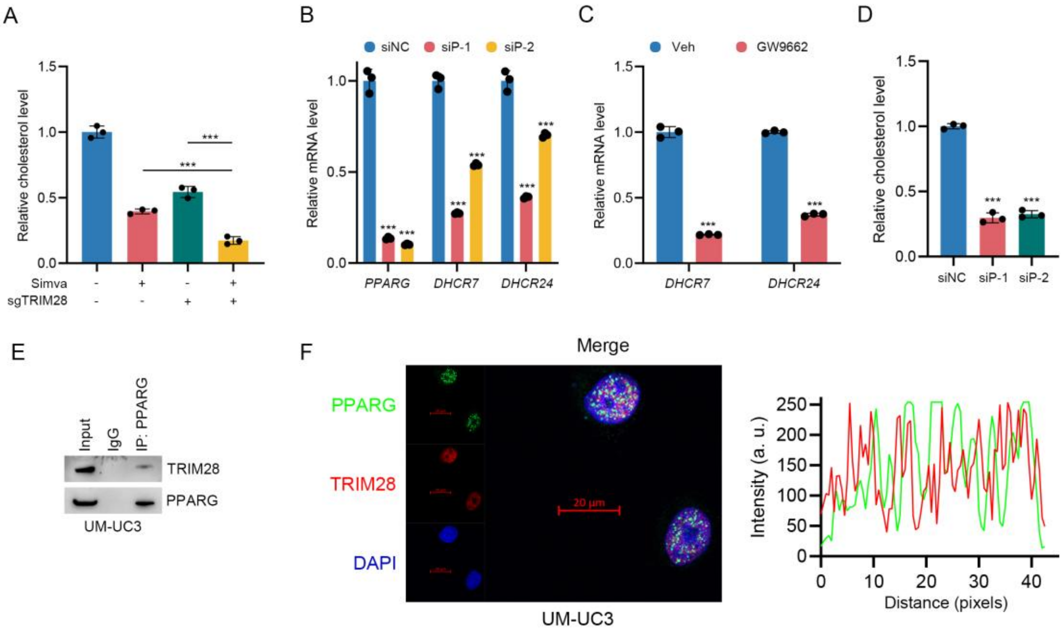

Supplementary Figure 4. TRIM28 regulates PPARG expression and interacts with PPARG in bladder cancer cells. **(A)** Relative cholesterol in T24 cells after silencing TRIM28 or treated with simvastatin (2 μM, 24 h). **(B)** qRT–PCR analysis showing the relative mRNA levels of *PPARG*, *DHCR7*, and *DHCR24* in UM-UC3 cells with PPARG silencing. **(C)** qRT–PCR analysis showing that inhibition of PPARG by GW9662 significantly reduced the expression of *DHCR7* and *DHCR24* compared with vehicle-treated controls. **(D)** Detection of endogenous cholesterol level after silencing PPARG in T24 cells. **(E)** Co-immunoprecipitation (Co-IP) assay confirming the endogenous interaction between TRIM28 and PPARG in UM-UC3 cells. **(F)** Immunofluorescence staining and line-scan intensity analysis showing the subcellular co-localization of TRIM28 (red) and PPARG (green) in UM-UC3 cells. Nuclei were counterstained with DAPI (blue). Scale bar = 20 μm. $^{*}P < 0.05$, $^{**}P < 0.01$, $^{***}P < 0.001$.

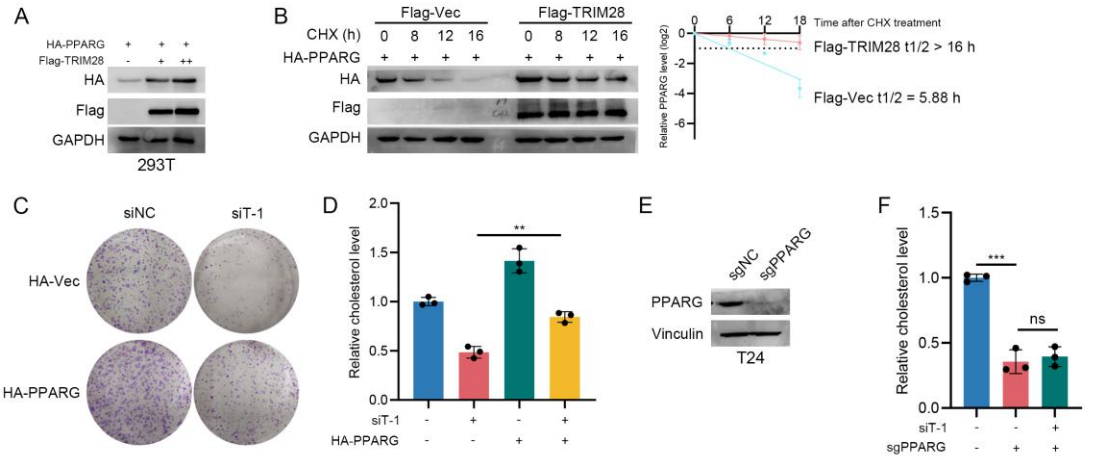

Supplementary Figure 5. TRIM28 enhances the stability and oncogenic function of PPARG. **(A)** Western blot analysis of 293T cells co-transfected with HA–PPARG and Flag–TRIM28 showing increased PPARG protein levels upon TRIM28 overexpression. **(B)** CHX chase assay demonstrating that TRIM28 overexpression prolongs the half-life of PPARG. The right panel shows quantification of PPARG protein degradation. **(C)** Colony formation assay of T24 cells treated with TRIM28 silencing and PPARG overexpresssion. **(D)** Relative cholesterol in T24 cells after silencing TRIM28 or overexpressing PPARG. **(E)** Validation of PPARG knockout efficiency in T24 cells via western blot. **(F)** Relative cholesterol in sgNC cells sgPPARG cells with or without silencing TRIM28.

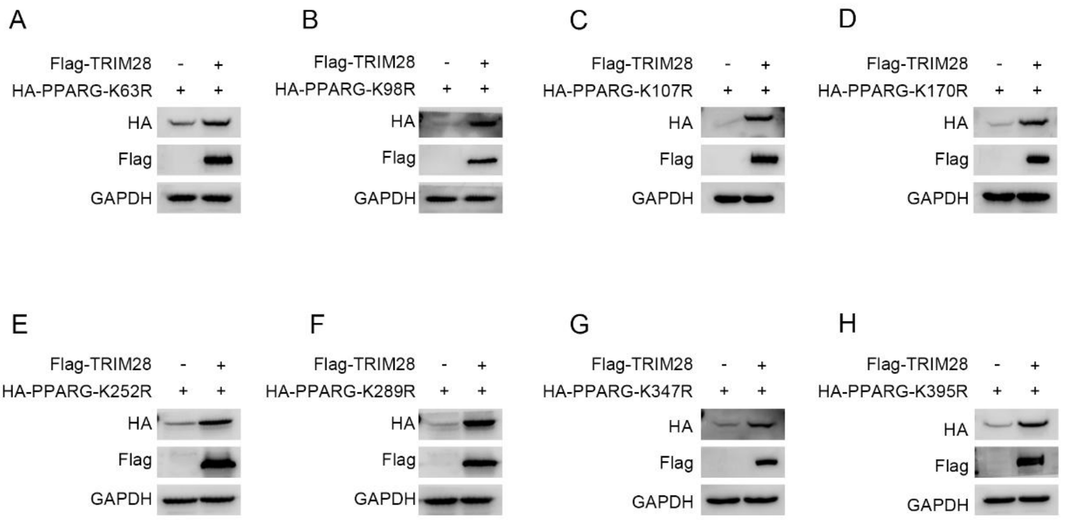

Supplementary Figure 6. Screening PPARG lysine residues for TRIM28-dependent SUMOylation. **(A–H)** HEK-293T cells were transfected with HA-tagged PPARG point mutants (K→R) at the indicated lysines and with or without Flag-TRIM28, followed by immunoblotting for HA and Flag.

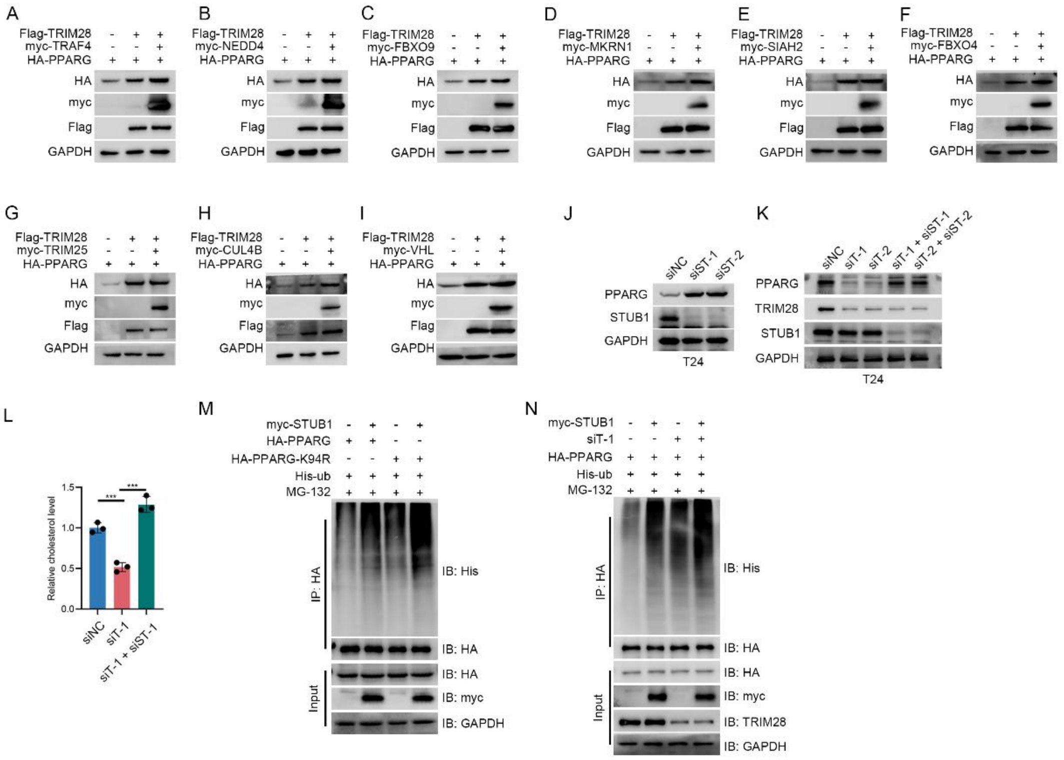

Supplementary Figure 7. TRIM28-mediated SUMOylation of PPARG inhibits STUB1-mediated PPARG degradation. **(A–I)** Specificity controls for other PPARG-related E3 ligases. HEK-293T cells were co-transfected with HA-PPARG, Flag-TRIM28, and the indicated myc-tagged E3s (TRAF4, NEDD4, FBXO9, MKRN1, SIAH2, FBXO4, TRIM25, CUL4B and VHL). Whole-cell lysates were immunoblotted with the antibodies shown. **(J)** Silencing STUB1 increases endogenous PPARG in T24 cells. **(K)** Detection of PPARG protein level after co-silencing STUB1 and TRIM28 in UM-UC3 cells. **(L)** Detection of endogenous cholesterol level after co-silencing STUB1 and TRIM28 in UM-UC3 cells. **(M)** *In-vivo* ubiquitination assay detecting the ubiquitination level of wild-type PPARG and K94R mutant PPARG with or without overexpressing STUB1. **(N)** *In-vivo* ubiquitination assay detecting the ubiquitination level of PPARG with co-transfecting TRIM28 siRNA and myc-STUB1 plasmid in HEK-293T cells.

Supplementary Table 1. Sequence of specific siRNAs and lentivirus

Target	Sense sequence (5' - 3')
siNC	UUCUCCGAACGUGUCACGUTT
siTRIM28-1 (siT-1)	GCGAUCUGGUUAUGUGCAATT
siTRIM28-2 (siT-2)	GCUCUGUUCUCUGUCCUGUCATT
siPPARG (siP)	GCUUGUACCUGCAGGAUCUTT
siSTUB1-1 (siST-1)	GGAGATGGAGAGCTATGAT
siSTUB1-2 (siST-2)	GGAGCAGGGCAATCGTCTG
sgNC	ACGGAGGCTAAGCGTCGCAA
sgTRIM28	GTTCGCATCCTGGGCGTCGG
sgPPARG	CTCCGTGGATCTCTCCGTAA

Supplementary Table 2. Primers used for qRT-PCR

Target	Forward sense (5' - 3')	Reverse sense (5' - 3')
GAPDH	CATGTACGTTGCTATCCAGGC	CTCCTTAATGTCACGCACGAT
STUB1	AGGCCAAGCACGACAAGTACAT	CTGATCTTGCCACACAGGTAGT
PPARG	ATGACAGACCTCAGACAGATTG	AATGTTGGCAGTGGCTCAG
DHCR7	AAAGCCGCCCAGCTCTATAC	TACTTGTTCACAACCCCTGC
TRIM28	TTTCATGCGTGATAGTGGCAG	GCCTCTACACAGGTCTCACAC
DHCR24	GCACAGGCATCGAGTCATCAT	GTGCATCGCACAAAGCTGC
SORT1	GTGGGTCTCCTACACCATTGATT	GAGAAAGTCCTCCAGGGAACAG
FDPS	ATTTCCGCCCTTAGTGTGGG	GACAGGGGCATCCTGTTCC
TSPO	GGTGGATCTCCTGCTGGTCA	CGACCAATACGCAGTAGTTGAGTGTG
FDFT1	CTGCACCACATCCCAGATGT	CCGAATCTTCACTGCCCCTT
ACAT2	GAGACTTACCCTAGGACGCCC	AGTTCTTGGCCACATAATTCCAC
VAPA	CCTATGCCAAAACCACAC	TGCCGATTTTCTTCTGATA
TM7SF2	AGTTGCTCTACGTGGGTGATG	CCAAACCCGTCATGTGTGAT
LSS	CTGTGCGTTCCATGGCCT	CACTGAAGTCCTGCCTGTGT

Supplementary Table 3. Antibodies used for WB, Co-IP and IHC

Target	Species	Company	Dilution
β-actin	mouse	HuaBio, EM21002	WB: 1/10000
GAPDH	rabbit	Abways, AB0036	WB: 1/10000
PPARG	rabbit	ImmunoWay, YT0819	WB: 1/1000
			Co-IP: 1 μg/mL
STUB1	rabbit	HuaBio, ET7108-65	WB: 1/1000
TRIM28	rabbit	Immunoway, YM8120	WB: 1/1000
			Co-IP: 1 μg/mL
HA-tag	mouse	Santa Cruz, sc-57592	Co-IP: 1 μg/mL
HA-tag	rabbit	HuaBio, 0906-1	WB: 1/1000
			Co-IP: 1 μg/mL
Flag-tag	rabbit	HuaBio, 0912-1	WB: 1/5000
Flag-tag	mouse	Proteintech, 66008-4-Ig	Co-IP: 1 μg/mL
myc-tag	mouse	Origene, TA150121	WB: 1/1000
His-tag	rabbit	Immunoway, YM8314	WB: 1/1000
V5-tag	rabbit	Proteintech, 14440-1-AP	WB: 1/1000
Ki67	rabbit	Proteintech, 27309-1-AP	IHC: 1/3000
Secondary	rabbit	ZSGB-Bio, ZB-2301	WB: 1/10000
antibody	mouse	ImmunoWay, RS23910	WB: 1/10000

Supplementary Table 4. Chemicals used in experiments.

Chemical	Source	Concentration
Cholesterol (Water Soluble)	MCE, HY-N0322A	20 μg/mL
Chloroquine	SparkJade, SJ-MA0048A	20 μM
MG132	Topscience, T2154	10 μM
CHX (Cycloheximide)	MCE, HY-12320	50 μg/mL
Simvastatin	Topscience, T0687	2 μM

Supplementary Table 5. Metabolism related hallmarks via GSE32894 dataset.

GeneSets	Size	NES	*P* value
WP_CHOLESTEROL_SYNTHESIS_DISORDERS	15	1.72	0.008
WP_CHOLESTEROL_METABOLISM	59	1.59	0.023
WP_CHOLESTEROL_METABOLISM_WITH_BLOCH_AND_KANDUTSCHRUSSELL_PATHWAYS	44	1.67	0.028
WP_FATTY_ACID_BIOSYNTHESIS	21	1.53	0.051
HALLMARK_CHOLESTEROL_HOMEOSTASIS	70	1.54	0.054
HALLMARK_FATTY_ACID_METABOLISM	142	0.92	0.542
HALLMARK_GLYCOLYSIS	181	0.87	0.658

Supplementary Table 6. Metabolism related hallmarks via GSE318442 dataset.

GeneSets	Size	NES	*P* value
GOBP_REGULATION_OF_CHOLESTEROL_BIOSYNTHETIC_PROCESS	29	1.57	0.000
GOBP_POSITIVE_REGULATION_OF_CHOLESTEROL_BIOSYNTHETIC_PROCESS	16	1.50	0.000
GOBP_REGULATION_OF_CHOLESTEROL_METABOLIC_PROCESS	48	1.53	0.000
WP_CHOLESTEROL_METABOLISM_WITH_BLOCH_AND_KANDUTSCHRUSSELL_PATHWAYS	46	1.52	0.000
WP_CHOLESTEROL_METABOLISM	64	1.46	0.000
WP_CHOLESTEROL_SYNTHESIS_DISORDERS	17	1.34	0.000
WP_CHOLESTEROL_BIOSYNTHESIS_PATHWAY	15	1.27	0.000
HALLMARK_CHOLESTEROL_HOMEOSTASIS	73	1.30	0.000
