# Supplementary material for: TRIM28 orchestrates SUMO-ubiquitin crosstalk to stabilize PPARG and drive bladder cancer progression
Source: Cell Death Dis. 2026 Apr 13;17(1):475. doi: 10.1038/s41419-026-08745-7 (PMC13183881; doi:10.1038/s41419-026-08745-7)

Figure 2B

T24

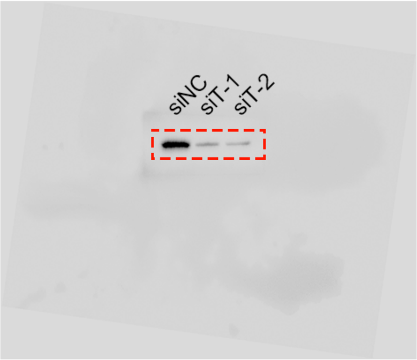

TRIM28

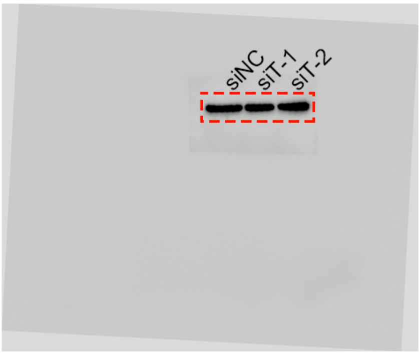

GAPDH

UM-UC3

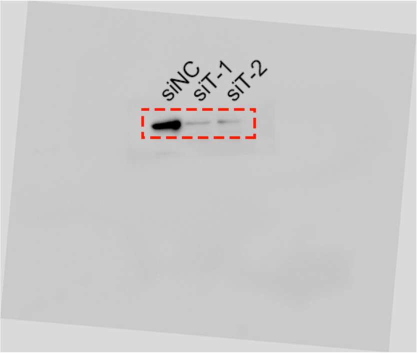

TRIM28

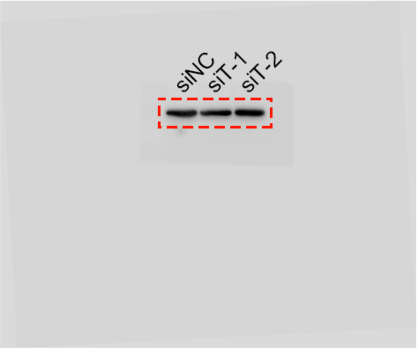

GAPDH

Figure 2J

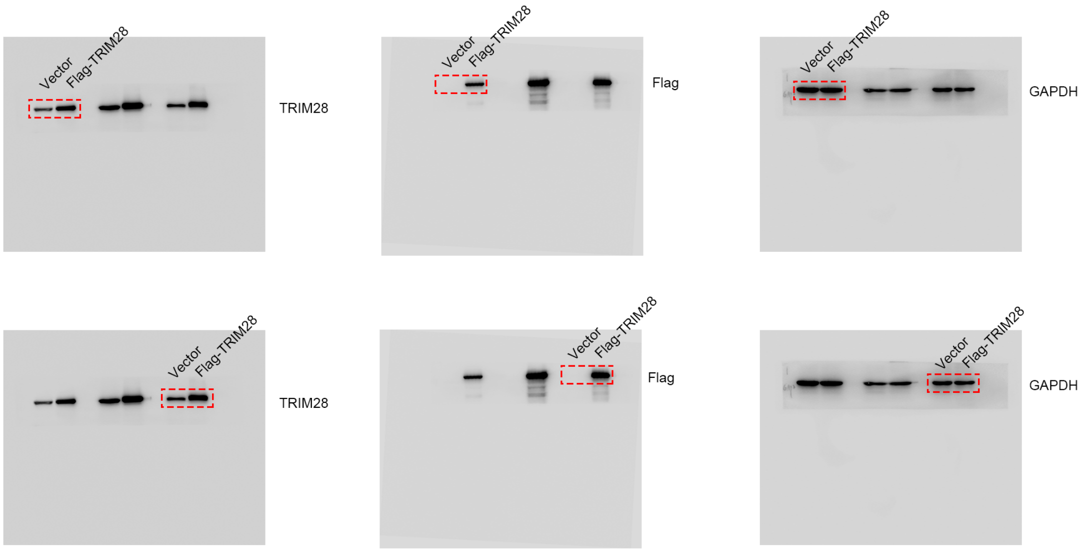
Vector
Flag-TRIM28
TRIM28
Vector
Flag-TRIM28
Flag
Vector
Flag-TRIM28
GAPDH
Vector
Flag-TRIM28
TRIM28
Vector
Flag-TRIM28
Flag
Vector
Flag-TRIM28
GAPDH

Figure 2O

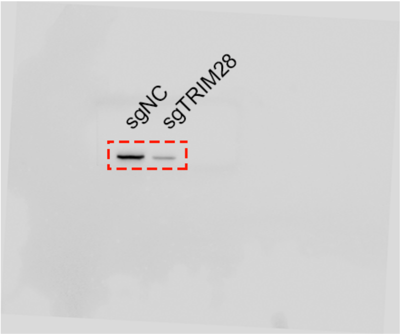

TRIM28

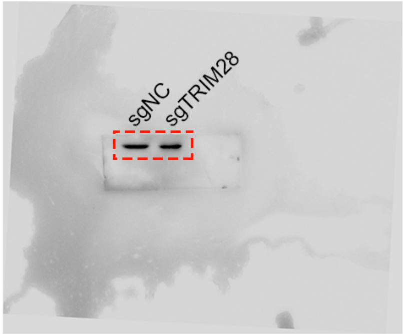

GAPDH

Figure 4D

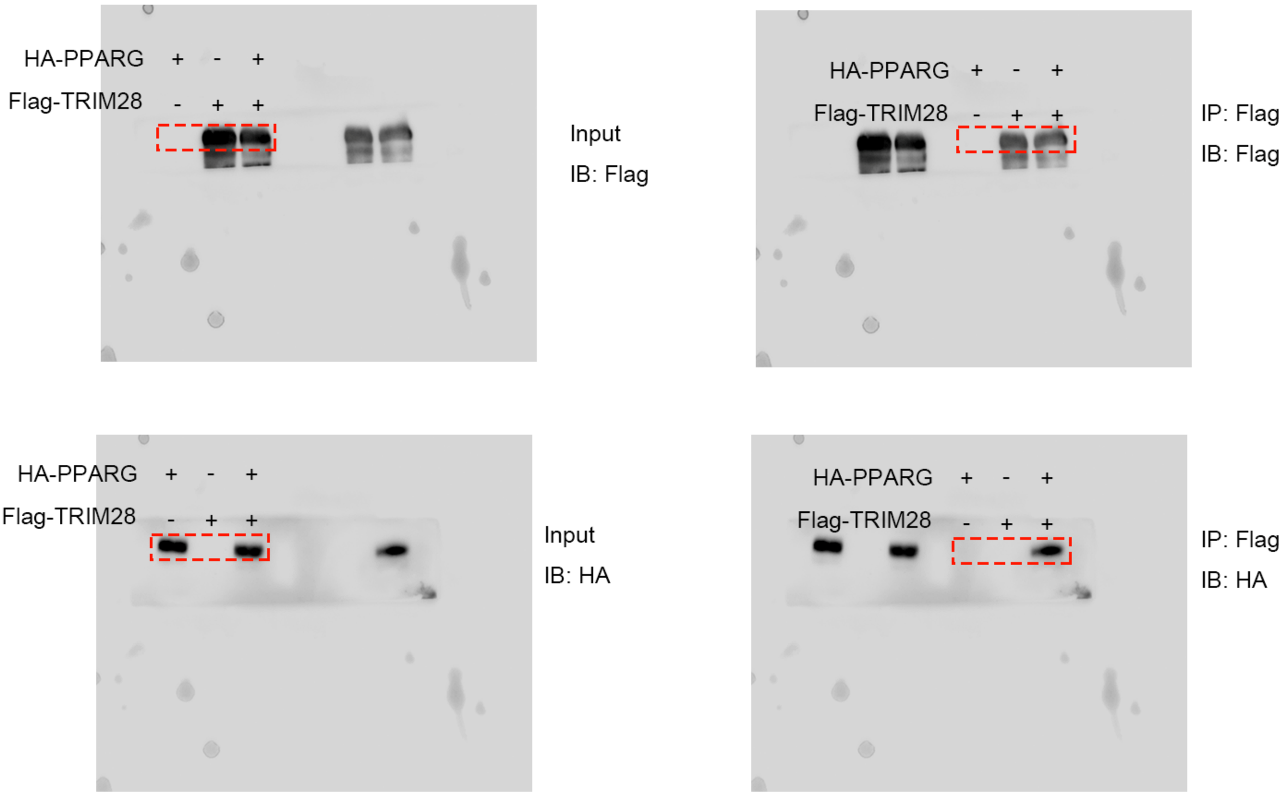

HA-PPARG + - +
Flag-TRIM28 - + +
Input
IB: Flag
HA-PPARG + - +
Flag-TRIM28 - + +
IP: Flag
IB: Flag
HA-PPARG + - +
Flag-TRIM28 - + +
Input
IB: HA
HA-PPARG + - +
Flag-TRIM28 - + +
IP: Flag
IB: HA

Figure 4E

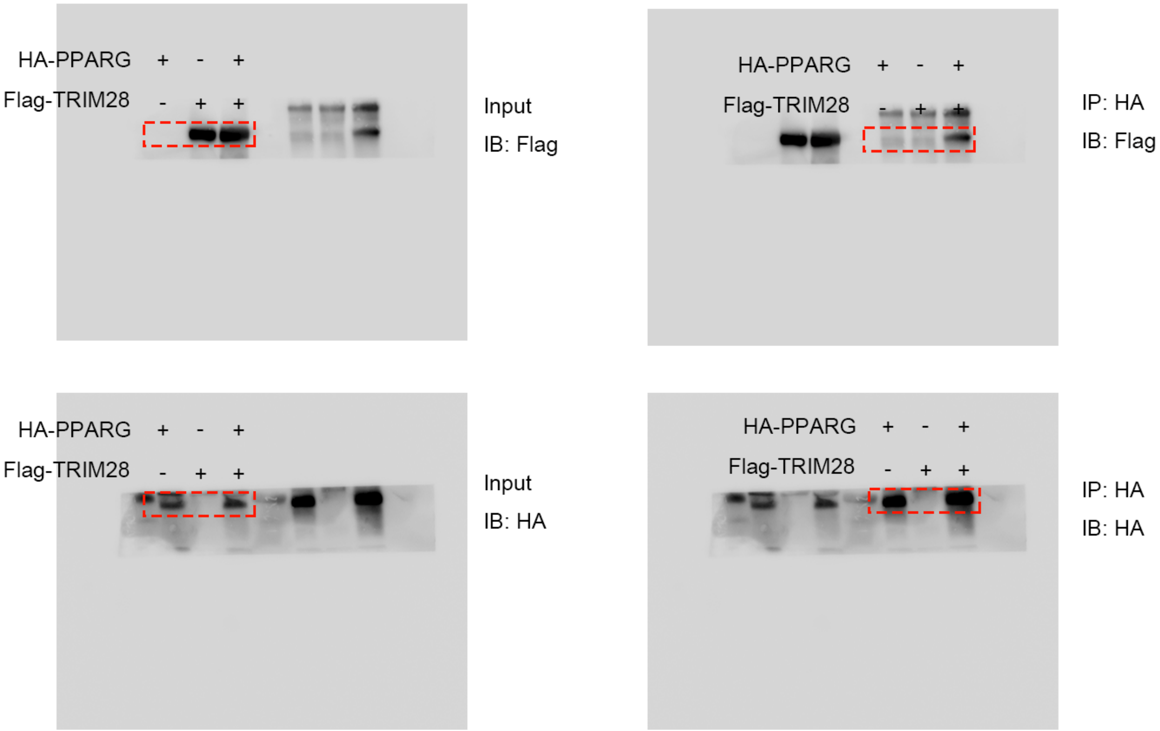
HA-PPARG + - +
Flag-TRIM28 - + +
Input
IB: Flag
HA-PPARG + - +
Flag-TRIM28 - + +
IP: HA
IB: Flag
HA-PPARG + - +
Flag-TRIM28 - + +
Input
IB: HA
HA-PPARG + - +
Flag-TRIM28 - + +
IP: HA
IB: HA

Figure 4F

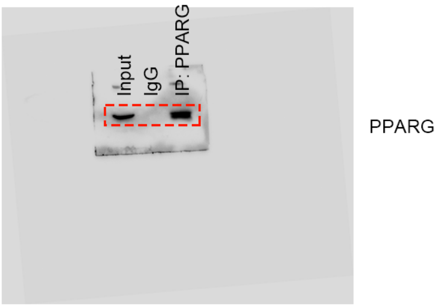

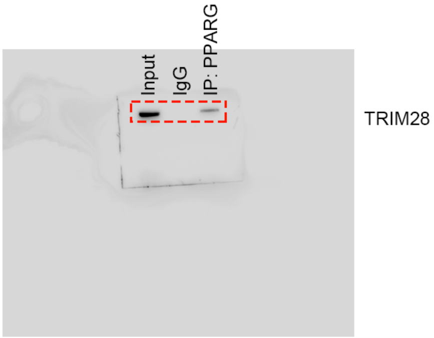

Figure 4G

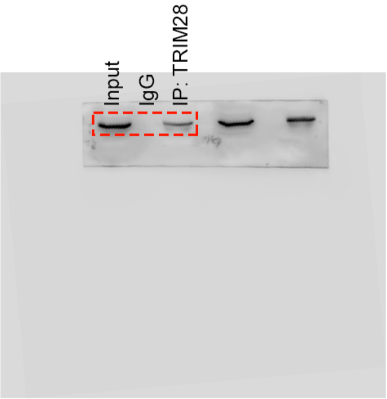

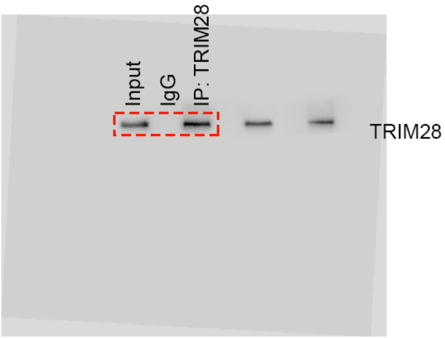

Figure 4K

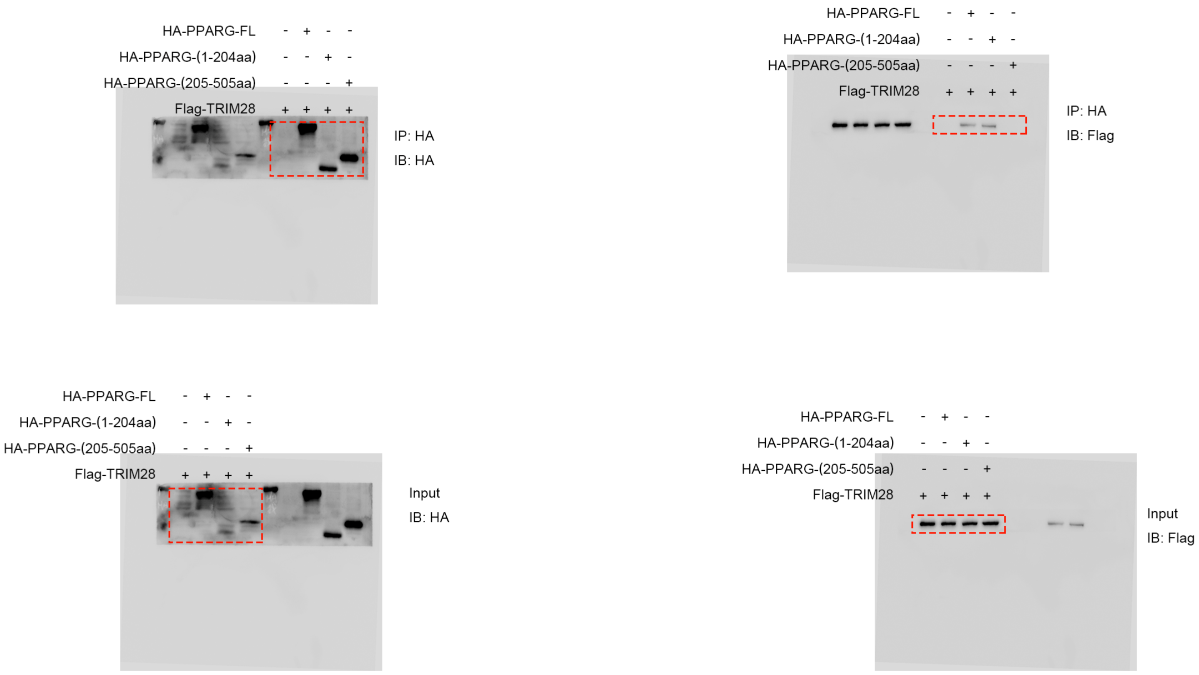

HA-PPARG-FL - + - -
HA-PPARG-(1-204aa) - - + -
HA-PPARG-(205-505aa) - - - +
Flag-TRIM28 + + + +
IP: HA
IB: HA
HA-PPARG-FL - + - -
HA-PPARG-(1-204aa) - - + -
HA-PPARG-(205-505aa) - - - +
Flag-TRIM28 + + + +
IP: HA
IB: Flag
HA-PPARG-FL - + - -
HA-PPARG-(1-204aa) - - + -
HA-PPARG-(205-505aa) - - - +
Flag-TRIM28 + + + +
Input
IB: HA
HA-PPARG-FL - + - -
HA-PPARG-(1-204aa) - - + -
HA-PPARG-(205-505aa) - - - +
Flag-TRIM28 + + + +
Input
IB: Flag

Figure 4L

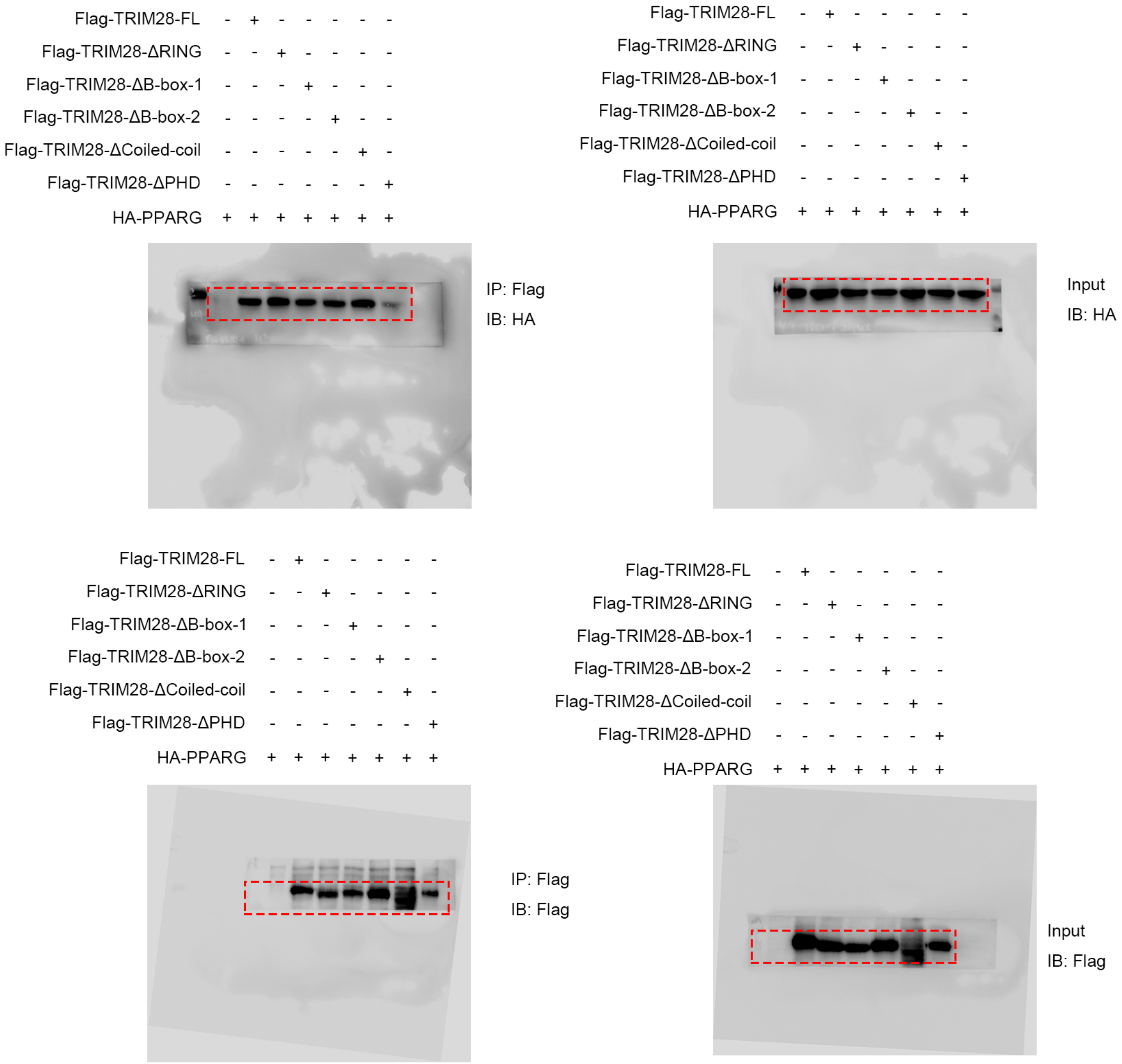
Flag-TRIM28-FL - + - - - - -
Flag-TRIM28-ΔRING - - + - - - -
Flag-TRIM28-ΔB-box-1 - - - + - - -
Flag-TRIM28-ΔB-box-2 - - - - + - -
Flag-TRIM28-ΔCoiled-coil - - - - - + -
Flag-TRIM28-ΔPHD - - - - - - +
HA-PPARG + + + + + + +
IP: Flag
IB: HA
Input
IB: HA
IP: Flag
IB: Flag
Input
IB: Flag

Figure 5A

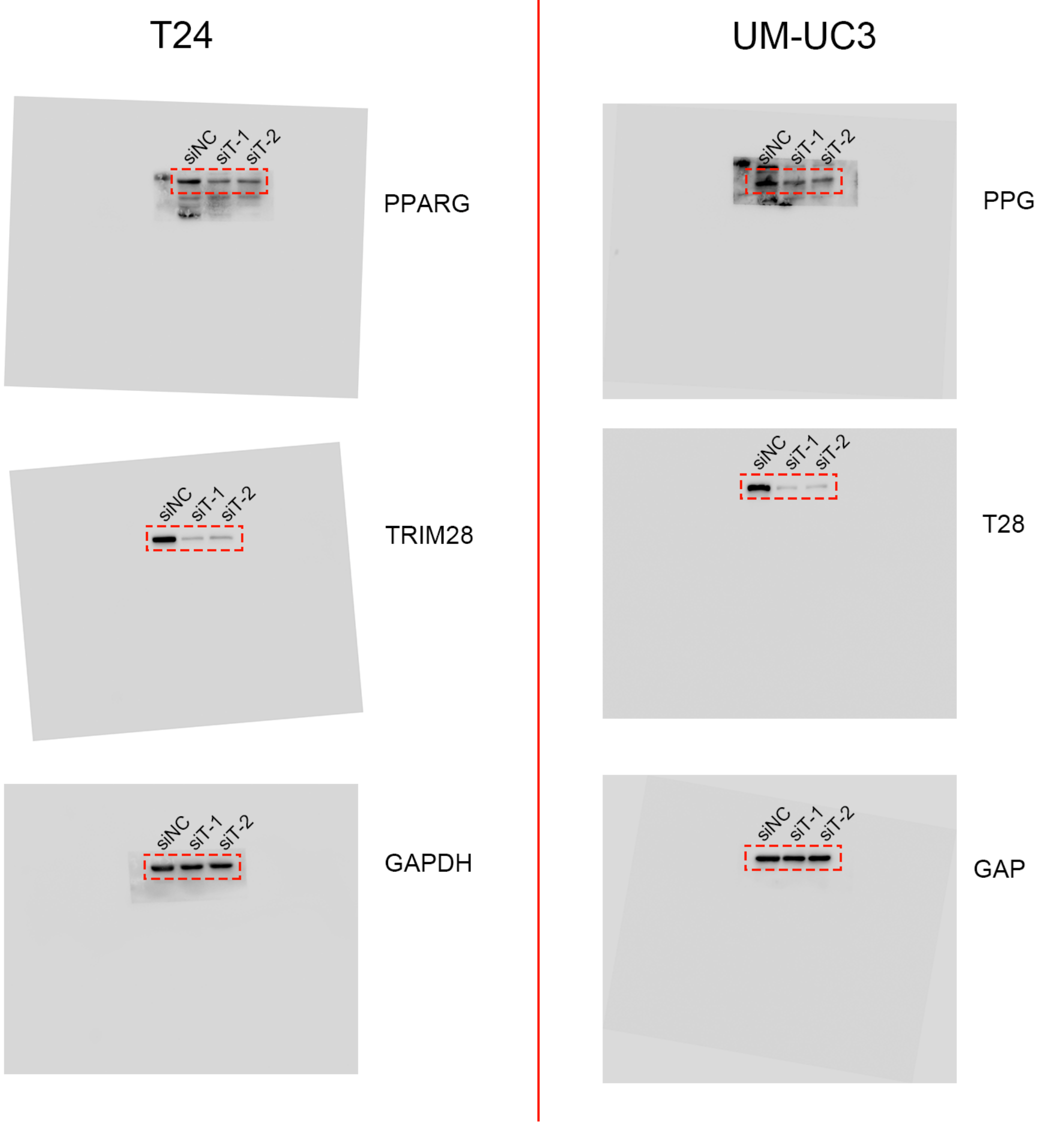

Figure 5C

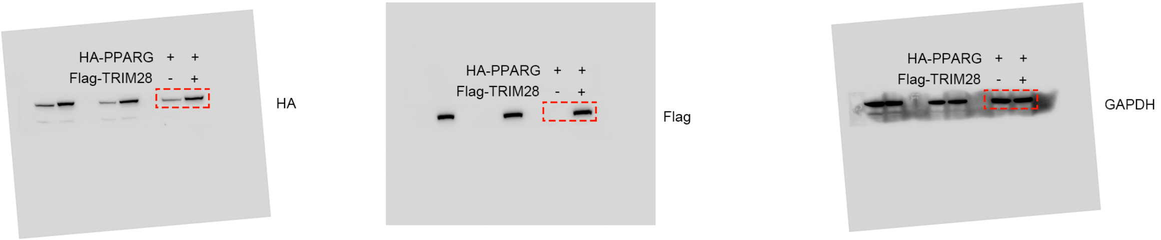
HA-PPARG + +
Flag-TRIM28 - +
HA
HA-PPARG + +
Flag-TRIM28 - +
Flag
HA-PPARG + +
Flag-TRIM28 - +
GAPDH

Figure 5D

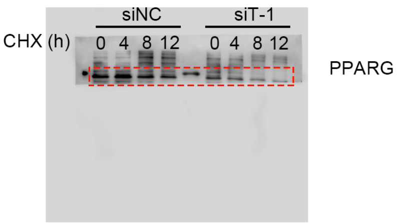
siNC
siT-1
CHX (h)
0 4 8 12 0 4 8 12
PPARG

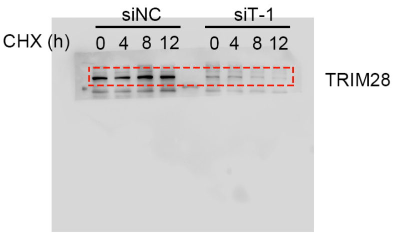
siNC
siT-1
CHX (h)
0 4 8 12 0 4 8 12
TRIM28

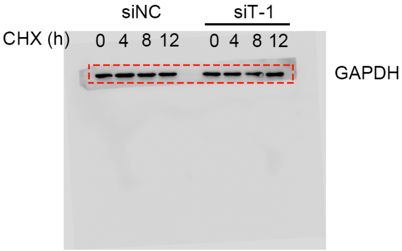
siNC
siT-1
CHX (h)
0 4 8 12 0 4 8 12
GAPDH

Figure 5E

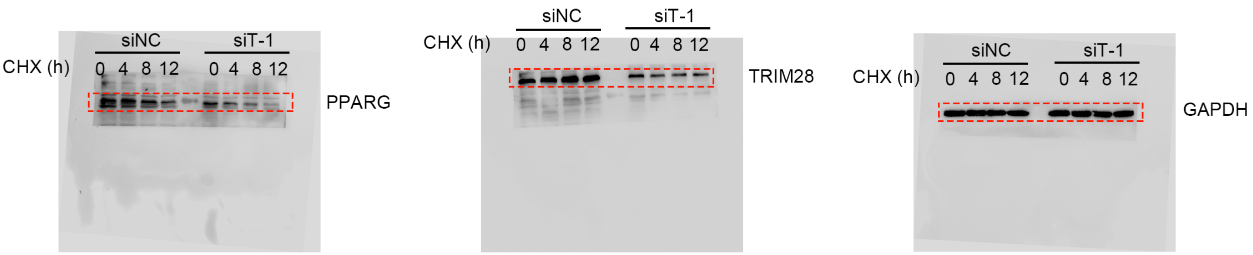

siNC
siT-1
CHX (h)
0 4 8 12 0 4 8 12
PPARG
siNC
siT-1
CHX (h)
0 4 8 12 0 4 8 12
TRIM28
siNC
siT-1
CHX (h)
0 4 8 12 0 4 8 12
GAPDH

Figure 5F

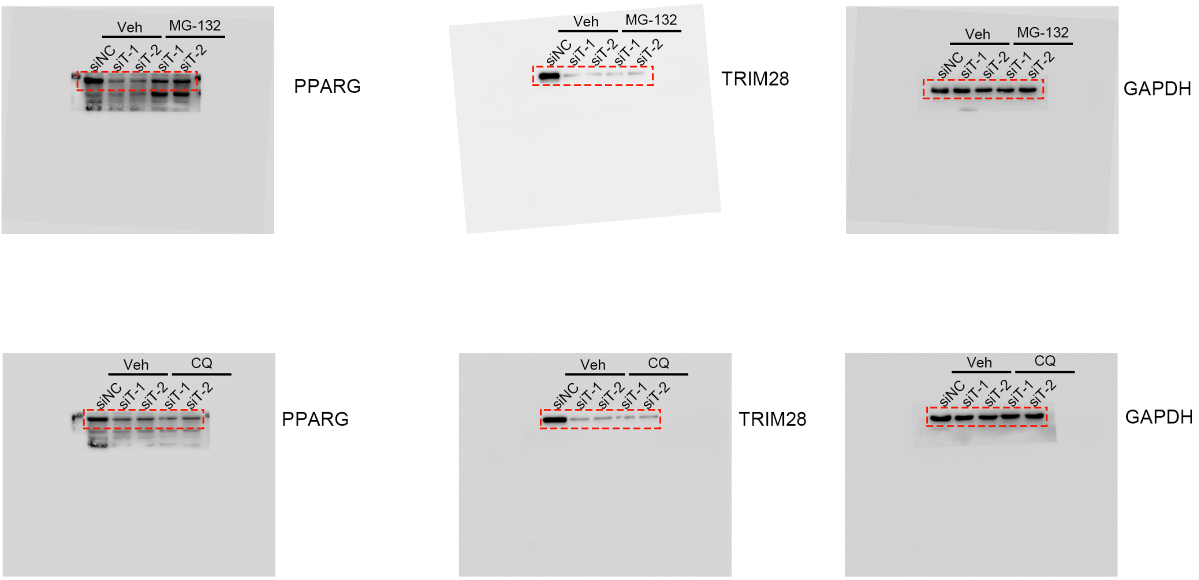

Veh
MG-132
siNC
siT-1
siT-2
siT-1
siT-2
PPARG
Veh
MG-132
siNC
siT-1
siT-2
siT-1
siT-2
TRIM28
Veh
MG-132
siNC
siT-1
siT-2
siT-1
siT-2
GAPDH
Veh
CQ
siNC
siT-1
siT-2
siT-1
siT-2
PPARG
Veh
CQ
siNC
siT-1
siT-2
siT-1
siT-2
TRIM28
Veh
CQ
siNC
siT-1
siT-2
siT-1
siT-2
GAPDH

Figure 5G

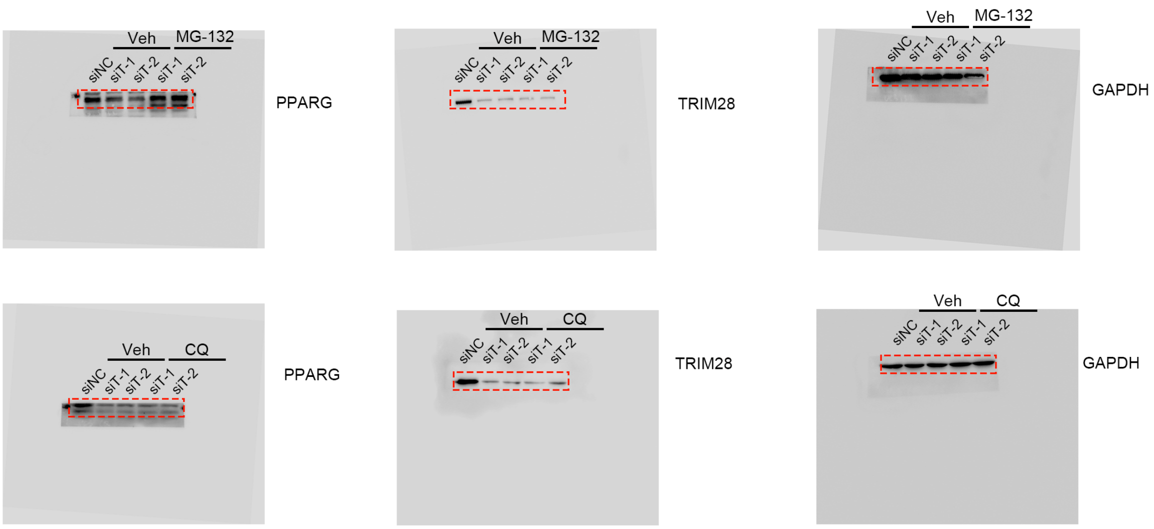

Veh
MG-132
siNC
siT-1
siT-2
siT-1
siT-2
PPARG
TRIM28
GAPDH
Veh
CQ
PPARG
TRIM28
GAPDH

Figure 5H

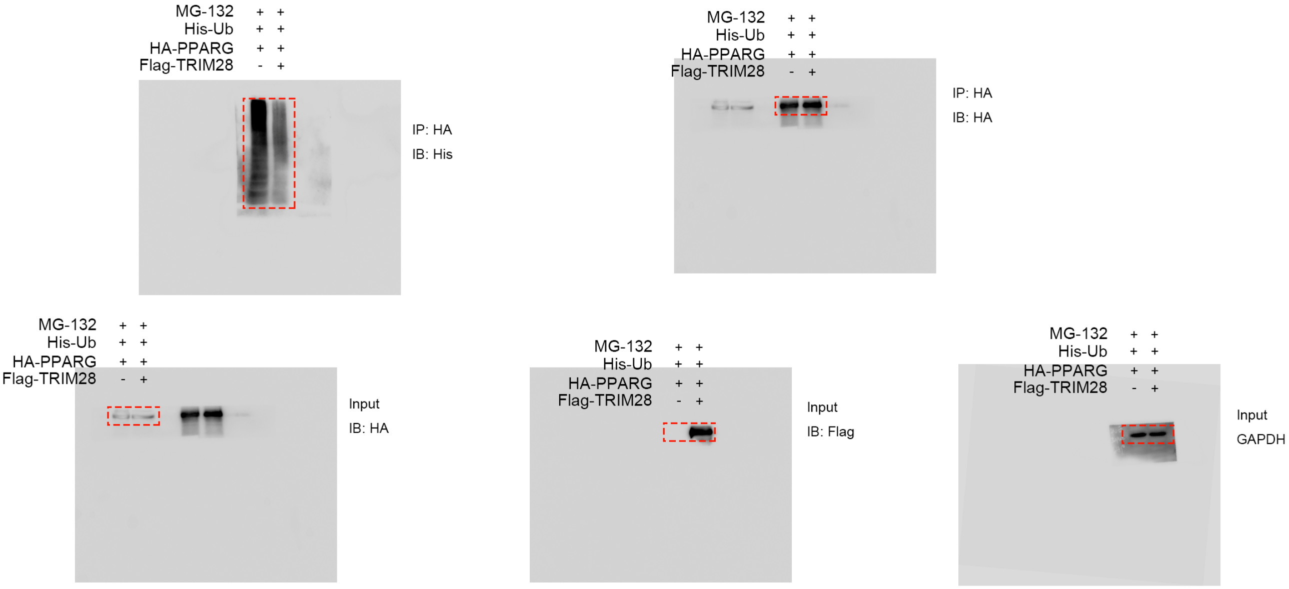
MG-132 + +
His-Ub + +
HA-PPARG + +
Flag-TRIM28 - +
IP: HA
IB: His
MG-132 + +
His-Ub + +
HA-PPARG + +
Flag-TRIM28 - +
IP: HA
IB: HA
MG-132 + +
His-Ub + +
HA-PPARG + +
Flag-TRIM28 - +
Input
IB: HA
MG-132 + +
His-Ub + +
HA-PPARG + +
Flag-TRIM28 - +
Input
IB: Flag
MG-132 + +
His-Ub + +
HA-PPARG + +
Flag-TRIM28 - +
Input
GAPDH

Figure 5I

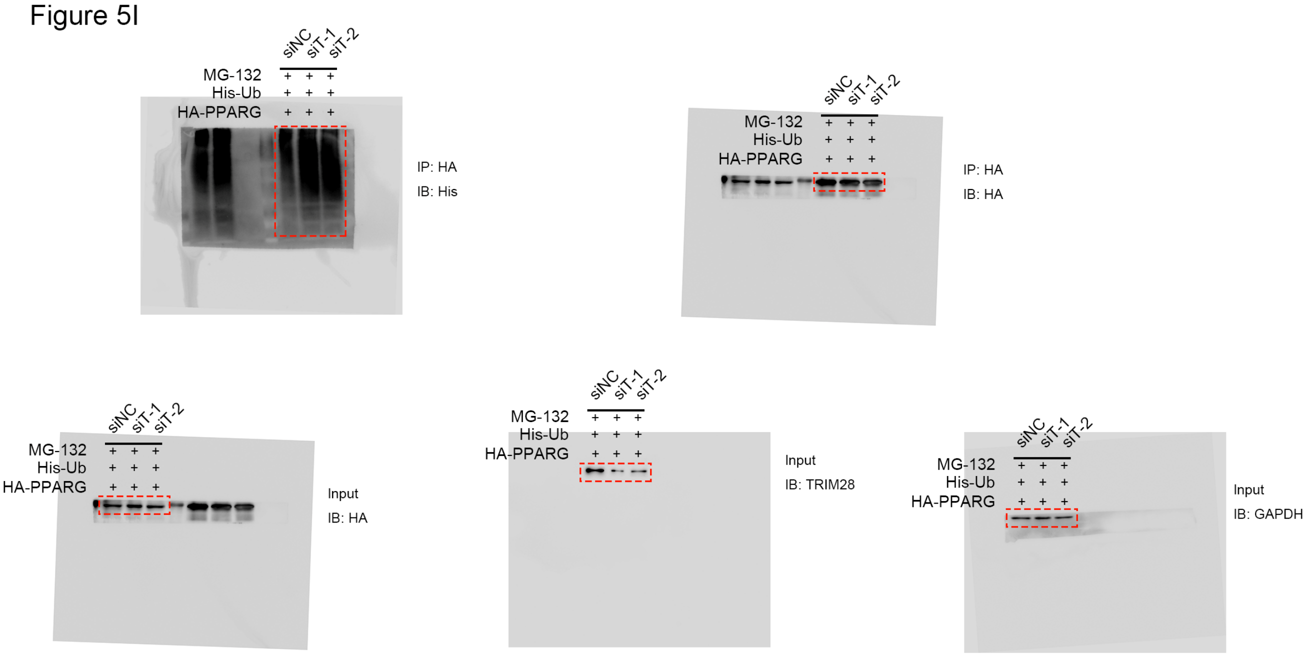

siNC
siT-1
siT-2
MG-132
His-Ub
HA-PPARG
IP: HA
IB: His
IP: HA
IB: HA
Input
IB: HA
Input
IB: TRIM28
Input
IB: GAPDH

Figure 6A

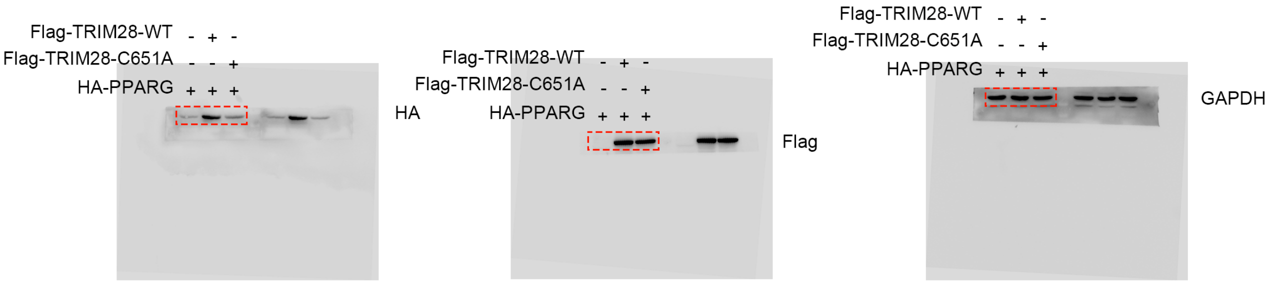
Flag-TRIM28-WT - + -
Flag-TRIM28-C651A - - +
HA-PPARG + + +
HA
Flag-TRIM28-WT - + -
Flag-TRIM28-C651A - - +
HA-PPARG + + +
Flag
Flag-TRIM28-WT - + -
Flag-TRIM28-C651A - - +
HA-PPARG + + +
GAPDH

Figure 6B

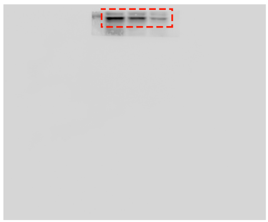

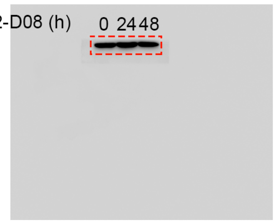

Figure 6C

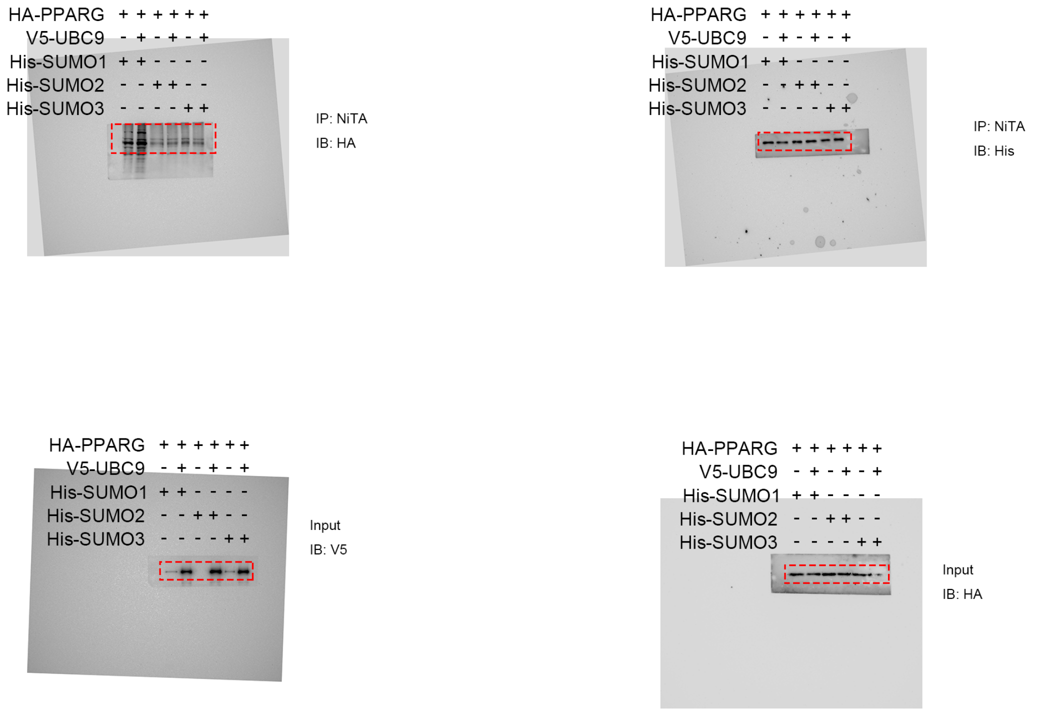
HA-PPARG + + + + + +
V5-UBC9 - + - + - +
His-SUMO1 + + - - - -
His-SUMO2 - - + + - -
His-SUMO3 - - - - + +
IP: NiTA
IB: HA
HA-PPARG + + + + + +
V5-UBC9 - + - + - +
His-SUMO1 + + - - - -
His-SUMO2 - - + + - -
His-SUMO3 - - - - + +
IP: NiTA
IB: His
HA-PPARG + + + + + +
V5-UBC9 - + - + - +
His-SUMO1 + + - - - -
His-SUMO2 - - + + - -
His-SUMO3 - - - - + +
Input
IB: V5
HA-PPARG + + + + + +
V5-UBC9 - + - + - +
His-SUMO1 + + - - - -
His-SUMO2 - - + + - -
His-SUMO3 - - - - + +
Input
IB: HA

Figure 6D

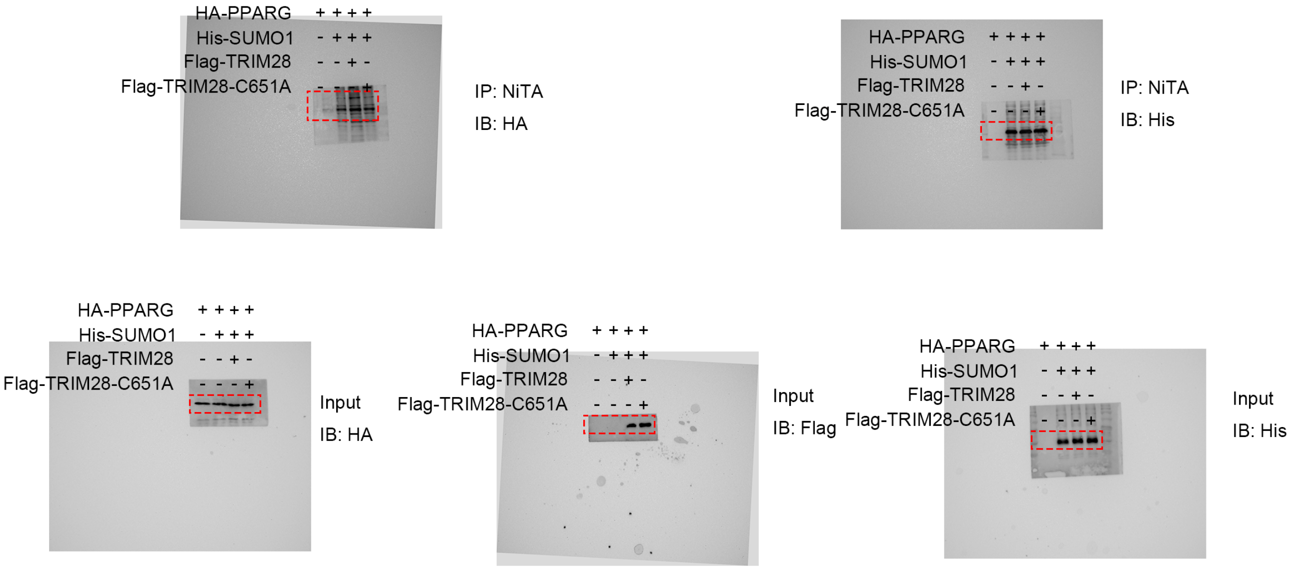
HA-PPARG + + + +
His-SUMO1 - + + +
Flag-TRIM28 - - + -
Flag-TRIM28-C651A - - - +
IP: NiTA
IB: HA
HA-PPARG + + + +
His-SUMO1 - + + +
Flag-TRIM28 - - + -
Flag-TRIM28-C651A - - - +
IP: NiTA
IB: His
HA-PPARG + + + +
His-SUMO1 - + + +
Flag-TRIM28 - - + -
Flag-TRIM28-C651A - - - +
Input
IB: HA
HA-PPARG + + + +
His-SUMO1 - + + +
Flag-TRIM28 - - + -
Flag-TRIM28-C651A - - - +
Input
IB: Flag
HA-PPARG + + + +
His-SUMO1 - + + +
Flag-TRIM28 - - + -
Flag-TRIM28-C651A - - - +
Input
IB: His

Figure 6E

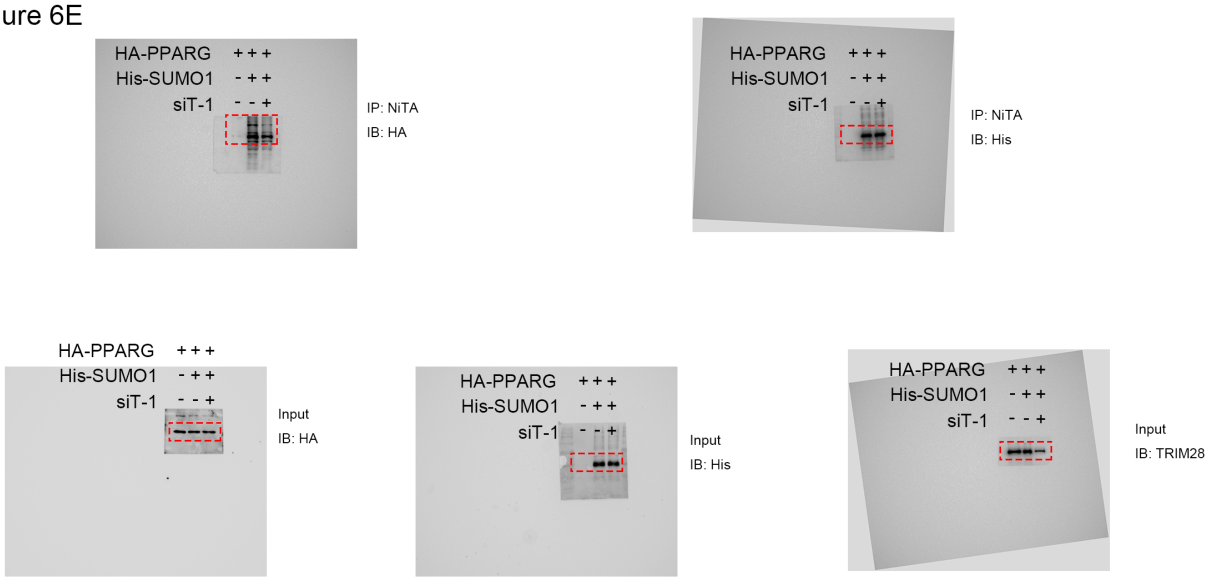
HA-PPARG + + +
His-SUMO1 - + +
siT-1 - - +
IP: NiTA
IB: HA
HA-PPARG + + +
His-SUMO1 - + +
siT-1 - - +
IP: NiTA
IB: His
HA-PPARG + + +
His-SUMO1 - + +
siT-1 - - +
Input
IB: HA
HA-PPARG + + +
His-SUMO1 - + +
siT-1 - - +
Input
IB: His
HA-PPARG + + +
His-SUMO1 - + +
siT-1 - - +
Input
IB: TRIM28

Figure 6F

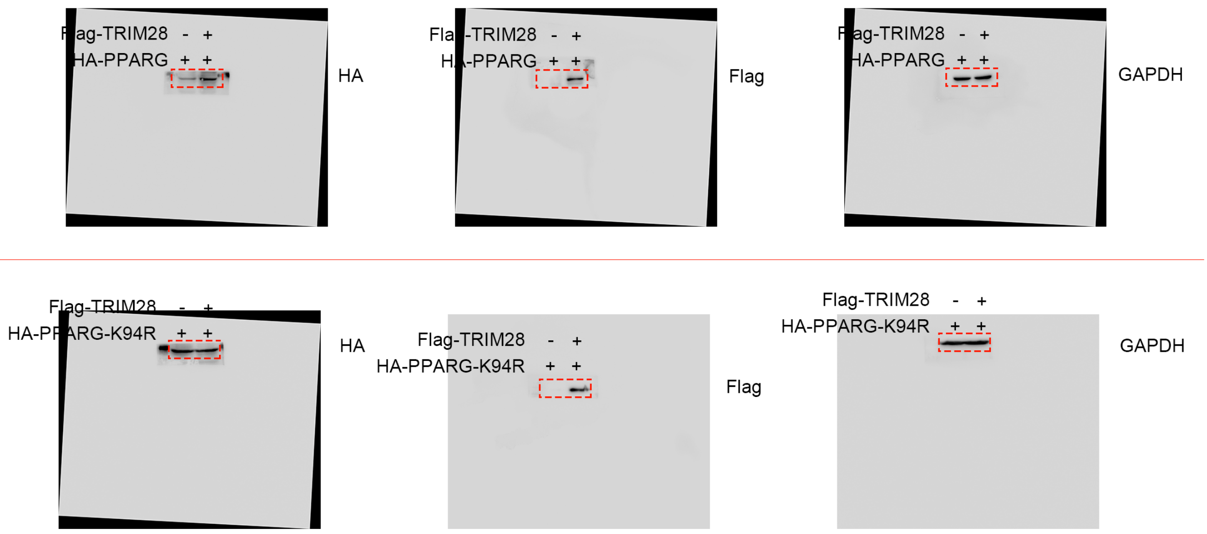

Flag-TRIM28 - +
HA-PPARG + +
HA
Flag
GAPDH
Flag-TRIM28 - +
HA-PPARG-K94R + +
HA
Flag-TRIM28 - +
HA-PPARG-K94R + +
Flag
Flag-TRIM28 - +
HA-PPARG-K94R + +
GAPDH

Figure 6G

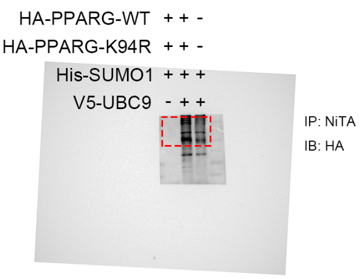
HA-PPARG-WT + + -
HA-PPARG-K94R + + -
His-SUMO1 + + +
V5-UBC9 - + +
IP: NiTA
IB: HA

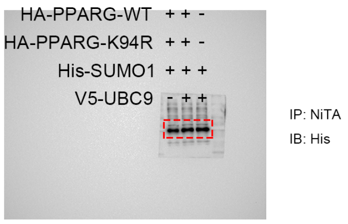
HA-PPARG-WT + + -
HA-PPARG-K94R + + -
His-SUMO1 + + +
V5-UBC9 - + +
IP: NiTA
IB: His

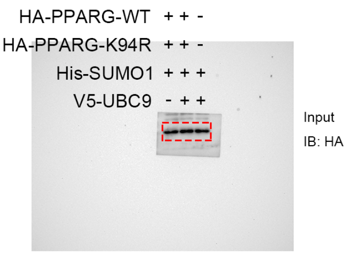
HA-PPARG-WT + + -
HA-PPARG-K94R + + -
His-SUMO1 + + +
V5-UBC9 - + +
Input
IB: HA

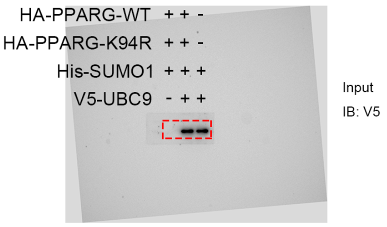
HA-PPARG-WT + + -
HA-PPARG-K94R + + -
His-SUMO1 + + +
V5-UBC9 - + +
Input
IB: V5

Figure 6I

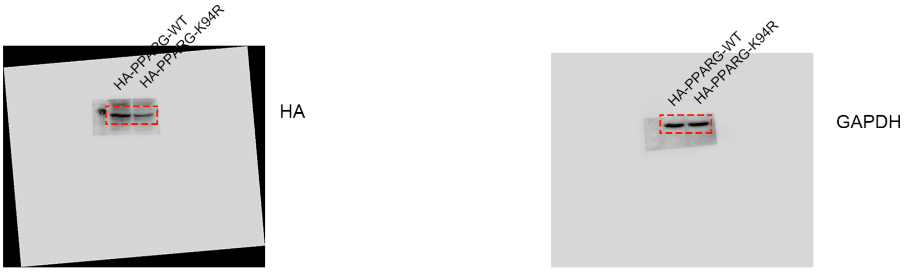

Figure 6J

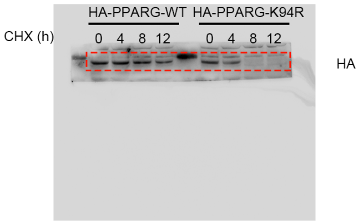

CHX (h)
HA-PPARG-WT
HA-PPARG-K94R
0 4 8 12
0 4 8 12
HA

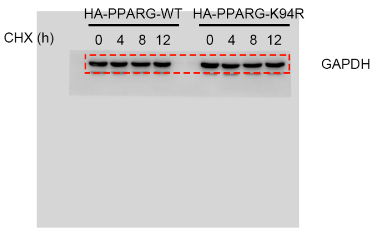

CHX (h)
HA-PPARG-WT
HA-PPARG-K94R
0 4 8 12
0 4 8 12
GAPDH

Figure 6K

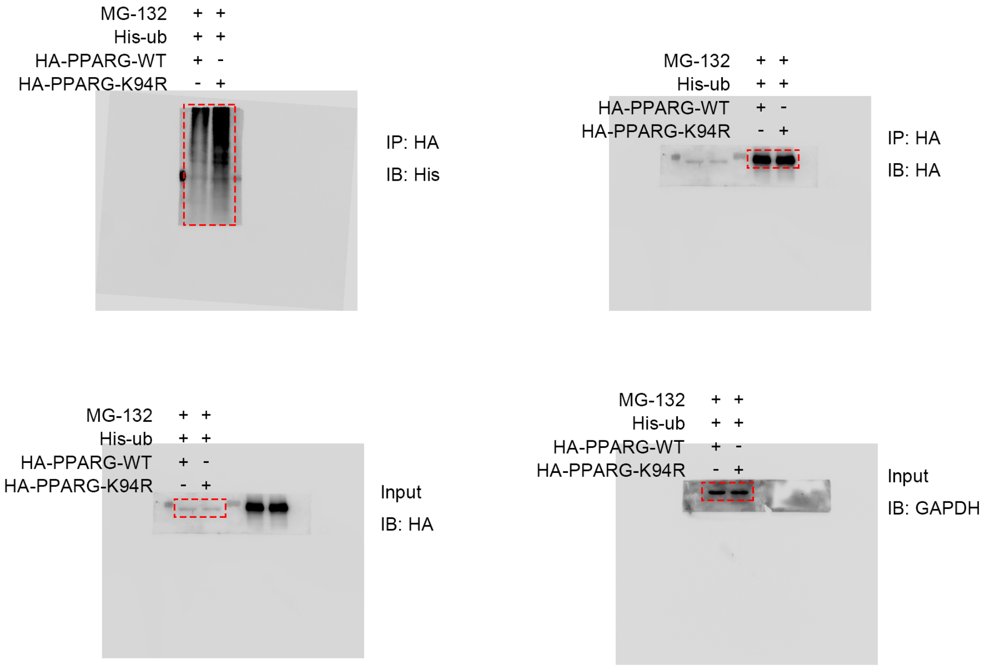
MG-132 + +
His-ub + +
HA-PPARG-WT + -
HA-PPARG-K94R - +
IP: HA
IB: His
MG-132 + +
His-ub + +
HA-PPARG-WT + -
HA-PPARG-K94R - +
IP: HA
IB: HA
MG-132 + +
His-ub + +
HA-PPARG-WT + -
HA-PPARG-K94R - +
Input
IB: HA
MG-132 + +
His-ub + +
HA-PPARG-WT + -
HA-PPARG-K94R - +
Input
IB: GAPDH

Figure 7B

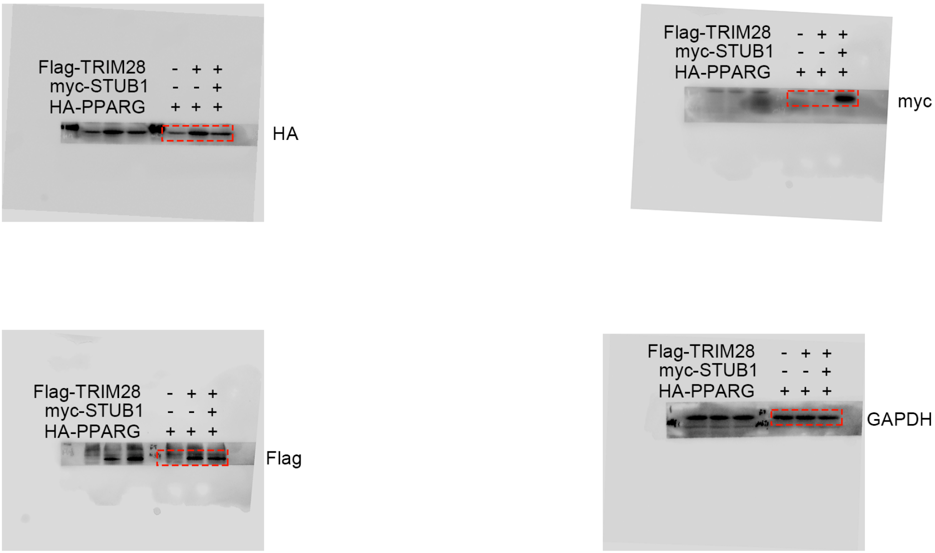

Flag-TRIM28 - + +
myc-STUB1 - - +
HA-PPARG + + +
HA
Flag-TRIM28 - + +
myc-STUB1 - - +
HA-PPARG + + +
myc
Flag-TRIM28 - + +
myc-STUB1 - - +
HA-PPARG + + +
Flag
Flag-TRIM28 - + +
myc-STUB1 - - +
HA-PPARG + + +
GAPDH

Figure 7C

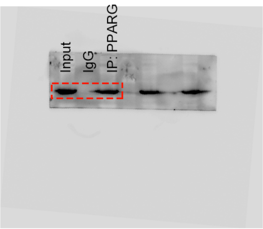

PPARG

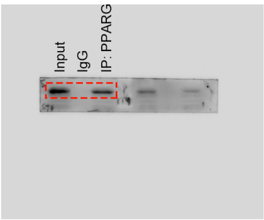

STUB1

Figure 7D

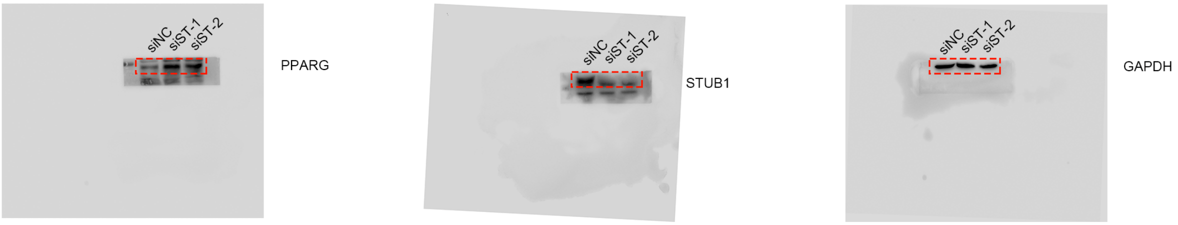
siNC
siST-1
siST-2
PPARG
siNC
siST-1
siST-2
STUB1
siNC
siST-1
siST-2
GAPDH

Figure 7E

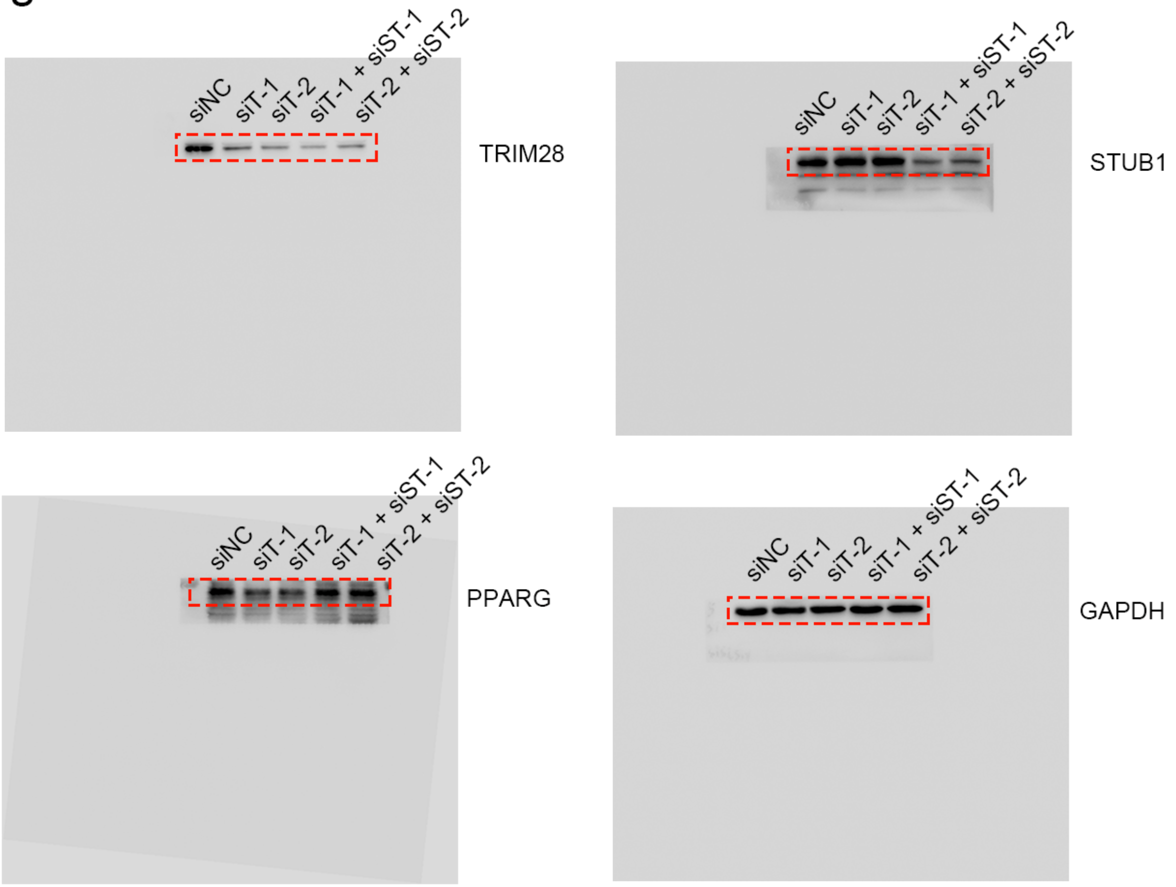
siNC
siT-1
siT-2
siT-1 + siST-1
siT-2 + siST-2
TRIM28
STUB1
PPARG
GAPDH

Figure 7I

myc-STUB1 - + +
HA-PPARG-K94R - - +
HA-PPARG-WT + + -
mg-132 + + +

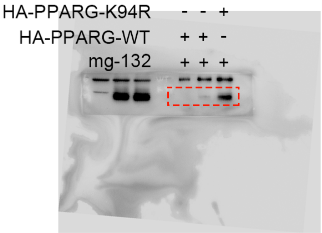

IP: HA
IB: myc

myc-STUB1 - + +
HA-PPARG-K94R - - +
HA-PPARG-WT + + -
mg-132 + + +

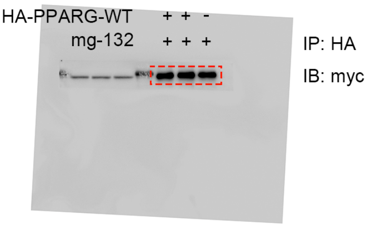

IP: HA
IB: myc

myc-STUB1 - + +
HA-PPARG-K94R - - +
HA-PPARG-WT + + -
mg-132 + + +

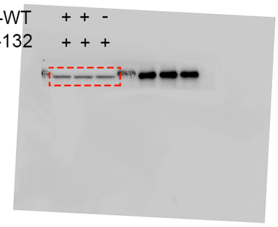

IP: HA
IB: myc

myc-STUB1 - + +
HA-PPARG-K94R - - +
HA-PPARG-WT + + -
mg-132 + + +

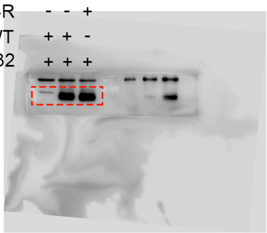

IP: HA
IB: myc

myc-STUB1 - + +
HA-PPARG-K94R - - +
HA-PPARG-WT + + -
mg-132 + + +

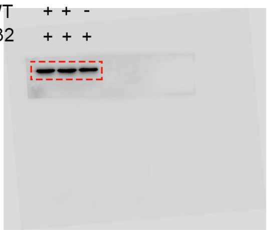

IP: HA
IB: myc

FIgure 7J

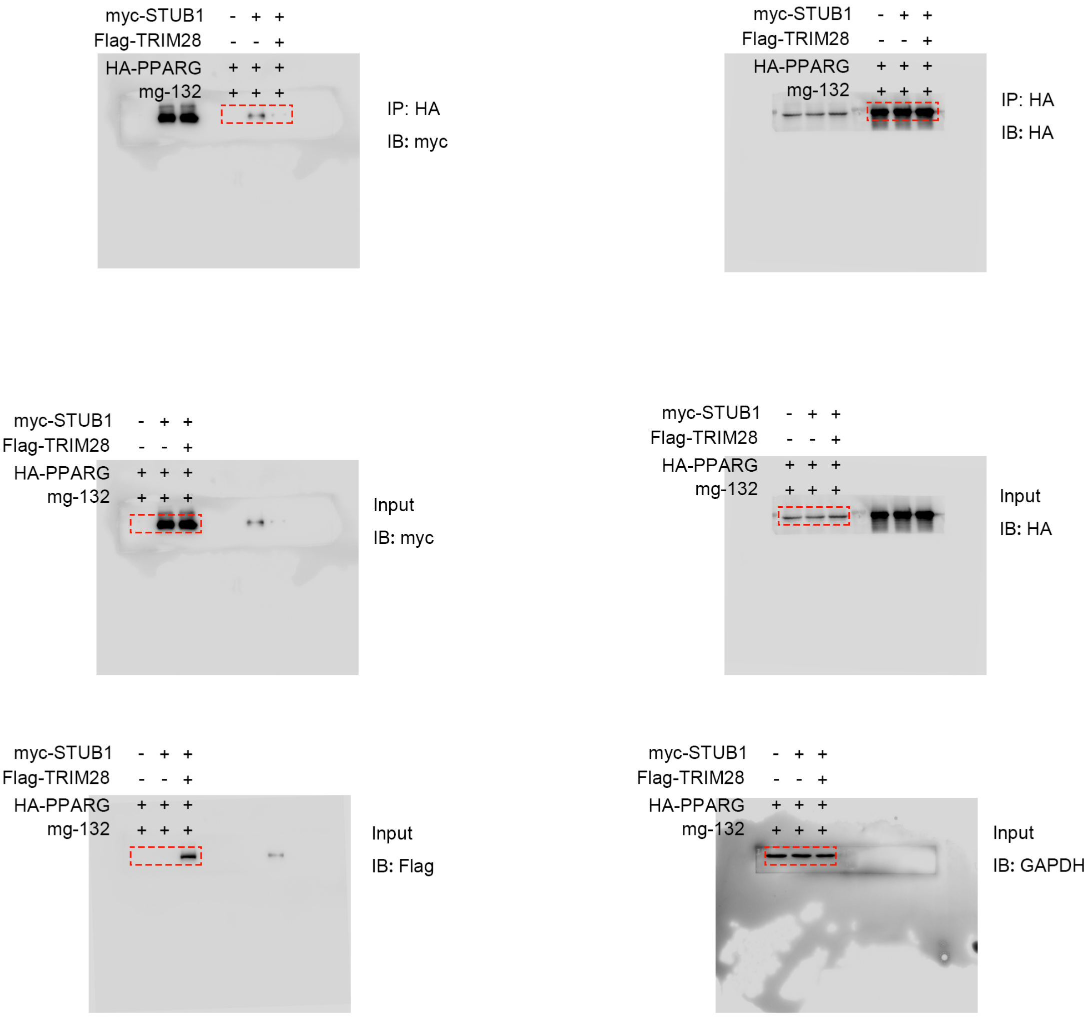
myc-STUB1 - + +
Flag-TRIM28 - - +
HA-PPARG + + +
mg-132 + + +
IP: HA
IB: myc
myc-STUB1 - + +
Flag-TRIM28 - - +
HA-PPARG + + +
mg-132 + + +
IP: HA
IB: HA
myc-STUB1 - + +
Flag-TRIM28 - - +
HA-PPARG + + +
mg-132 + + +
Input
IB: myc
myc-STUB1 - + +
Flag-TRIM28 - - +
HA-PPARG + + +
mg-132 + + +
Input
IB: HA
myc-STUB1 - + +
Flag-TRIM28 - - +
HA-PPARG + + +
mg-132 + + +
Input
IB: Flag
myc-STUB1 - + +
Flag-TRIM28 - - +
HA-PPARG + + +
mg-132 + + +
Input
IB: GAPDH

Figure 7K

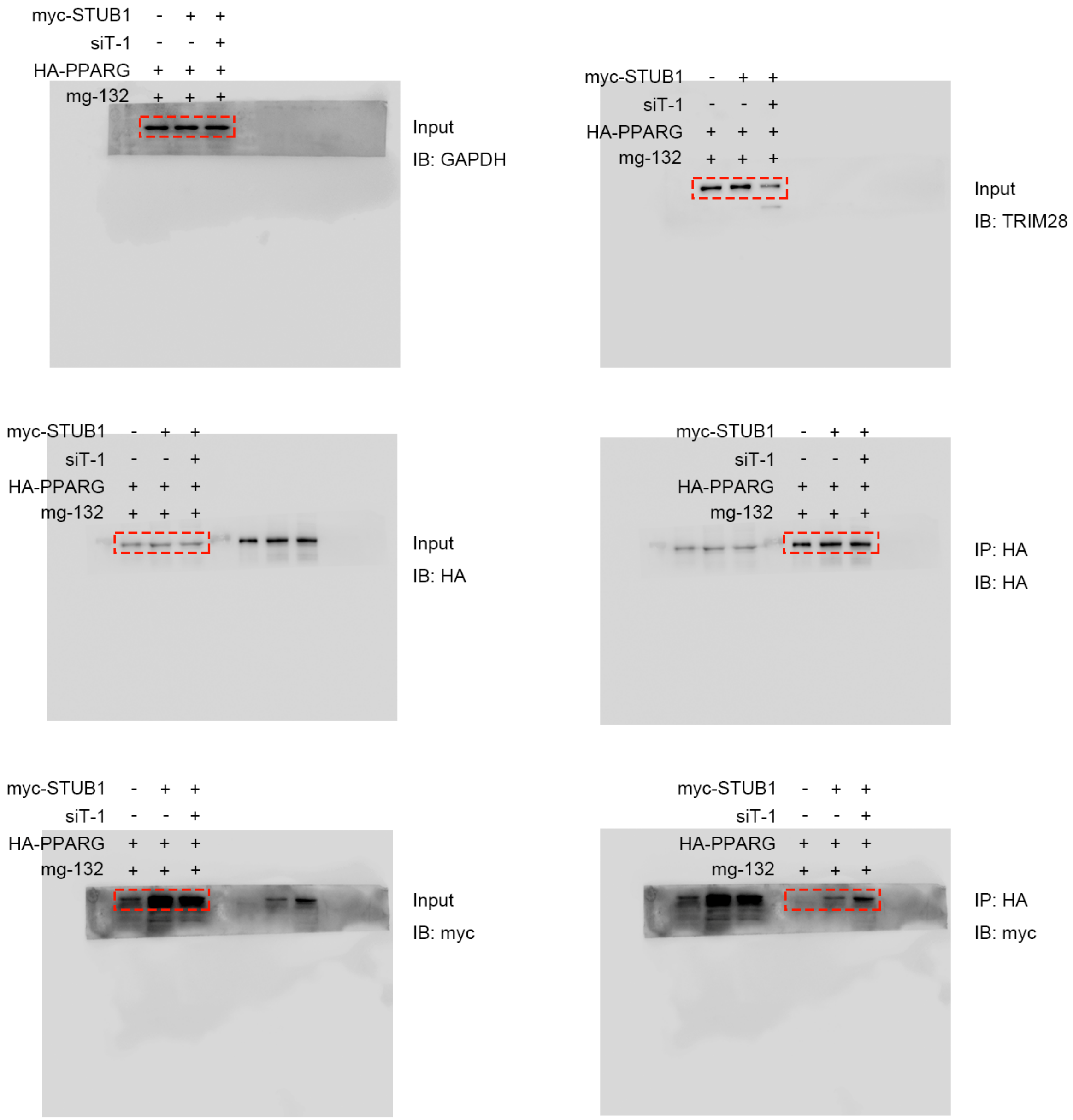

myc-STUB1 - + +
siT-1 - - +
HA-PPARG + + +
mg-132 + + +
Input
IB: GAPDH
myc-STUB1 - + +
siT-1 - - +
HA-PPARG + + +
mg-132 + + +
Input
IB: TRIM28
myc-STUB1 - + +
siT-1 - - +
HA-PPARG + + +
mg-132 + + +
Input
IB: HA
myc-STUB1 - + +
siT-1 - - +
HA-PPARG + + +
mg-132 + + +
IP: HA
IB: HA
myc-STUB1 - + +
siT-1 - - +
HA-PPARG + + +
mg-132 + + +
Input
IB: myc
myc-STUB1 - + +
siT-1 - - +
HA-PPARG + + +
mg-132 + + +
IP: HA
IB: myc

Figure 7L

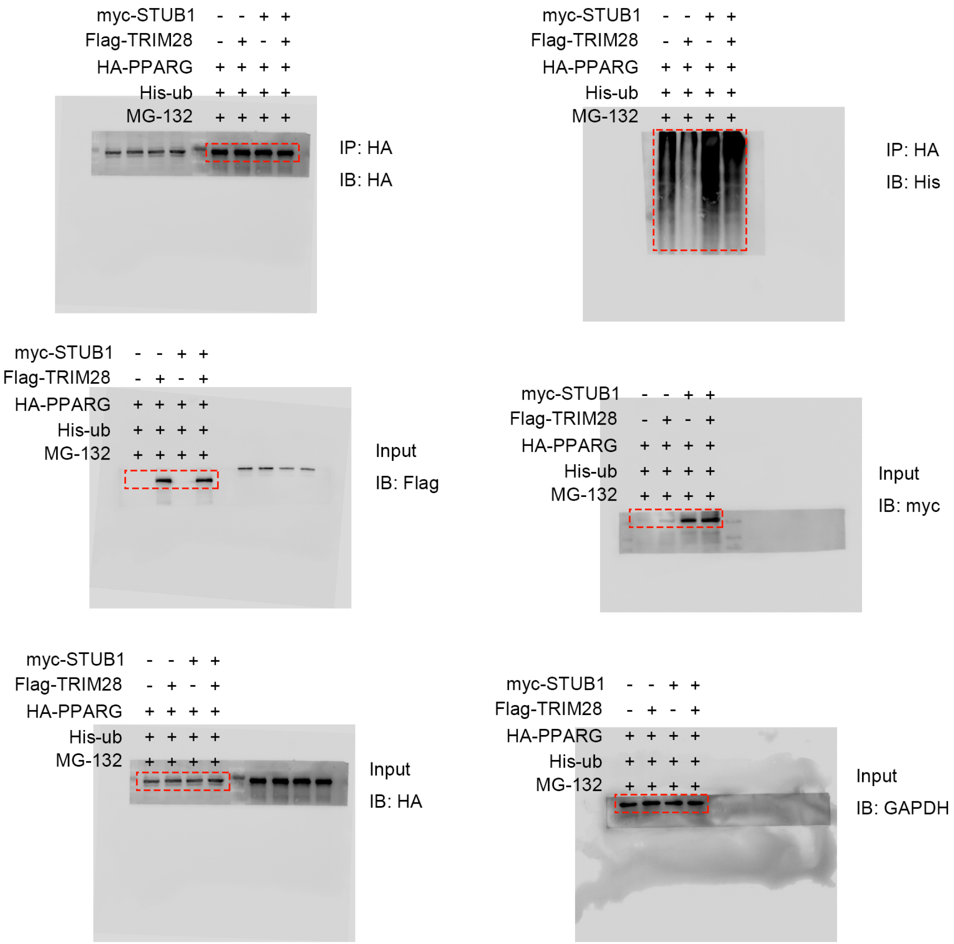
myc-STUB1 - - + +
Flag-TRIM28 - + - +
HA-PPARG + + + +
His-ub + + + +
MG-132 + + + +
IP: HA
IB: HA
myc-STUB1 - - + +
Flag-TRIM28 - + - +
HA-PPARG + + + +
His-ub + + + +
MG-132 + + + +
IP: HA
IB: His
myc-STUB1 - - + +
Flag-TRIM28 - + - +
HA-PPARG + + + +
His-ub + + + +
MG-132 + + + +
Input
IB: Flag
myc-STUB1 - - + +
Flag-TRIM28 - + - +
HA-PPARG + + + +
His-ub + + + +
MG-132 + + + +
Input
IB: myc
myc-STUB1 - - + +
Flag-TRIM28 - + - +
HA-PPARG + + + +
His-ub + + + +
MG-132 + + + +
Input
IB: HA
myc-STUB1 - - + +
Flag-TRIM28 - + - +
HA-PPARG + + + +
His-ub + + + +
MG-132 + + + +
Input
IB: GAPDH

Supplementary Figure 4E

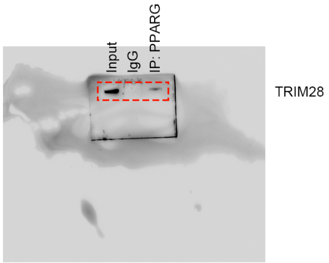

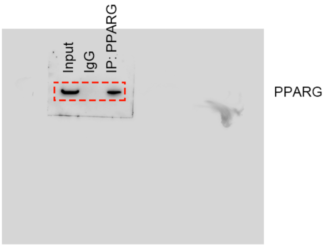

Supplementary Figure 5A

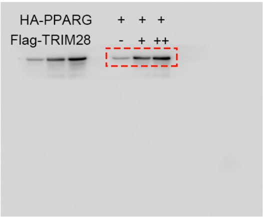

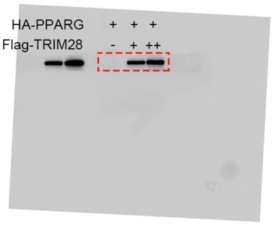

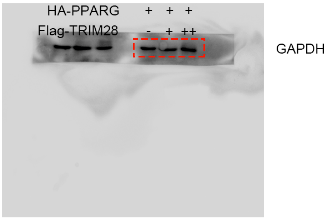

Supplementary Figure 5B

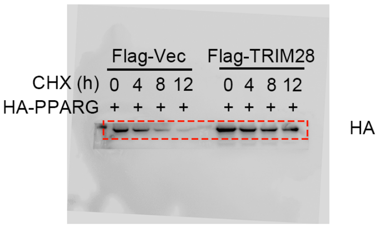

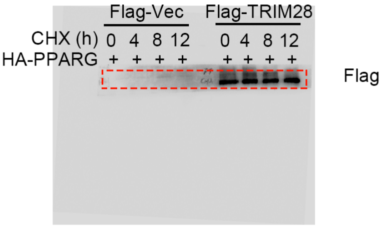

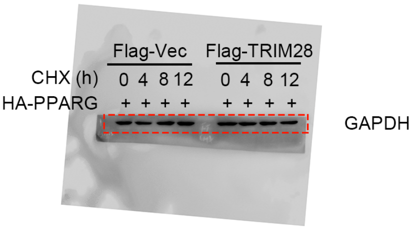

Supplementary Figure 5E

PPARG

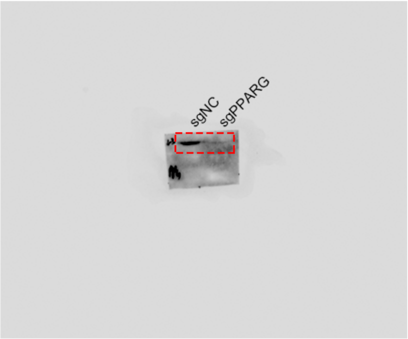

Vinculin

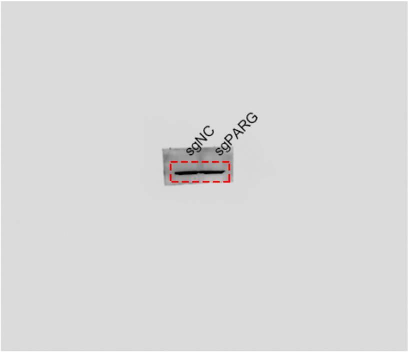

Supplementary Figure 6A

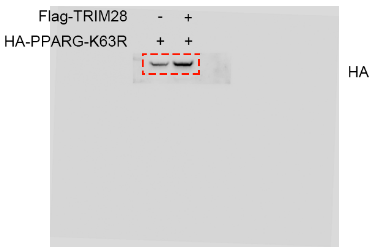

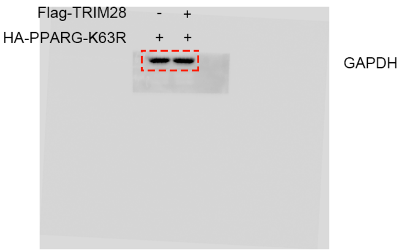

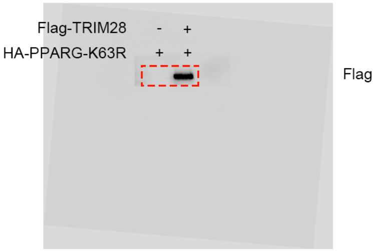

Supplementary Figure 6B

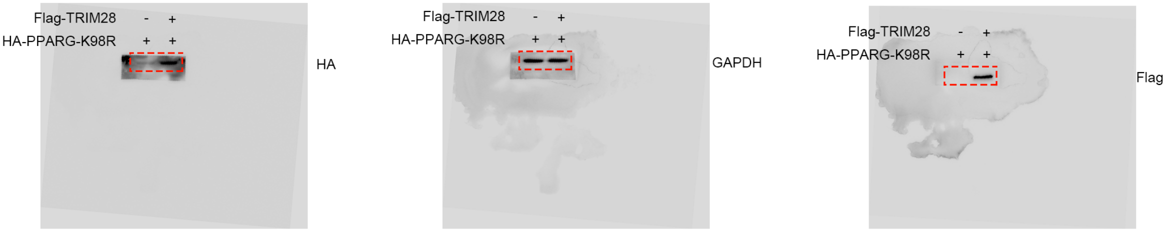

Supplementary Figure 6C

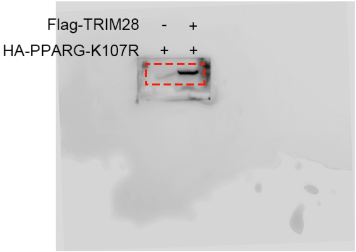

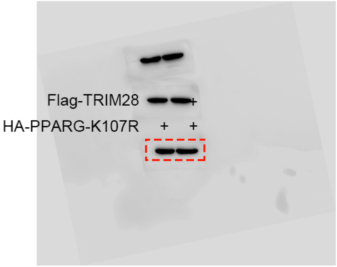

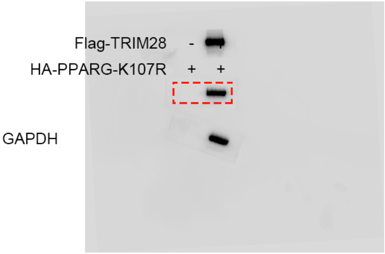

Supplementary Figure 6D

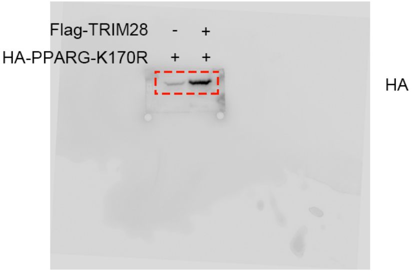

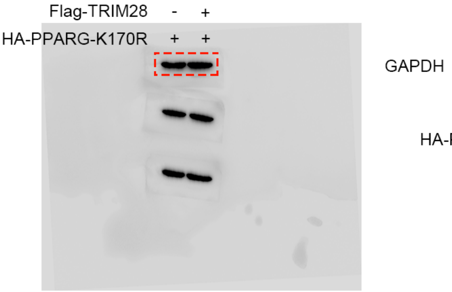

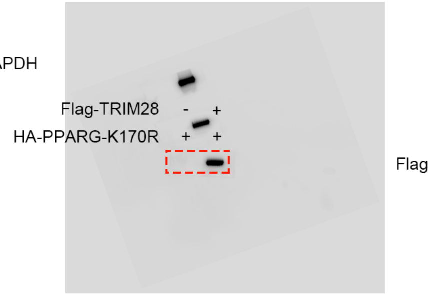

Supplementary Figure 6E

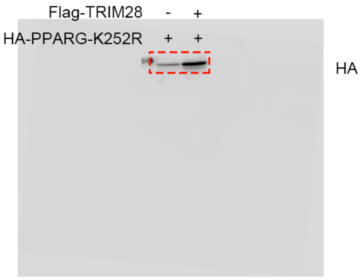

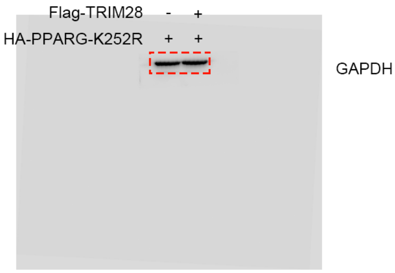

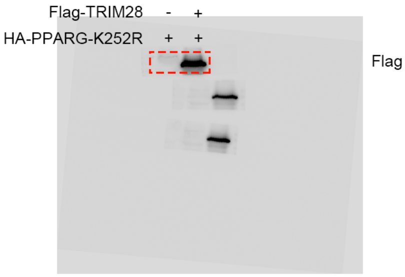

Supplementary Figure 6F

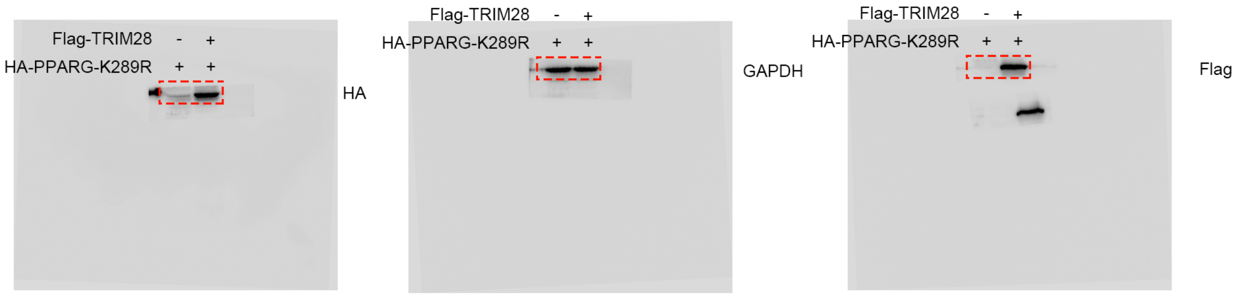

Supplementary Figure 6G

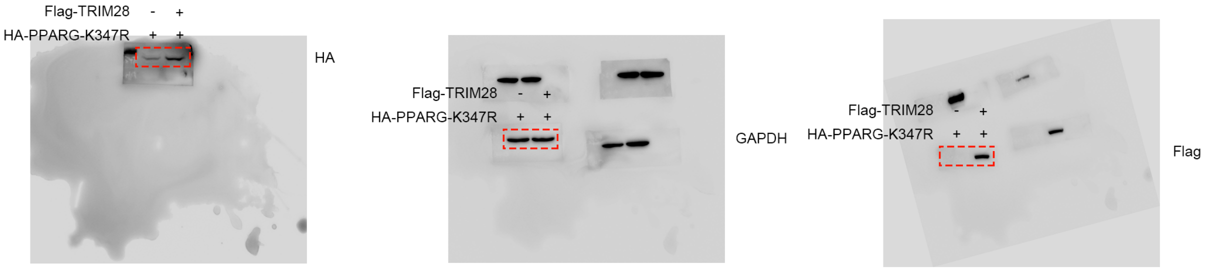

Supplementary Figure 6H

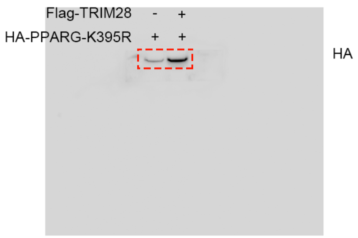

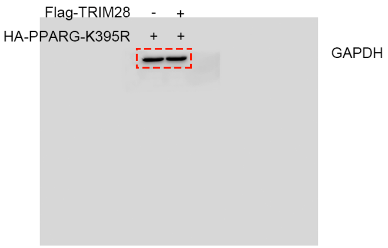

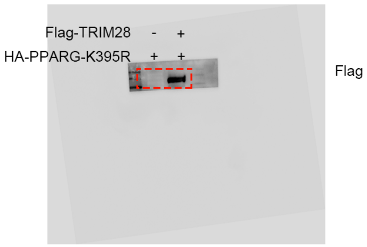

Supplementary Figure 7A

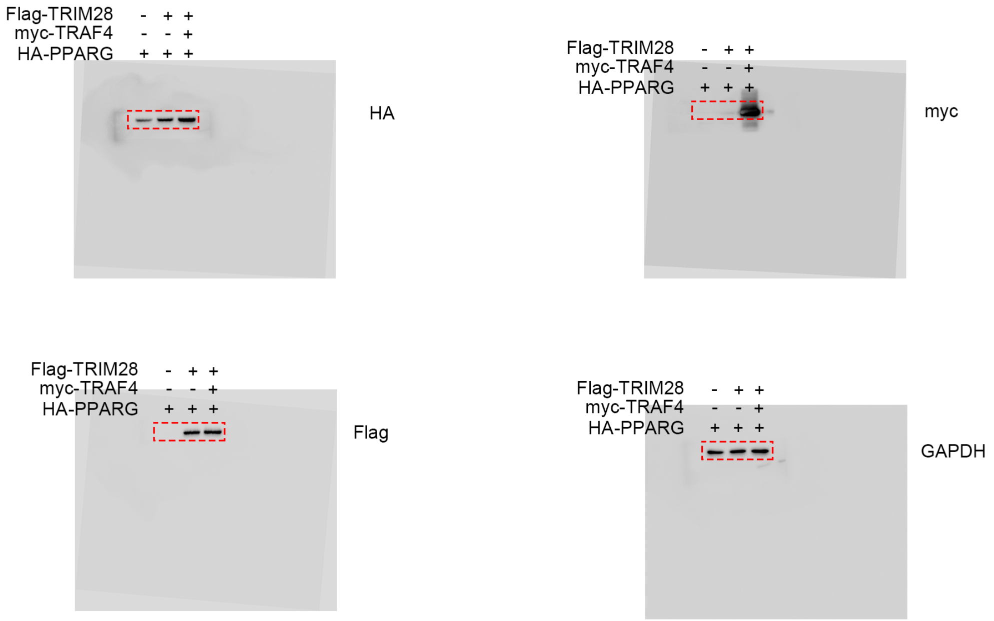

Supplementary Figure 7B

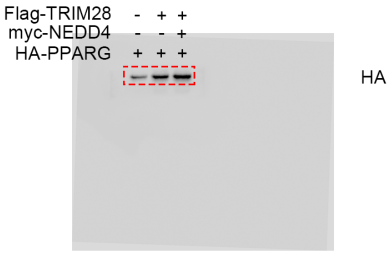

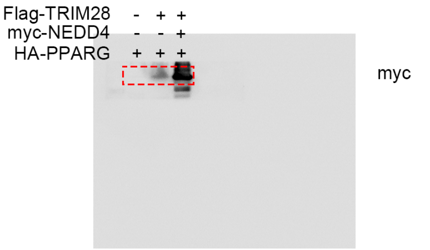

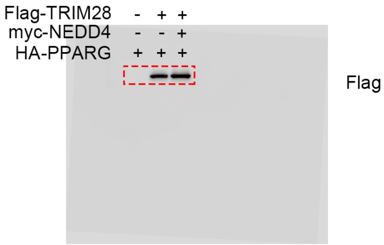

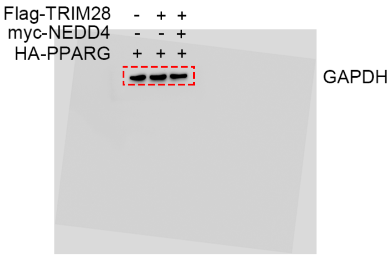

Supplementary Figure 7C

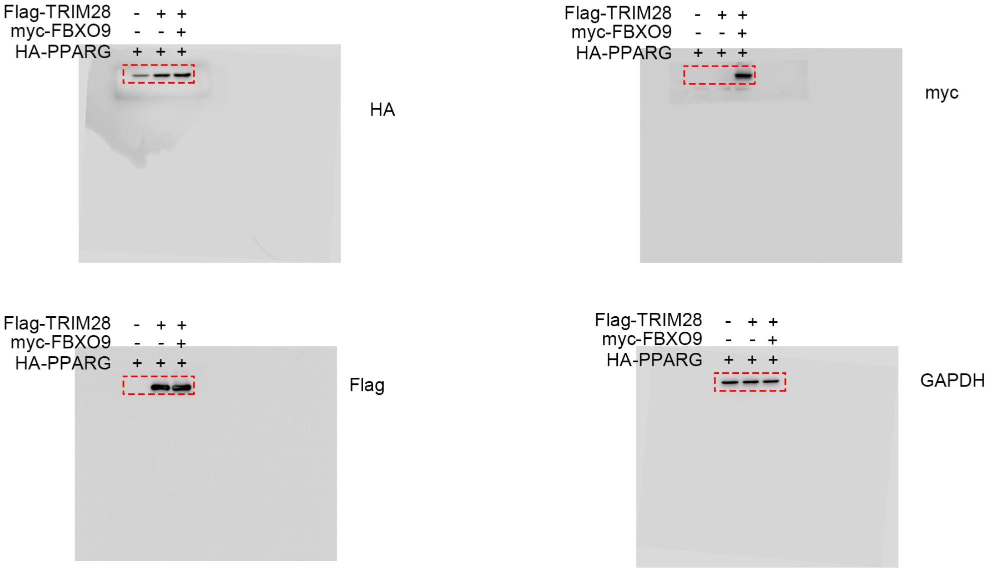

Supplementary Figure 7D

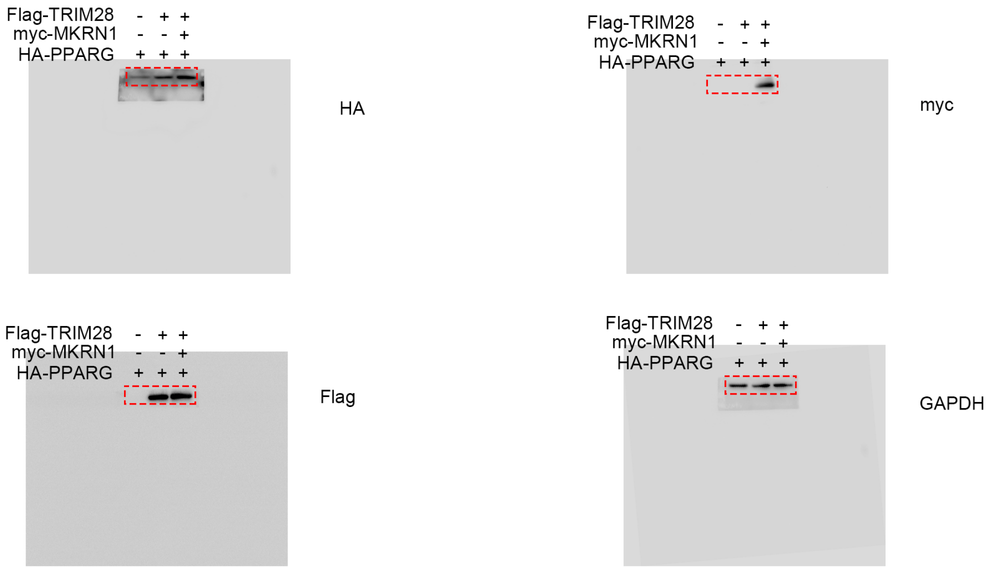

Supplementary Figure 7E

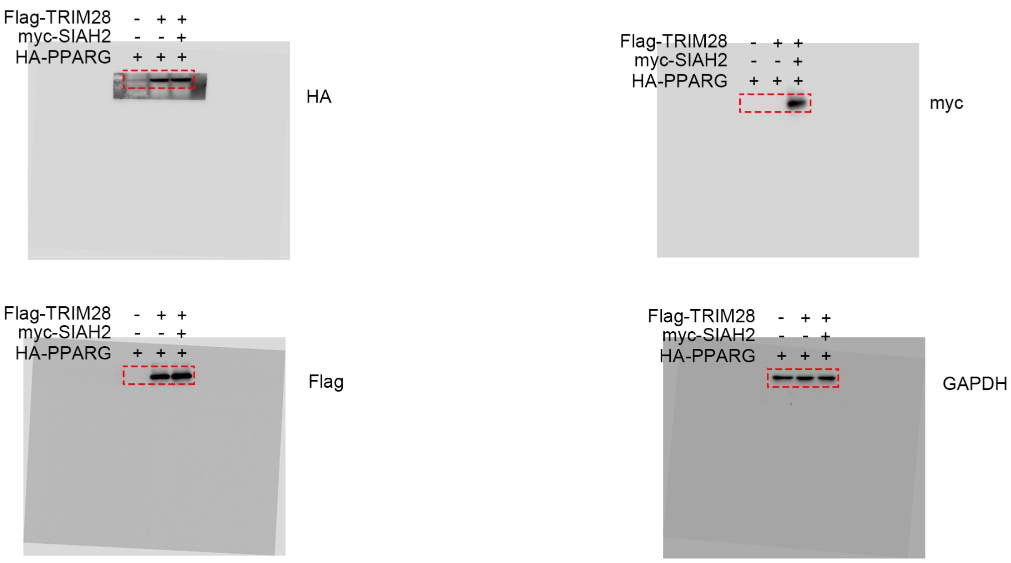

Supplementary Figure 7F

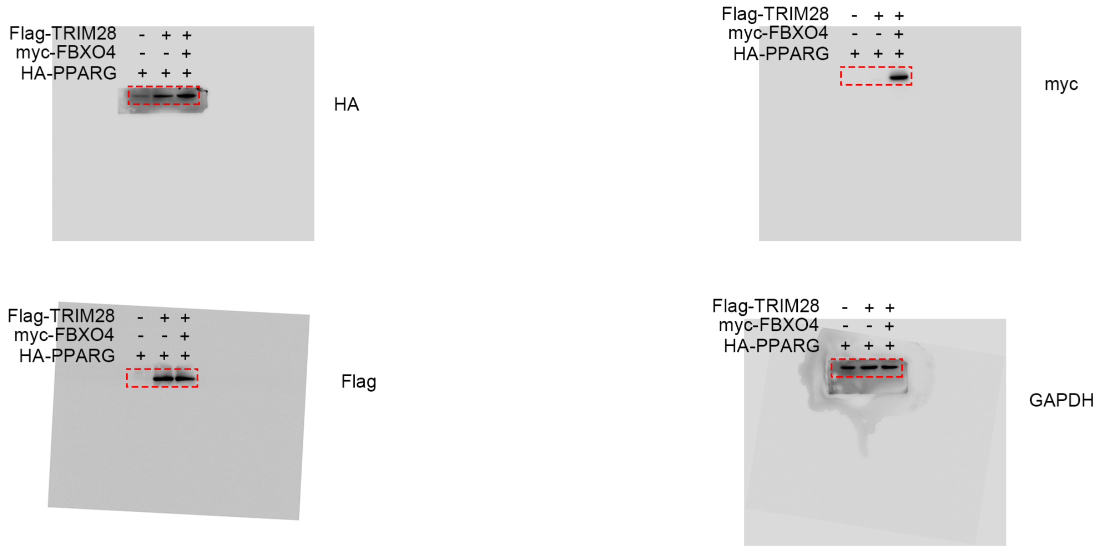

Supplementary Figure 7G

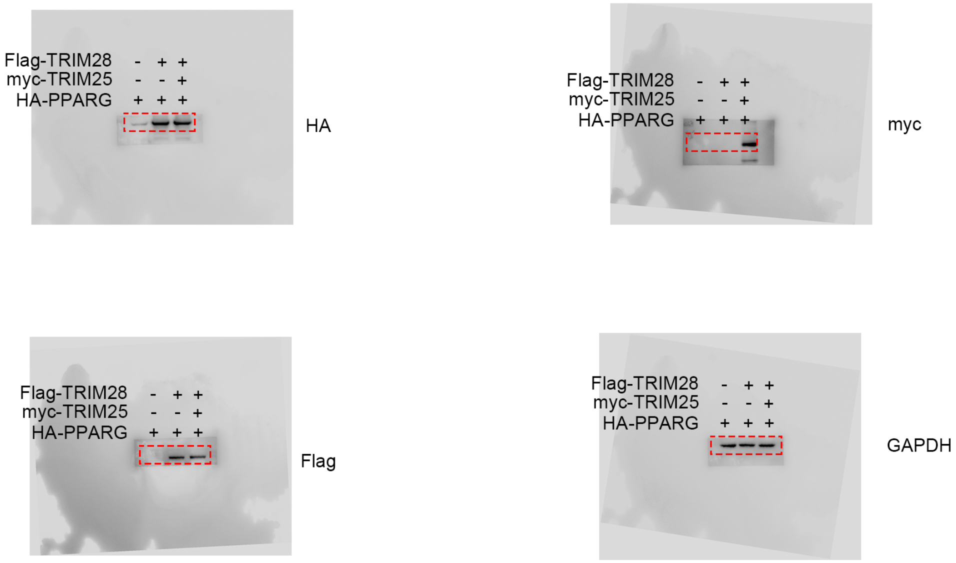

Supplementary Figure 7H

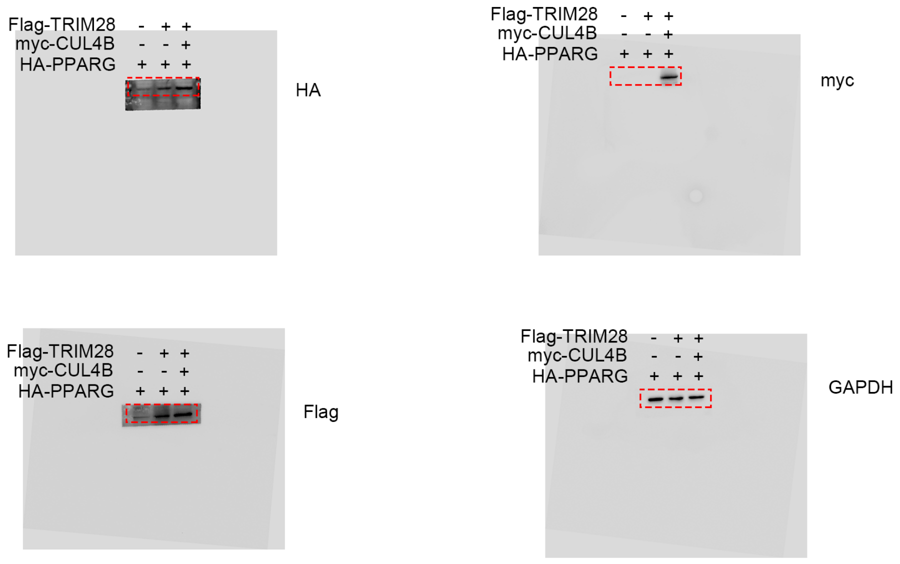

Supplementary Figure 7I

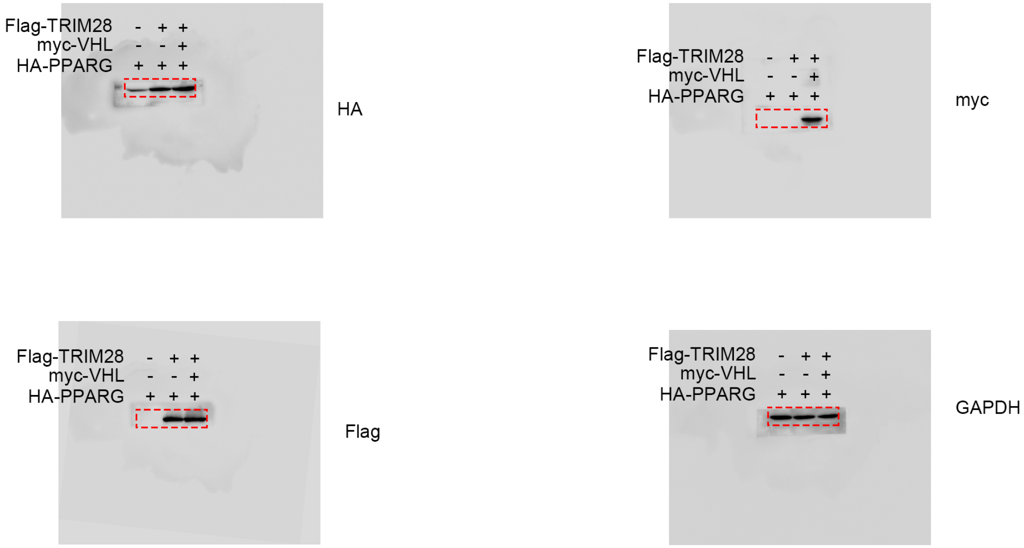

Supplementary Figure 7J

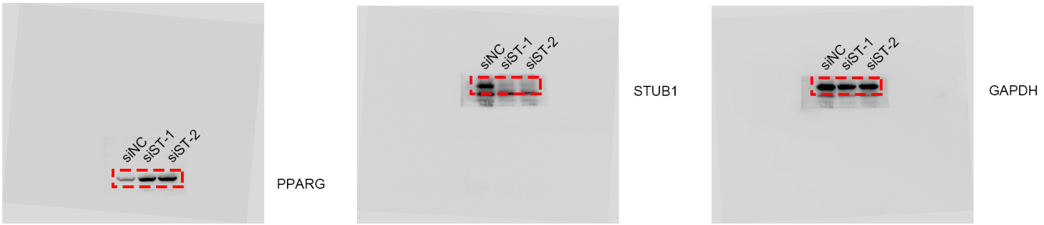

Supplementary Figure 7K

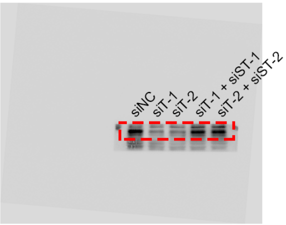

PPARG

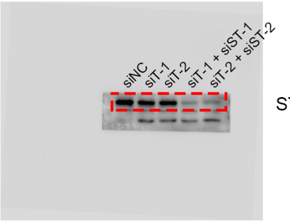

STUB1

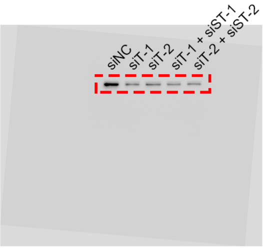

TRIM28

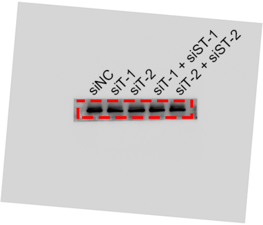

GAPDH

Supplementary Figure 7M

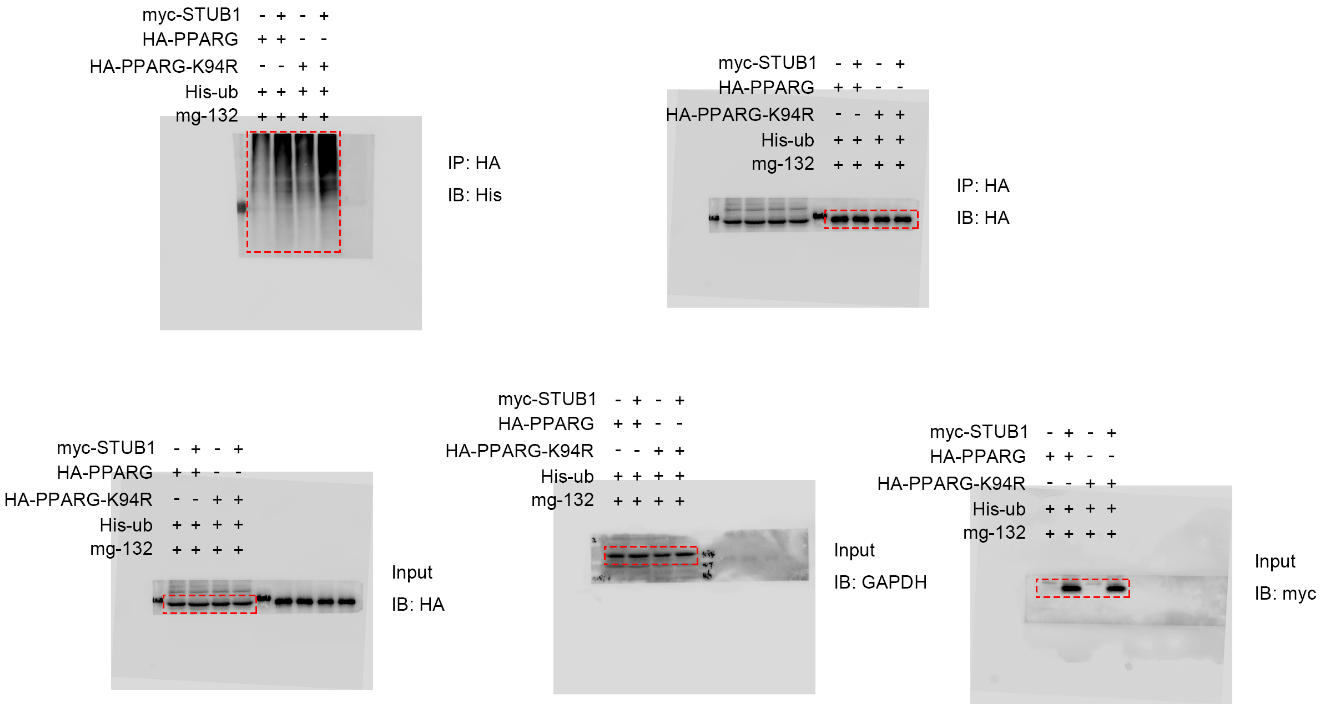

Supplementary Figure 7N

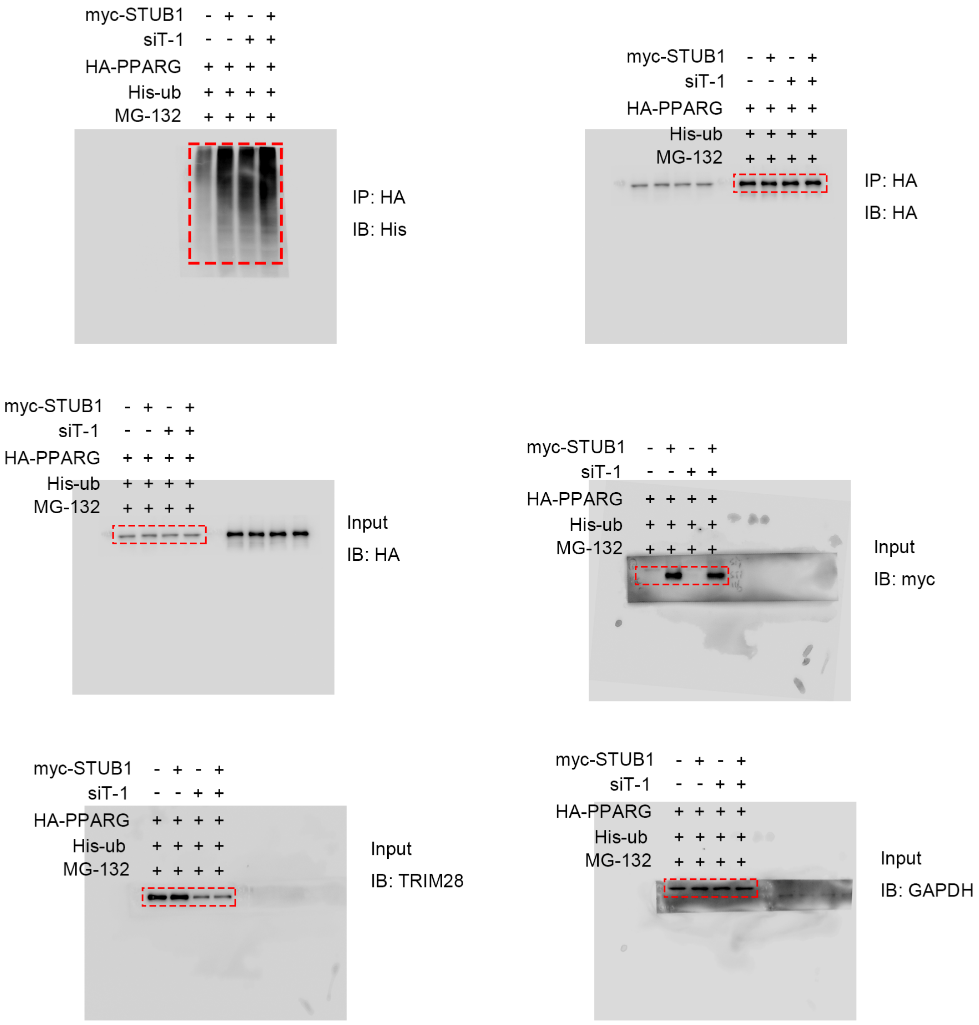

Supplement: Supplementary file 2 — Uncropped data [file 41419_2026_8745_MOESM2_ESM.pdf]
